# Comparative Evaluation of the Antibacterial and Antitumor Activities of 9-Phenylfascaplysin and Its Analogs

**DOI:** 10.3390/md22020053

**Published:** 2024-01-23

**Authors:** Maxim E. Zhidkov, Maria A. Sidorova, Polina A. Smirnova, Oleg A. Tryapkin, Andrey V. Kachanov, Alexey V. Kantemirov, Lyubov G. Dezhenkova, Natalia E. Grammatikova, Elena B. Isakova, Andrey E. Shchekotikhin, Marina A. Pak, Olga N. Styshova, Anna A. Klimovich, Aleksandr M. Popov

**Affiliations:** 1 Department of Chemistry and Materials, Institute of High Technologies and Advanced Materials, FEFU Campus, Far Eastern Federal University, Ajax Bay 10, Russky Island, 690922 Vladivostok, Russia; 2 Laboratory of Chemical Transformation of Antibiotics, Gause Institute of New Antibiotics, 119021 Moscow, Russia; 3 Departments of Biotechnology and Marine Natural Compounds Chemistry, G.B. Elyakov Pacific Institute of Bioorganic Chemistry, Far Eastern Branch of The Russian Academy of Sciences, 690922 Vladivostok, Russiaannaklim_1991@mail.ru (A.A.K.);

**Keywords:** fascaplysin derivatives, antibacterial activity, antiproliferative activity, antitumor activity, 9-phenylfascaplysin

## Abstract

Based on the results of our own preliminary studies, the derivative of the marine alkaloid fascaplysin containing a phenyl substituent at C-9 was selected to evaluate the therapeutic potential in vivo and in vitro. It was shown that this compound has outstandingly high antimicrobial activity against Gram-positive bacteria, including antibiotic-resistant strains in vitro. The presence of a substituent at C-9 of the framework is of fundamental importance, since its replacement to neighboring positions leads to a sharp decrease in the selectivity of the antibacterial action, which indicates the presence of a specific therapeutic target in bacterial cells. On a model of the acute bacterial sepsis in mice, it was shown that the lead compound was more effective than the reference antibiotic vancomycin seven out of nine times. However, ED_50_ value for 9-phenylfascaplysin (**7**) was similar for the unsubstituted fascaplysin (**1**) in vivo, despite the former being significantly more active than the latter in vitro. Similarly, assessments of the anticancer activity of compound **7** against various variants of Ehrlich carcinoma in mice demonstrated its substantial efficacy. To conduct a structure–activity relationship (SAR) analysis and searches of new candidate compounds, we synthesized a series of analogs of 9-phenylfascaplysin with varying aryl substituents. However, these modifications led to the reduced aqueous solubility of fascaplysin derivatives or caused a loss of their antibacterial activity. As a result, further research is required to explore new avenues for enhancing its pharmacokinetic characteristics, the modification of the heterocyclic framework, and optimizing of treatment regimens to harness the remarkable antimicrobial potential of fascaplysin for practical usage.

## 1. Introduction

**Figure 1:**
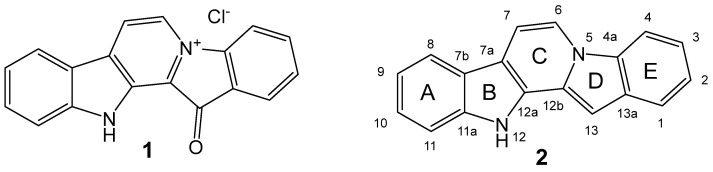
Structures of fascaplysin (**1**) and 12*H*-pyrido[1,2*-a*:3,4-*b′*]diindole (**2**).

In the post-antibiotics era, the spreading of multi-drug-resistant (MDR) pathogenic bacteria poses a great challenge for the global health system [1]. While antibiotics have played a critical role in saving millions of lives, their extensive use has led to the emergence and rapid dissemination of antibiotic resistance [2,3,4]. Notably, the majority of antibiotics currently used in practice or under clinical development belong to a limited set of chemical classes [5]. Hence, there is a pressing need to explore new molecular frameworks exhibiting potent antibacterial properties or targeting novel biological mechanisms [6]. In this context, marine-derived compounds have gained prominence as valuable sources for the development of innovative antimicrobial agents [7]. One of these promising compounds is the red pigment fascaplysin (**1**, Figure 1), isolated from the marine sponge *Fascaplysinopsis* sp. [8]. It was the first representative of the group of marine alkaloids containing a 12*H*-pyrido[1,2-*a*:3,4-*b*′]diindole ring system (**2**). This group also includes homofascaplysins A–C and their brominated analogs [9]. The spectrum of biological activity of fascaplysin (**1**) includes antitumor, antibacterial, antifungal, antiviral, and antimalarial actions [8,9,10].

**Figure 2:**
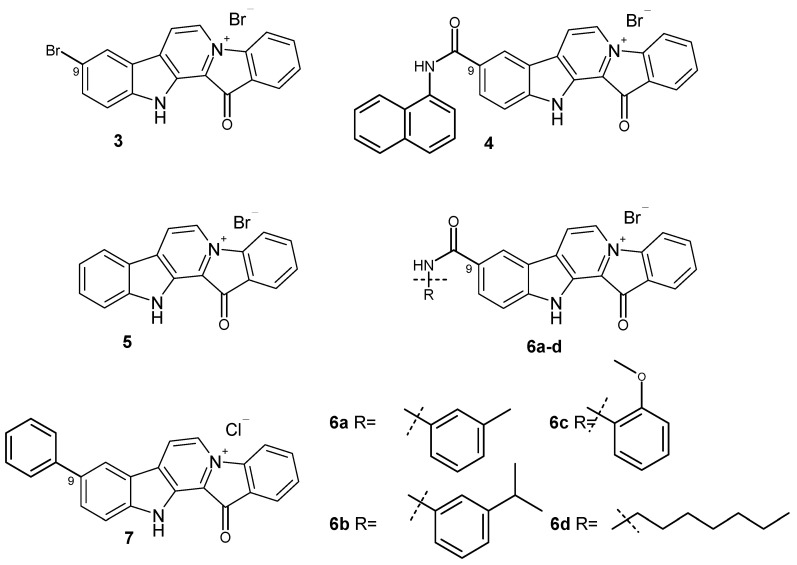
The structures of fascaplysin derivatives **3**–**7** with high antibacterial activity.

Notably, some derivatives of fascaplysin have therapeutic potencies compared to the parental alkaloid [11]. Most of them have been studied for their potential use as antitumor agents. In the same time, recently, the antibacterial activities of some of fascaplysin derivatives substituted at C-9 against methicillin-resistant *Staphylococcus aureus* (MRSA) were described [12]. The results showed that fascaplysin derivatives **3** and **4** exhibited higher activity against MRSA ATCC43300 (Figure 2). Moreover, several compounds are up to 10 times more potent than vancomycin (an antibiotic commonly used to treat of MRSA infections). These derivatives of fascaplysin also potently inhibited MRSA biofilm formation in vitro and had high antibacterial efficacy in vivo. It is also important to note that the antimicrobial activity of these compounds depends on the nature of the counter ion used. Even in the case of unsubstituted fascaplysin, the replacement of the chloride anion by the bromide anion (**5**) leads to an approximately twofold increase in the antibacterial activity. It is noteworthy that compound **3** was first obtained over twenty years ago while studying the chemical properties of fascaplysin [13]. Compounds related to amide **4** were previously investigated as potent multi-targeted agents for the treatment of Alzheimer’s disease [14].

Further evaluation of fascaplysin derivatives **6a**–**6d** showed that these amphiphilic compounds with high antibacterial activity have low hemolytic activity and cytotoxicity to mammalian cells. Preliminary mechanistic exploration suggests that compounds **6a**–**6d** are potent FtsZ inhibitors, which block the GTPase activity of FtsZ (filamentous temperature-sensitive mutant Z), leading to the inhibition of bacterial cell division [15]. The FtsZ protein, playing a key role in bacterial cell division, has recently been identified as an attractive target in the development of new antibacterial drugs [16,17,18,19,20]. At the same time, 9-phenylfascaplysin (**7**) and a series of its derivatives can be considered as analogs of FtsZ inhibitor **6a** (Figure 2) [21]. The remarkable antibacterial properties of these series show promise for the further in-depth study of compounds **6** and **7** and their analogs. Having obtained 9-phenylfascaplysin independently from the authors of [21], we set out to study the therapeutic potential of this compound and its closest derivatives.

In this work, we present, for the first time, the results of a comprehensive study of 9-phenylfascaplysin (**7**). At the initial stage of this study, we developed a scheme for the synthesis of the target compound, its isomers at different positions of the fascaplysin skeleton, and a number of derivatives for subsequent assessment of the effects of structural changes on the target biological activity. Then, the antimicrobial activities of the resulting compounds against a wide range of pathogenic bacteria and their antiproliferative activities against several tumor cells were assessed. At the next stage, these types of activities were studied in vivo. Finally, the acute toxicity of the most promising derivative in this series was studied.

## 2. Results and Discussion

### 2.1. Preparation of 9-Phenylfascaplysin, Its Isomers, and Some Analogs

**Scheme 1:**
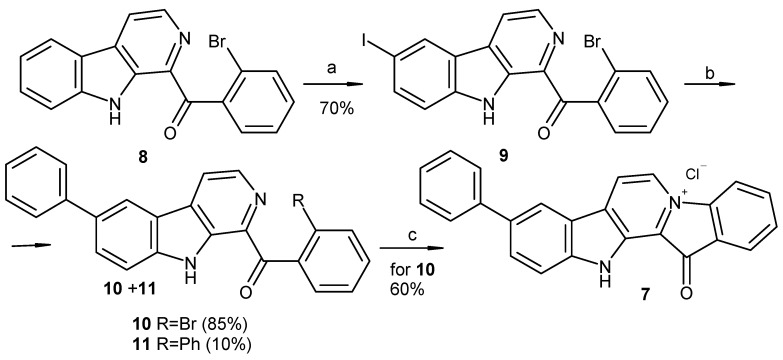
Reagents and conditions: (a) I_2_ (1.1 equiv.), TsOH (10 equiv.), DMSO, 110 °C, and 1 h; (b) PhB(OH)_2_ (1.2 equiv.), toluene, 2M aq. Na_2_CO_3_, Pd[PPh_3_]_4_ (0.025 equiv.), argon, 90 °C, and 7 h; (c) 220 °C, 40 min, and HCl (aq).

To date, more than ten methods used to synthesize fascaplysin and its derivatives and analogs have been reported [22,23,24,25,26,27,28,29,30,31]. The two-step synthesis suggested by Zhu et al. is the most suitable for preparation of fascaplysin derivatives from substituted tryptamines and acetophenones [30]. Our investigation revealed that the direct iodination of β-carboline **8** at C-6 occurred during its cyclization from tryptamine and 2′-bromoacetophenone, using of an excess of molecular iodine. We then tried to use this finding to obtain 9-phenylfascaplysin (Scheme 1). Given that the subsequent high-temperature quaternization step exhibited superior reactivity with the *o*-bromobenzoyl-β-carboline derivative compared to its chloro or fluoro analogs, we tried to use a selective cross-coupling reaction involving the iodine atom of substrate **9**, which also contained a bromine atom in the benzoyl residue. Attempts to perform cross-coupling of **9** with phenylboronic acid in 1,4-dioxane as a solvent revealed a notable absence of products of substitution. However, when the reaction was performed in refluxing toluene for 7 h, it resulted in a mixture of compounds. This mixture primarily comprised the starting 1-(2′-bromobenzoyl)-6-iodo-β-carboline (**9**), along with mono-substituted (**10**) and di-substituted (**11**) products, aligning with data previously reported [21]. After the chromatographic separation of the obtained mixture, targeted 1-(2′-bromobenzoyl)-6-phenyl-β-carboline (**10**) was isolated in 85% yield and converted into 9-phenylfascaplysin (**7**) according to the method derived from [25].

**Scheme 2:**
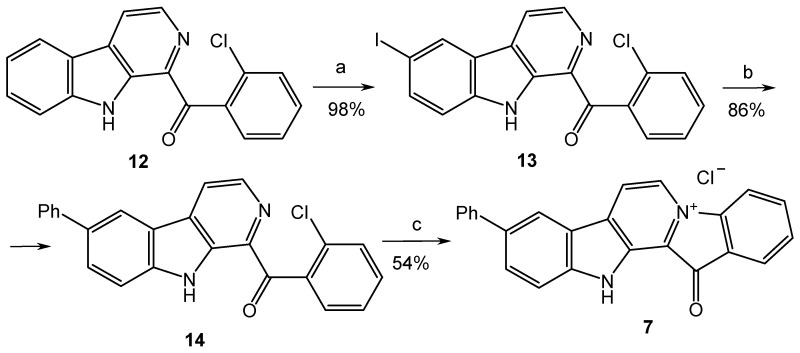
Reagents and conditions: (a) I_2_ (1.1 equiv.), TsOH (10 equiv.), DMSO, 110 °C, and 1 h; (b) PhB(OH)_2_ (1.2 equiv.), toluene, 2M aq. Na_2_CO_3_, Pd[P(C_6_H_5_)_3_]_4_ (0.025 equiv.), argon, 90 °C, and 7 h; (c) SiO_2_, 230 °C, 40 min, and HCl (aq).

Since the cross-coupling of 1-(2′-bromobenzoyl)-6-iodo-β-carboline (**9**) led to the mixture of products, we replaced the bromine atom in the benzoyl fragment of compound **8** with chlorine, since the chloroarenes are not coupled in convenient conditions for the Suzuki–Miyaura reaction (Scheme 2). This, in turn, led to the partial decomposition of the reaction mixture on the final cyclization step, which resulted in a decrease in the yield of target product ***7*** to 20–30%. Considerable efforts to optimize this stage allowed us to achieve a satisfactory (54%) yield of **7** by using silica gel as a support for its implementation.

**Scheme 3:**
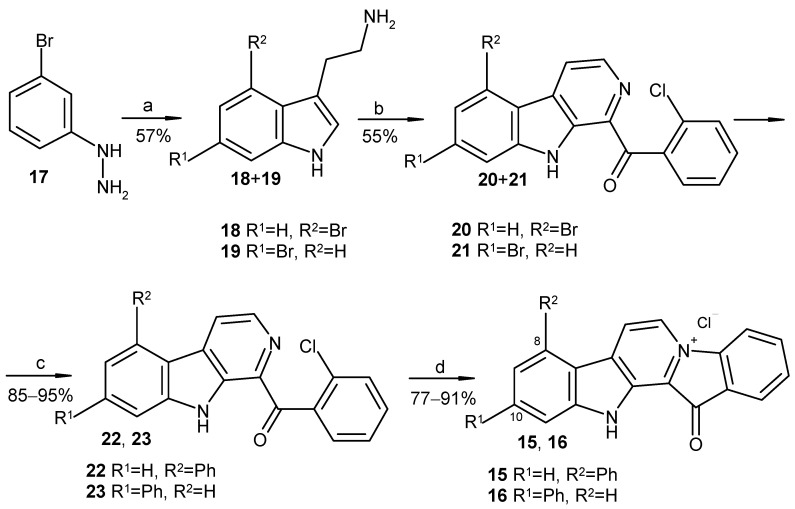
Reagents and conditions: (a) 4-bromobutanal, EtOH, H_2_O, autoclave, 150 °C, and 1 h; (b) 2′-chloroacetophenone (1 equiv.), I_2_ (0.8 equiv.), DMSO, 110 °C, 1 h, tryptamines **18** and **19** (1.0 equiv.), DMSO, 110 °C, and 4 h, then MPLC separation; (c) PhB(OH)_2_ (1.2 equiv.), toluene, 2M aq. Na_2_CO_3_, Pd[PPh_3_]_4_ (0.025 equiv.), argon, 90 °C, and 7 h; (d) SiO_2_, 220 °C, 40 min, and HCl (aq).

To elucidate the impact of the phenyl substituent position on the biological activity of fascaplysin analogs, we synthesized two isomers of compound **7** with the phenyl substituent at either C-8 (compound **15**) or C-10 (compound **16**). Starting tryptamines were obtained via the Fisher reaction between 3-bromophenylhydrazine (**17**) and 4-bromobutanal in an autoclave at 150 °C, which led to the mixture of isomeric bromotryptamines **18** and **19** (Scheme 3) [32]. Thereafter, the obtained mixture was used for the cascade coupling with *o*-chloroacetophenone using the method developed by Zhu et al. [30], which included the sequential iodination of the acetophenone, the Kornblum oxidation of the intermediate to corresponding phenylglyoxal, and the Pictet–Spengler condensation with the derivative of tryptamine, followed by oxidation to the derivative of β-carboline. After chromatography purification, two isomeric 5(7)-bromo-β-carbolines, **20** and **21**, were obtained with a total yield of 55%. Subsequently, the cross-coupling reaction of individual 5(7)-bromo-β-carbolines **20** and **21** with phenylboronic acid, following the previously established procedure used for the synthesis of compound **14**, resulted in the formation of 5(7)-phenyl-β-carbolines **22** and **23** (Scheme 3). Products **22** and **23** were subsequently transformed to 8-phenylfascaplysin (**15**) and its isomer **16** according to the procedure reported by the group of Radchenko [25].

**Scheme 4:**
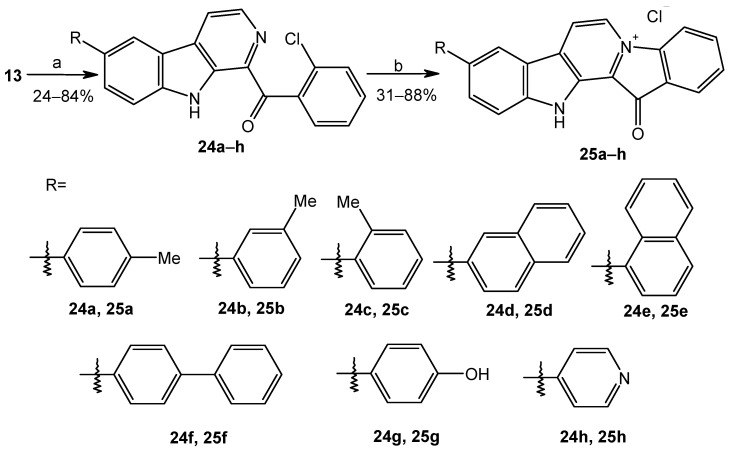
Reagents and conditions: (a) R-B(OH)_2_ (1.2 equiv.), toluene or THF, 2M aq. Na_2_CO_3_, Pd[PPh_3_]_4_ (0.025 equiv.), argon, 90 °C, and 7 h; (b) SiO_2_, 230 °C, 40 min, and HCl (aq).

To evaluate the additional potential for binding to therapeutic targets of compound **7**, a series of its derivatives were obtained containing small lipophilic fragments in the phenyl substituents (**25a [21]**, **25b**, **25c**), bulky substituents (**25d**–**25f**), and polar fragments capable of forming of hydrogen bonds (**25g**–**25h**) (Scheme 4). The successful synthesis of derivatives **25a**–**25h** was made possible through the utilization of silica gel as a solid-phase support, which facilitated the quaternization of β-carbolines **24a**–**24h**. Most of the target products could not be obtained using the conventional reaction conditions applied for the preparation of fascaplysin derivatives [33].

### 2.2. Antibacterial and Antiproliferative Activities of 9-Phenylfascaplysin and Its Isomers and Analogs In Vitro

**Figure 3:**
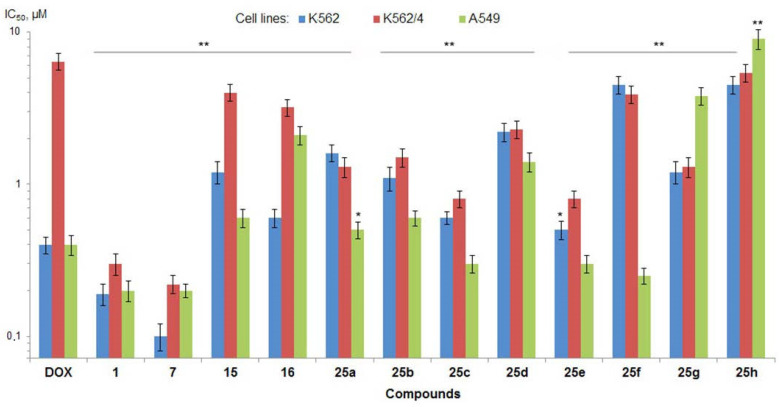
Antiproliferative activity (IC_50_, µM) of fascaplysin (**1**) and its derivatives in vitro (MTT-assays, 72 h exposure). Doxorubicin (DOX) was used as a reference drug (positive control); * *p* < 0.05 and ** *p* < 0.001 versus doxorubicin-treated cells.

The antibacterial activities of 9-phenylderivative (**7**) and paternal fascaplysin (**1**) were evaluated on a broad panel of pathogenic bacteria via the broth microdilution method. Gram-positive bacteria are presented by four *Staphylococcus aureus* strains (including two methicillin-resistant strains—MRSA), *Bacillus cereus*, a *Staphylococcus epidermidis* strain, four *Enterococcus* spp. strains (including two vancomycin-resistant strains—VRE), and *Mycobacterium smegmatis*. Gram-negative bacteria are presented by *Escherichia coli*. The screening panel includes ATCC reference stains of the main types of bacteria and antibiotic-resistant isolates, which refers to the priority pathogens list of the World Health Organization. For comparative study of antiproliferative properties of compounds, we used lung carcinoma cells A549 as an example of adherent-type cells and myelogenous leukemia cells K562 as an example of suspension tumor cells. Adherent or suspension tumor cell lines could have some differences in susceptibility for the tested compounds [34]. Additionally, we used K562/4 subline with expression of Pgp [35] for the assessment of the ability of fascaplysins to circumvent MDR associated with the expression of efflux proteins. Vancomycin (Van) and rifampicin (Rif) were used as positive controls for antibacterial screening, and doxorubicin (DOX) was used as a reference for evaluating the antitumor activity. The results of comparative tests of antibacterial potency and antiproliferative activity are shown in Table 1 and on Figure 3, respectively.

Screening results showed that paternal fascaplysin (**1**) has high antimicrobial activity against the majority of studied Gram-positive bacteria, ranging from 0.0075 µg/mL against the *S. epidermidis* to 1 µg/mL against the *E. faecium* (Table 1). However, both strains of *E. faecalis* and Gram-negative *E. coli* were not susceptible to **1** (MIC ≥ 8 µg/mL). In addition, fascaplysin demonstrated potent antiproliferative activity against two human tumor cell lines (leukemia cells K562 and carcinoma cells A549) close to reference Dox (Figure 3, Table S1). Moreover, in striking contrast to Dox, fascaplysin efficiently circumvented MDR of K562/4 cells, mediated by the overexpression of Pgp.

9-Phenylfascaplysin (**7**), as an unsubstituted fascaplysin, exhibited high antimicrobial activity, but the introduction of a phenyl group at C-9 led to changes in antibacterial properties. Thus, this modification led to a significant increase in activity against the majority of strains susceptible to fascaplysin **1** and *E. faecalis* resistant to this alkaloid (Table 1). At the same time, some decrease in activity of **7** was observed for *E. faecium* and *M. smegmatis*. No change in antimicrobial activity compared to fascaplysin was observed for Gram-negative *E. coli*. Derivative **7** also has high potential as an antitumor agent: its antiproliferative activity was close to fascaplysin (**1**), with a slightly increasing potency against wild-type K562 cells (Figure 3, Table S1).

**Figure 4:**
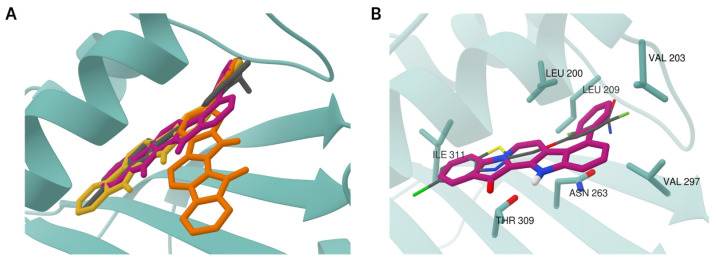
Structure of *S. aureus* FtsZ (PDB ID 4DXD) in complex with compounds **15**, **7**, and **16** predicted by molecular docking. (**A**) Superposition of 9PC (gray) and best-scoring poses of compounds **15** (pink), **7** (yellow), and **16** (orange) at the binding site. (**B**) Binding site residues interacting with compound **15** (pink) were identified via PLIP v. 2.3.0. Moreover, 9PC is shown as thin gray sticks colored by heteroatoms.

Following on from the data presented in Table 1, phenylfascaplysins **15** and **16** exhibited antibacterial activity but were less potent than their isomer at C-9 (**7**). The antiproliferative activities of both compounds **15** and **16** were also significantly lower than those of paternal fascaplysin (**1**) and its 9-phenyl derivative **7** (Figure 3, Table S1). The data we have acquired indicate that the remarkable antibacterial potency of compound **7**, when compared to its regioisomers that differ in the position of the phenyl substituent within the fascaplysin core structure, arises from distinctive interactions with its intracellular targets (Table 1). The data obtained, combined with the fact that the antimicrobial activity of compound **7** is comparable to the antimicrobial activity of compounds **6a**–**6d** (exceptionally high activity against Gram-positive MRSA but low activity against Gram-negative *E. coli*), allow us to form a hypothesis about the unity of their molecular targets [15]. To assess whether the therapeutic target of 9-phenylfascaplysin is FtsZ, we conducted molecular docking experiments involving compounds **15**, **7**, and **16** with *S. aureus* FtsZ (PDB ID 4DXD), as described in Methods. The binding modes of the top-ranked poses of compounds **15** and **7** were similar to that of the FtsZ inhibitor PC190723 from the PDB crystal structure (Figure 4A). In contrast, the orientation of compound **16** was orthogonal to the position of other ligands with the fascaplysin moiety oriented outside the protein. The phenyl substituent of all fascaplysin derivatives occupied the portion of the cleft in proximity to the loop interacting with its hydrophobic residues. Some of the interacting residues reported for PC190723 [36] were identified for compound **15**, for which, according to calculations, the greatest affinity for the therapeutic target under the study was expected (Figure 4B). Although we conducted docking experiments with the presumed molecular target of substituted at C-9 fascaplysins, we were unable to establish a meaningful correlation between the computed data and the MIC values recorded for the investigated compounds. While these findings do not rule out the possibility of an interaction with the mentioned FtsZ, they strongly suggest the existence of additional therapeutic targets for the studied compounds within the bacterial cell.

Some of the synthesized analogs of fascaplysin, specifically derivatives **25b**, **25e**, and **25f**, exhibit antibacterial efficacy similar to lead compound **7**. Notably, they display enhanced activity against *E. faecium* strains. Furthermore, compounds **25d** and **25f** demonstrate substantially reduced metabolic activity of tumor cells, suggesting the potential for an improved therapeutic index. However, it is important to note that a majority of these derivatives exhibit lower solubility in water compared to lead compound **7**. An attempt to enhance the aqueous solubility of **25h** by substituting the phenyl group in compound **7** with a pyridine residue resulted in improved solubility. Nevertheless, this modification led to a significant decrease in antimicrobial activity, which is notably lower than that of the original compound **7**. Accordingly, due to its outstanding activity, 9-phenylfascaplysin (**7**) was selected for the further evaluation of antibacterial and anticancer efficacy and acute toxicity in vivo.

### 2.3. Study of the Therapeutic Potential of 9-Phenylfascaplysin In Vivo

#### 2.3.1. Study of the Antibacterial Efficacy of 9-Phenylfascaplysin In Vivo

**Figure 5:**
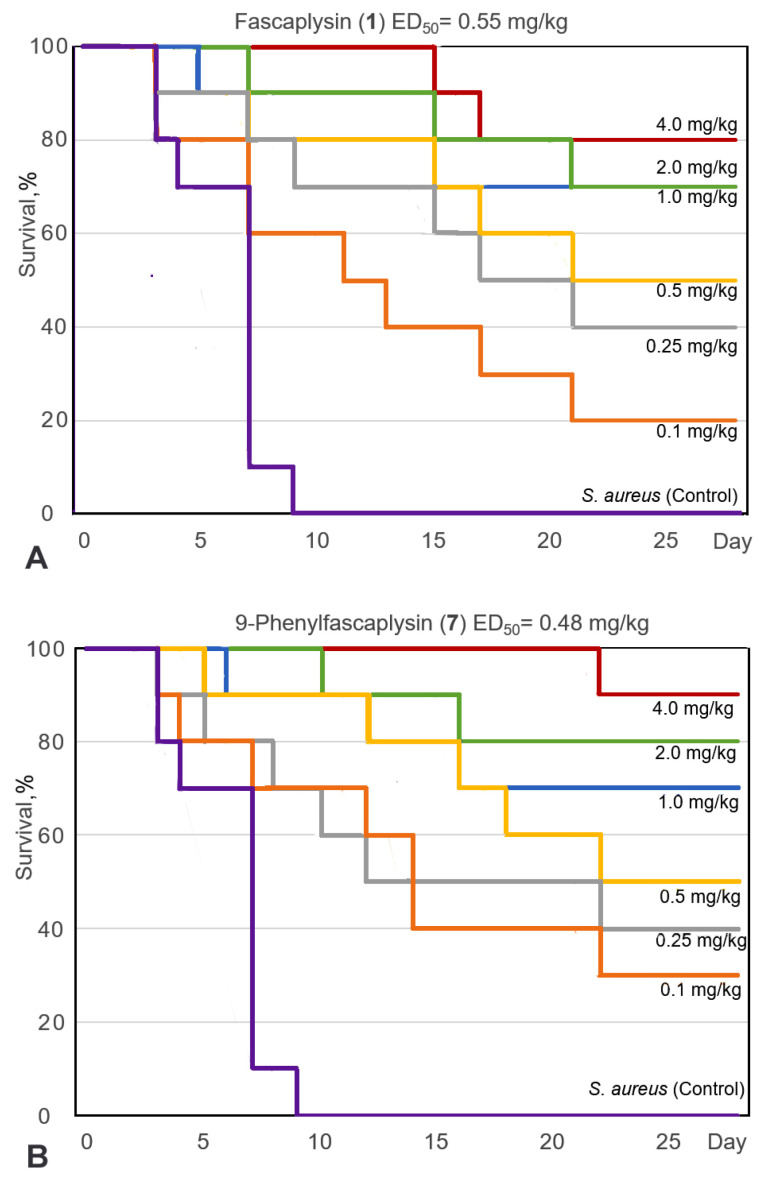

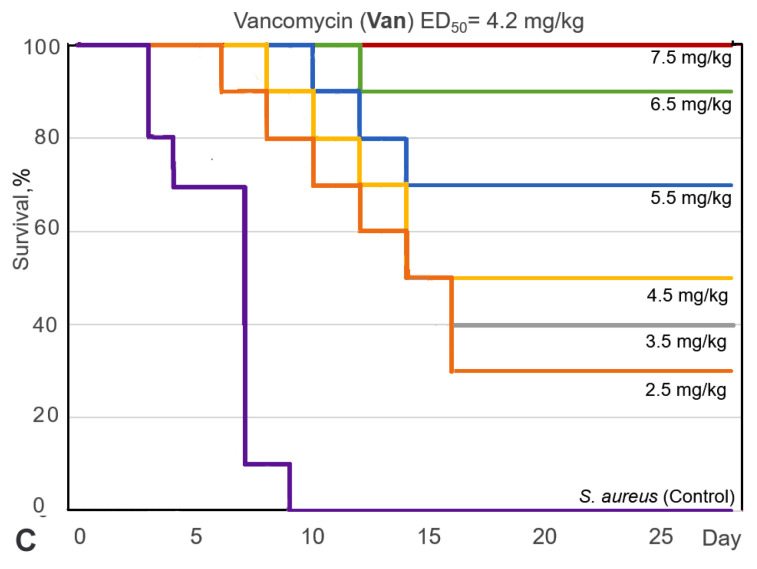
Survival curves (Kaplan–Meier) of mice infected with *S. aureus* after intravenous (i.v.) administration of fascaplysin (**A**), 9-phenylfascaplysin (**B**), and vancomycin (**C**). Mice (*n* = 10 for each group) were treated 1 h after infection via corresponding single doses of the drugs shown on the curves.

For the selected 9-phenylfascaplysin (**7**), an estimation of the antibacterial efficacy in comparison to fascaplysin (**1**) and reference vancomycin (Van) was carried out using a mouse staphylococcal sepsis model for single-dose intravenous (i.v.) administration. The study’s results are shown on Figure 5 and in Table S2. Compound **7** was less water soluble than fascaplysin (**1**), which complicated its in vivo testing. However, the addition of 10–20% ethanol or PEG600 as solubilizing agents significantly improved the aqueous solubility of compound **7**. In this study, the death of animals in the treatment groups was noted up to the 22nd day (Figure 5A,B), while the observation for animals was carried out for 28 days, after which all mice were euthanized. It is in contrast to the study [15], in which the animals were observed after treatment with fascaplysin for only for 7 days.

The results of this study showed that the survival rate of the mice, infected with *S. aureus*, increased after single-dose i.v. administration of compound **7**, Van, or paternal fascaplysin **1** in a dose-dependent manner (Figure 5). Based on data of the survival of the animal, the effective dose (ED_50_) values of the tested drugs were calculated (Figure 5, Table S2). The ED_50_ value of 9-phenylfascaplysin (**7**) was almost nine times lower than that for the reference Van. At the same time, the ED_50_ value for compound **7** was unexpectedly similar to **1**, which is in contrast to the results obtained for *S. aureus* in vitro, where derivative **7** was about two orders more active than unsubstituted fascaplysin (**1**). In both cases, 100% survival of experimental animals was not achieved for doses of **1** or **7**. These results can be ascribed to two plausible factors. Firstly, the higher lipophilicity of compound **7** (LogP = 2.0), in contrast to fascaplysin (LogP = 0.28, lipophilicity predicted using ClogP software v.2.2 at www.molinspiration.com, accessed on 31 January 2022), may facilitate its binding with albumins or lead to a more pronounced distribution within the lipophilic organs and tissues of the test subjects. Secondly, it might undergo more rapid metabolism, resulting in a decrease in the concentration of the active compound in the bloodstream, ultimately leading to a reduction in the therapeutic effect. Remarkable, the structure of compound **7** does not contain sp^3^-hybridized carbon atoms. According to the recently developed drug-likeness parameter Fsp^3^, this indicates a low likelihood of using this compound as a drug and indicates the need to increase this parameter during the subsequent modification of the identified lead compound [37].

#### 2.3.2. Evaluation of the Antitumor Activity of 9-Phenylfascaplysin (**7**) In Vivo

Previously, we were the first to evaluate the antitumor effect of fascaplysin against the ascitic form of Ehrlich carcinoma in vivo [10]. In the same study, we also showed that fascaplysin primarily has an antiproliferative effect on actively dividing cells. Over the past three decades, significant progress has been made in understanding the mechanisms of action of fascaplysin on various types of cells. Apparently, the first effect of fascaplysin is the ability to selectively inhibit CDK 4, which leads to cell cycle arrest and causes its cytostatic effect [38]. At the same time, this effect does not explain the full breadth of the therapeutic activity of this alkaloid, since it is able to suppress viability in Rb-null cancer cells [39]. Upon increasing the time of exposure to cell culture, a complex cascade of interactions is realized, the end result of which is their apoptotic death, which is accompanied by characteristic manifestations (formation of apoptotic bodies, activation of caspases-3 and -9, PARP-1 cleavage, etc.) [40]. The early event involves mitochondrial damage to the cells, resulting in the perturbation of mitochondrial membrane potential (Δψm). It (i) leads to the opening of mitochondrial permeability transition pores (PTP), which release proapoptoic proteins from mitochondria to cytosol; (ii) decreases in ATP levels and an increase in the content of reactive oxygen species (ROS); and (iii) triggers autophagy in response [41]. ROS, in turn, lead to DNA damage and the launch of repair systems. Fascaplysin is capable of directly intercalating into DNA [42]. It also causes the inhibition of the PI3K/AKT/mTOR signaling cascade, at least partly, through the inhibition of VEGFR3, VEGFR2, and TRKA [39,43]. The inhibition of this critical signaling cascade is responsible for the antiangiogenic properties of fascaplysin and partially determines its apoptotic effect via the inhibition of the biosynthesis of survivin. These mechanisms make it possible to characterize fascaplysin as a promising lead compound for the development of a multitargeted antitumor drug with increased resistance potential.

Therefore, to evaluate the antitumor activity of compound **7** in comparison to fascaplysin in vivo, we used ascitic and solid forms of the mouse model of Ehrlich carcinoma. The tested substances were administered to animals intraperitoneally (i.p.) in a volume of 0.5 mL daily five times; starting from the next day after tumor inoculation, compound **7** was administered in a 20% aqueous ethanol solution. Dox was used as a positive control and along with compound **7** for combinational treatment. For the evaluation of the antitumor activity of 9-phenylfascaplysin (**7**) on a murine ascitic, the Ehrlich adenocarcinoma daily dose of test substances was used as follows: Dox monotherapy—0.25 mg/kg (reference drug—positive control); monotherapy of compound **7** at three doses—5 mg/kg, 2.5 mg/kg and 1.25 mg/kg; and combined treatment of **7** with Dox—1.25 mg/kg and 0.25 mg/kg, respectively. The obtained results are given in Table 2.

This study’s results revealed a high antitumor potency of compound **7** in a dose of 5 mg/kg. Survival in this group on day 62 after tumor inoculation (experiment termination time) was 80%, and the increase in life span was 262.5%. This antitumor effect was close to the reference drug DOX at a dose of 0.25 mg/kg, which led to 100% survival and an increase in lifespan of 284.8%. The antitumor effect of **7** has a positive dose-response relationship. Thus, at a lower dose (2.5 mg/kg) of 9-phenylfascaplysin (**7**), survival in this group was 30% and ILS was 98%. At the same time, **7**, at a dose of 1.25 mg/kg, did not have a significant effect on increasing the lifespan or survival of animals. The combined effect of compound **7** at a dose of 1.25 mg/kg with the reference DOX at a dose of 0.25 mg/kg did not have a synergistic effect. Therefore, the combined usage of 9-phenylfascaplysin (**7**) and DOX reduces the therapeutic effect of the latter, which may be due to their mutual competitive effect on tumor cells due to the presence of common targets. Compared to fascaplysin, which, at doses ranging from 5 mg/kg to 20 mg/kg, did not reliably increase MST for this experimental model [10], the obtained results indicate significant progress in the optimization of the fascaplysin structure toward the creation of effective antitumor agents. Determining what changes in the molecular mechanisms of action occurred after the introduction of the phenyl substituent at C-9 of fascaplysin requires comprehensive study and will be the subject of a separate publication.

**Figure 6:**
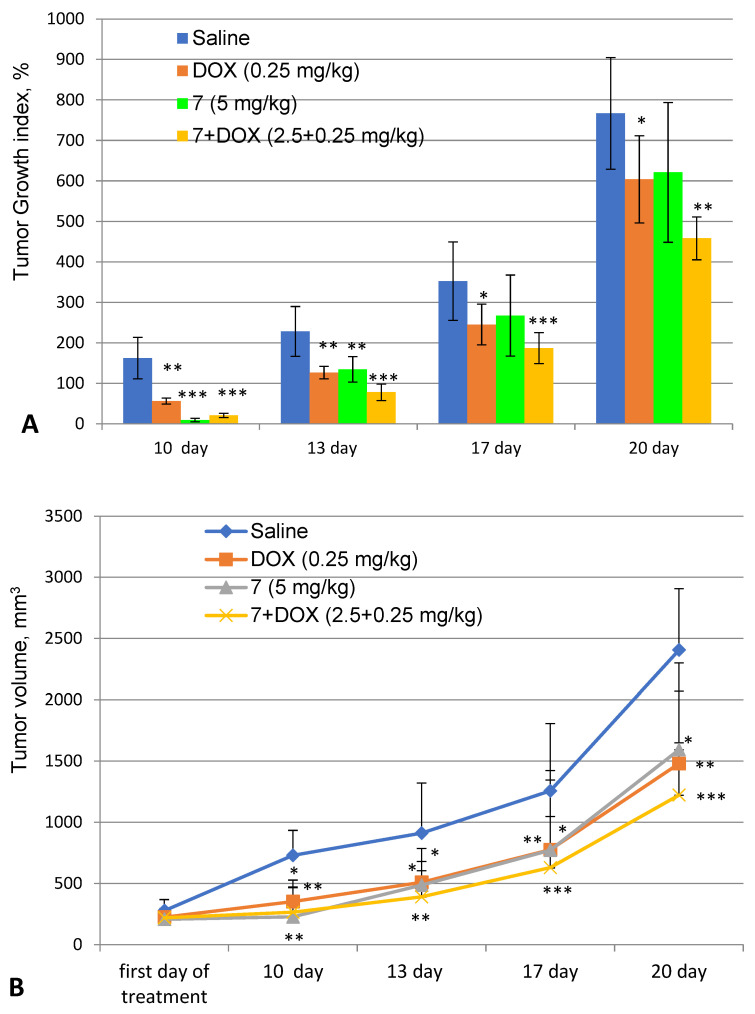
Dynamics of tumor growth. Tumor growth index (**A**) and change in tumor volume (**B**). Saline—control group of animals (untreated); DOX—group of animals treated with doxorubicin at a dose of 0.25 mg/kg; **7**—9-phenylfascaplysin (**7**) at a dose of 5 mg/kg; and 7 + DOX—9-phenylfascaplysin (**7**) at a dose of 2.5 mg/kg in combination with DOX at a dose of 0.25 mg/kg. Results are presented as mean ± SEM (*n* = 10). The significance of the differences was estimated via one-way ANOVA, followed by Tukey’s test versus the saline group. Significant differences are presented as * *p* < 0.05, ** *p* < 0.01, and *** *p* < 0.001.

9-Phenylfascaplysin (**7**), during the course of treatment, significantly inhibited the growth rate of Ehrlich adenocarcinoma inoculated subcutaneously. Thus, in the treatment group, the tumor volume on the last day of treatment was in three times less than that in the control (-) group, and the TGI value was only 9% (Figure 6).

However, after finishing the treatment course, a marked increase in tumor growth was observed in this group. Starting from the 13th day of the experiment, compound **7** had an antitumor effect comparable to DOX: it contributed to decreases in the growth rate and the tumor mass of approximately 1.5 times and inhibited tumor growth by 30%. The final data correlate with known antitumor effects of fascaplysin and some of its derivatives against solid tumors (A375, 1 mg/kg, reduction in tumor volume by three times [39]; HCT-116, 4 mg/kg, and TGI 42% [44]; S180, 5 mg/kg, and TGI 32.6% [45]; the Ehrlich solid tumor model, 4 mg/kg, and TGI 30% [46]). Our observations further substantiate the previously mentioned hypothesis regarding the swift metabolism or clearance of compound **7** from the experimental animals, ultimately resulting in the cessation of the antitumor efficacy. When administered in combination with DOX, compound **7** demonstrated a modest enhancement in the therapeutic antitumor effect, as indicated in Table 3.

Additionally Table 4 shows that 6 days after tumor inoculation, the body weight of mice was almost unchanged. On days 13 and 20, in the saline group, body weight decreased by 6 and 15%, respectively, which is connected to cancer cachexia. In the groups DOX, **7**, and **7** + DOX, the weights of the animals were more stable, which is probably due to the suppression of tumor growth, although there were no significant differences comparable to the saline DOX groups.

### 2.4. Acute Toxicity of 9-Phenylfascaplysin (***7***)

The study of acute toxicity of compound **7** was carried out on white outbred female mice at doses of 10 to 40 mg/kg (single injection) intraperitoneally. Also, in a volume of 0.2 mL, an aqueous solution of 20% ethanol was injected intraperitoneally (solvent control). Lethality was assessed when observing animals for 14 days and expressed as a percentage (%) in relation to the total number of animals in the group. The research results are shown in Table 5.

The LD_10_ and LD_50_ of 9-phenylfascaplysin (**7**) were calculated to be 12 mg/kg and 25 mg/kg (confidence intervals were 21.5–28.0 mg/kg), respectively. Obtained data make it possible to classify this compound as moderately toxic, according to [47]. As previously shown, the LD_50_ of unsubstituted fascaplysin, when administered intravenously in mice, is 38 mg/kg [45].

## 3. Materials and Methods

### 3.1. Chemistry

All starting materials were commercially available. Commercial reagents were used without any purification. The products were isolated via MPLC: Buchi B-688 pump, glass column C-690 (15 × 460 mm), or PP-cartridge (150 × 12 mm) with Silica gel (particle size 0.015–0.040 mm), as well as a Knauer K-2001 UV-detector. The analytical examples were purified using a Shimadzu HPLC system (model: LC-20AP) equipped with a UV detector (model: SPD-20A) using a Supelco C18 (5 µm, 20 × 250 mm) column and a MeOH:water (20:80, 50:50, 70:30) mobile phase via isocratic elution at a flow rate of 15 mL/min. NMR spectra were recorded with a NMR instrument operating at 400 MHz (^1^H) and 100 MHz (^13^C). ^1^H NMR spectra were referenced with TMS as the internal standard, in some cases, to the residual signal of used solvents. Chemical shifts in ^13^C NMR spectra were determined relative to the ^13^C signal of the TMS or used solvents. Chemical shifts were given on the *δ* scale (ppm). Coupling constants (J) were given in Hz. Multiplicities were indicated as follows: s (singlet), d (doublet), t (triplet), q (quartet), m (multiplet), or br (broadened). The original spectra of the relative compounds can be found in the Supplementary Materials. High-resolution mass spectra (HRMS) were obtained using a time-of-flight (TOF) mass spectrometer (model Agilent TOF 6210) equipped with an electrospray source at atmospheric pressure ionization (ESI).

#### 3.1.1. Preparation of Compounds **9**, **13**

Corresponding β-carboline (0.98 mmol), TsOH (1.180 g, 6.9 mmol), and iodine (2.680 g, 10.5 mmol) were added to 15 mL of DMSO, and the resulting solution was heated at 150 °C for 1 h. Then, the reaction mixture was cooled to room temperature, followed by the addition of water (200 mL) and extraction with CHCl_3_ (5 × 20 mL). The extract was washed with 10% Na_2_S_2_O_3_, dried over Na_2_SO_4_, filtered, and evaporated under reduced pressure. The residue was triturated with Et_2_O and dried to give the target product.

For 1-(2′-bromobenzoyl)-6-iodo-β-carboline (**9**): yellow solid, 70%. ^1^H NMR (C_6_D_6_): *δ* 8.28 (d, 1H), 8.12 (d, 1H), 7.51 (dd, 1H), 7.40 (dd, 1H), 7.37 (d, 1H) 7.24 (d, 1H), 6.96 (dd, 1H), 6.76 (ddd, 1H), and 6.33 (d, 1H). ^13^C NMR (C_6_D_6_): *δ* 197.7, 170.3, 140.7, 139.6, 138.5, 136.8, 135.8, 135.5, 132.4, 130.3, 130.1, 129.3, 126.1, 122.7, 119.9, 119.5, 118.5, and 113.6. HRMS-ESI, *m*/*z*: [M + H]^+^ calculated for C_18_H_11_^79^BrIN_2_O^+^ 476.9099, found 476.9123.

For 1-(2′-chlorobenzoyl)-6-iodo-β-carboline (**13**): yellow solid, 98%. ^1^H NMR (CDCl_3_): *δ* 10.45 (br. s, 1H), 8.58 (d, *J* = 4.89 Hz, 1H), 8.51 (s, 1H), 8.11 (d, *J* = 4.9 Hz, 1H), 7.89 (dd, *J* = 8.6, 1.6 Hz, 1H), 7.59 (dd, *J* = 7.4, 1.5 Hz, 1H), and 7.39–7.54 (m, 4H). ^13^C NMR (CDCl_3_): *δ* 197.5, 140.0, 139.2, 138.0, 137.7, 136.5, 135.8, 131.8, 131.3, 130.8, 130.4, 130.1, 129.9, 126.4, 123.2, 119.2, 116.0, and 114.0. HRMS-ESI, *m*/*z*: [M + H]^+^ calculated for C_18_H_11_^35^ClIN_2_O^+^ 432.9599, found 432.9608.

#### 3.1.2. Preparation of 1-(2′-Bromobenzoyl)-6-phenyl-β-carboline (**10**)

1-(2′-Bromobenzoyl)-6-iodo-β-carboline (**9**) (15 mg, 0.032 mmol), phenylboronic acid (2 mg, 0.016 mmol), tetrakis(triphenylphosphine)palladium (0) (0.9 mg, 2.5%), 300 µL toluene, and 120 µL 2M aqueous solution of Na_2_CO_3_ were placed in a 5 mL ground test tube. The reaction mixture was purged with argon and heated at 90 °C for 7 h. The cooled solution was diluted with 50 mL of water and extracted with EtOAc (3 × 20 mL). The extract was evaporated under reduced pressure. The residue was chromatographed via MPLC using benzene as an eluent. The yield of the target product was 85%.

^1^H NMR (400 MHz, CDCl_3_): *δ* 10.48 (br. s, 1H), 8.60 (d, 1H), 8.40 (s, 1H), 8.23 (d, 1H), 7.92 (dd, 1H), 7.73 (dd, 1H), 7.60 (dd, 1H), 7.52–7.56 (m, 1H), 7.48–7.51 (m, 1H), 7.37–7.46 (m, 1H). ^13^C NMR (100 MHz, CDCl_3_): *δ* 199.8, 142.8, 142.1, 141.9, 140.5, 138.9, 136.9, and 136.3, 134.7, 133.5, 132.9, 131.4, 131.1, 130.7, 130.5, 128.9, 128.6, and 128.5. HRMS-ESI, *m*/*z*: [M + H]^+^ calculated for C_24_H_16_^81^BrN_2_O^+^ 429.0441, obtained 429.0443.

#### 3.1.3. Preparation of 9-Phenylfascaplysin (**7**) from Compound **10**

1-(2′-Bromobenzoyl)-6-phenyl-β-carboline (0.036 mg, 0.08 mmol) was heated in a closed vial at 220 °C for 40 min. After cooling, the mixture was washed with EtOAc (3 × 3 mL) and water (3 × 10 mL). The aqueous layers were combined and acidified with hydrochloric acid. Then, the sample was evaporated under reduced pressure. The resulting product was a red powder (0.027 mg, 77%).

^1^H NMR (400 MHz, MeOH-d4): *δ* 9.33 (s, 1H), 8.97 (s, 1H), 8.59 (s, 1H), 7.68–7.77 (m, 4H), 7.62 (dd, *J*1 = *J*2 = 7.7 Hz, 1H), 7.49–7.64 (m, 2H), and 6.49 (d, *J* = 8.4 Hz, 1H). ^13^C NMR (100 MHz, MeOH-d4): *δ* 181.6, 147.2, 146.6, 141.4, 139.3, 136.9, 136.5, 133.6, 131.3, 128.7, 126.5, 124.0, 121.3, 120.3, 118.0, 115.1, and 113.5. HRMS-ESI, *m*/*z*: [M]^+^ calculated for C_24_H_15_N_2_O^+^ 347.1179, obtained 347.1175.

#### 3.1.4. Preparation of Mixture of Tryptamines **18** and **19**

A mixture of 4-bromobutanal (1.33 g, 8.8 mmol), 3-bromophenylhydrazine hydrochloride (0.50 g, 2.2 mmol), EtOH (3 mL), and H_2_O (1 mL) was placed into an autoclave and heated at 150 °C for 1 h. After cooling, the mixture was poured into H_2_O (100 mL) and extracted with EtOAc (3 × 50 mL). Then, the aqueous solution was treated with NaOH to pH 12 and extracted with CH_2_Cl_2_ (3 × 50 mL). The combined organic layer was washed with brine (2 × 100 mL), dried over Na_2_SO_4_, and evaporated. After flash column chromatography (EtOAc, then EtOH/NH_3_), compounds **18** and **19** were isolated as a mixture at a ratio of 1:1 (brown oil, 300 mg, 57%).

#### 3.1.5. Preparation of Substituted 1-Benzoyl-β-carbolines **20**, **21**

2-Chloroacetophenone (0.458 mmol) and iodine (92 mg, 0.366 mmol) were added to 2 mL of DMSO, and the resulting solution was heated at 90 °C for 1 h. After that, the mixture of compounds **18** and **19** (0.458 mmol) was added to the solution, and this solution was stirred at the same temperature for 3–4 h until the completion of the reaction (monitored by TLC). Then, the reaction mixture was cooled to room temperature, followed by the addition of water (50 mL) and extraction with EtOAc (3 × 25 mL). The extract was washed with 10% Na_2_S_2_O_3_, dried over Na_2_SO_4_, and filtered and evaporated under reduced pressure. The residue was purified via MPLC using benzene and benzene/hexanes as the eluent to give the desired product.

For 5-bromo-1-(2-chlorobenzoyl)-β-carboline (**20**): yellow solid, 27%. ^1^H NMR (400 MHz, CDCl_3_): *δ* 10.60 (br. s, 1H), 8.79 (d, *J* = 5.0 Hz, 1H), 8.63 (d, *J* = 5.0 Hz, 1H), 7.62 (d, *J* = 0.6 Hz, 1H), 7.59–7.61 (m, 1H), and 7.41–7.57 (m, 5H). ^13^C NMR (100 MHz, CDCl_3_): *δ* 197.6, 142.1, 139.1, 138.1, 136.7, 135.5, 131.8, 131.3, 131.3, 130.0, 129.9, 129.9, 126.3, 125.0, 121.0, 120.1, 118.2, and 111.0. HRMS-ESI, *m*/*z*: [M + H]^+^ calculated for C_18_H_11_^79^Br^35^ClN_2_O^+^384.9738, obtained 384.9745.

For 7-bromo-1-(2-chlorobenzoyl)-β-carboline (**21**): yellow solid, 28%. ^1^H NMR (400 MHz, CDCl_3_): *δ* 10.44 (br. s, 1H), 8.58 (d, *J* = 4.9 Hz, 1 H), 8.14 (d, *J* = 4.9 Hz, 1H), 8.05 (d, *J* = 8.3 Hz, 1H), 7.81 (d, *J* = 1.4 Hz, 1 H), 7.60 (dd, *J* = 7.4, 1.6 Hz, 1H), and 7.39–7.56 (m, 4H). ^13^C NMR (100 MHz, CDCl_3_): *δ* 197.5, 141.8, 139.3, 138.1, 136.8, 135.8, 131.8, 131.3, 131.2, 130.1, 129.9, 126.3, 124.5, 123.2, 123.0, 119.7, 119.0, and 115.2. HRMS-ESI, *m*/*z*: [M + H]^+^ calculated for C_18_H_11_^79^Br^35^ClN_2_O^+^384.9738, obtained 384.9743.

#### 3.1.6. Preparation of Substituted 1-Benzoyl-β-carbolines **14**, **22**, **23**, **24a**–**24h**

Method 1 (for compounds **14**, **22**, **23**, **24a**–**24f**): 6-iodo-1-(2-chlorobenzoyl)-β-carboline (**13**) (23.8 mg, 0.055 mmol), the corresponding phenylboronic acid (0.08 mmol), Na_2_CO_3_ (8.5 mg, 0.08 mmol), tetrakis(triphenylphosphine)palladium (0) (0.9 mg, 1.4 × 10^−3^ mmol), H_2_O (150 µL), and toluene (300 µL) were mixed in a 1.5 mL vial with a tight-fitting cap and purged with argon, and the resulting mixture was stirred at 90 °C for 7 h. After cooling, the reaction mixture was diluted with water and extracted with EtOAc (3 × 10 mL). The extract was evaporated under reduced pressure. The residue was chromatographed via MPLC, using benzene, toluene, or their mixture with hexane as an eluent.

Method 2 (for compounds **24g**–**24h**): 6-iodo-1-(2-chlorobenzoyl)-β-carboline (**13**) (100 mg, 0.23 mmol), the corresponding phenylboronic acid (0.67 mmol), Na_2_CO_3_ (36 mg, 0.34 mmol), tetrakis(triphenylphosphine)palladium (0) (26 mg, 40 × 10^−3^ mmol), H_2_O (0.5 mL), and THF (2.5 mL) were added to a 5 mL screw cap test tube and purged with argon, and the resulting mixture was stirred at 90 °C for 6 h. After cooling, the reaction mixture was diluted with water and extracted with EtOAc (3 × 20 mL). The extract was evaporated under reduced pressure. The residue was chromatographed via MPLC using EtOAc, toluene, hexane, and their mixtures as the eluents.

For compound **14**: yellow solid, 86%. ^1^H NMR (400 MHz, CDCl_3_): *δ* 10.47 (br. s, 1H), 8.58 (d, *J* = 5.0 Hz, 1H), 8.38 (s, 1H), 8.21 (d, *J* = 5.0 Hz, 1H) 7.89 (dd, *J* = 8.5, 1.6 Hz, 1H), 7.67–7.75 (m, 3H) 7.62 (dd, *J* = 7.3, 1.8 Hz, 1H), and 7.36–7.55 (m, 6H). ^13^C NMR (100 MHz, CDCl_3_): *δ* 197.6, 141.2, 140.5, 138.9, 138.3, 135.6, 134.7, 137.2, 131.9, 131.8, 131.2, 130.0, 129.9, 129.1, 128.9, 127.3, 127.0, 126.3, 121.3, 120.3, 119.2, and 112.3. HRMS-ESI, *m*/*z*: [M + H]^+^ calculated for C_24_H_16_^35^ClN_2_O^+^ 383.0946, obtained 383.0940.

For compound **22**: yellow solid, 85%. ^1^H NMR (400 MHz, CDCl_3_): *δ* 10.61 (br. s, 1H), 8.33 (d, *J* = 5.0 Hz, 1H), 7.39–7.72 (m, 13H), and 7.26 (d, *J* = 1.3 Hz, 1H). ^13^C NMR (100 MHz, CDCl_3_): *δ* 197.7, 141.5, 140.1, 139.3, 138.7, 138.4, 137.1, 135.3, 131.8, 131.4, 131.2, 130.0, 129.8, 129.2, 128.9, 128.7, 128.1, 126.3, 122.4, 120.8, 118.5, and 110.9. HRMS-ESI, *m*/*z*: [M + H]^+^ calculated for C_24_H_16_^35^ClN_2_O^+^ 383.0946, obtained 383.0944.

For compound **23**: yellow solid, 95%. ^1^H NMR (400 MHz, CDCl_3_): *δ* 10.51 (br. s, 1H), 8.58 (d, *J* = 4.9 Hz, 1H), 8.24 (d, *J* = 8.1 Hz, 1H), 8.18 (d, *J* = 4.9 Hz, 1H), 7.82 (d, *J* = 0.6 Hz, 1H), 7.71–7.77 (m, 2H), 7.60–7.65 (m, 2H), and 7.40–7.57 (m, 6H). ^13^C NMR (100 MHz, CDCl_3_): *δ* 197.6, 143.0, 141.8, 141.1, 139.0, 138.3, 137.3, 135.4, 131.8, 131.6, 131.2, 130.0, 129.9, 129.0, 127.8, 127.6, 126.3, 122.2, 120.8, 119.8, 119.1, and 110.4. HRMS-ESI, *m*/*z*: [M + H]^+^ calculated for C_24_H_16_^35^ClN_2_O^+^ 383.0946, obtained 383.0948.

For compound **24a**: yellow solid, 24%. ^1^H NMR (400 MHz, CDCl_3_): *δ* 10.45 (br. s, 1H), 8.57 (d, *J* = 4.9 Hz, 1H), 8.35 (s, 1H), 8.20 (d, *J* = 4.9 Hz, 1H), 7.87 (dd, *J* = 8.5, 1.7 Hz, 1H), 7.67 (d, *J* = 8.5 Hz, 1H), 7.61 (d, *J* = 7.9 Hz, 3H), 7.53–7.41 (m, 3H), 7.31 (d, *J* = 7.9 Hz, 2H), and 2.44 (s, 3H). ^13^C NMR (100 MHz, CDCl_3_): *δ* 197.6, 140.4, 138.9, 138.3, 138.3, 137.2, 136.8, 135.6, 134.6, 132.0, 131.8, 131.2, 130.1, 129.9, 129.6, 129.0, 127.2, 126.4, 121.3, 120.0, 119.2, 112.2, and 21.1. HRMS-ESI, *m*/*z*: [M + H]^+^ calculated for C_25_H_18_^35^ClN_2_O^+^397.1102, obtained 397.1098.

For compound **24b**: yellow solid, 84%. ^1^H NMR (400 MHz, CDCl_3_): *δ* 10.46 (br. s, 1H), 8.59 (d, *J* = 4.9 Hz, 1H), 8.38 (s, 1H), 8.21 (d, *J* = 4.9 Hz, 1H), 7.89 (dd, *J* = 8.4, 1.7 Hz, 1H), 7.69 (d, *J* = 8.4 Hz,1H), 7.62 (dd, *J* = 7.3, 1.7 Hz, 1H), 7.38–7.56 (m, 7H), 7.22 (d, *J* = 7.4 Hz, 1H), and 2.49 (s, 3 H). ^13^C NMR (100 MHz, CDCl_3_): *δ* 197.5, 141.2, 140.5, 139.0, 138.5, 138.3, 137.3, 135.5, 134.8, 132.0, 131.9, 131.3, 130.1, 130.0, 129.2, 128.8, 128.1, 127.8, 126.4, 124.4, 121.3, 120.3, 119.3, 112.2, and 21.6. HRMS-ESI, *m*/*z*: [M + H]^+^ calculated for C_25_H_18_^35^ClN_2_O^+^ 397.1102, obtained 397.1095.

For compound **24c**: yellow solid, 91%. ^1^H NMR (400 MHz, CDCl_3_): *δ* 10.49 (br. s, 1H), 8.57 (d, *J* = 4.9 Hz, 1H), 8.17 (d, *J* = 4.9 Hz, 1H), 8.13 (s, 1H), 7.65–7.70 (m, 1H), 7.60–7.65 (m, 2H), 7.42–7.55 (m, 3H), 7.30–7.38 (m, 4H), and 2.34 (s, 3H). ^13^C NMR (100 MHz, CDCl_3_): *δ* 197.6, 141.7, 140.1, 138.9, 138.3, 137.1, 135.6, 135.6, 135.1, 131.9, 131.8, 131.2, 131.0, 130.4, 130.2, 130.0, 129.9, 127.3, 126.4, 125.9, 122.2, 120.7, 119.2, 111.6, and 20.6. HRMS-ESI, *m*/*z*: [M + H]^+^ calculated for C_25_H_18_^35^ClN_2_O^+^397.1102, obtained 397.1104.

For compound **24d**: yellow solid, 28%. ^1^H NMR (400 MHz, CDCl_3_): *δ* 10.49 (br. s, 1H), 8.59 (d, *J* = 4.8 Hz, 1H), 8.50 (s, 1H), 8.24 (d, *J* = 4.8 Hz, 1H), 8.15 (s, 1H), 8.03–7.86 (m, 5H), 7.73 (d, *J* = 8.4, 1H), 7.62 (d, *J* = 6.7, 1H), and 7.56–7.42 (m, 5H). ^13^C NMR (100 MHz, CDCl_3_): *δ* 197.6, 140.6, 139.0, 138.5, 138.3, 137.2, 135.7, 134.6, 133.8, 132.4, 132.0, 131.8, 131.3, 130.1, 129.9, 129.4, 128.6, 128.1, 127.7, 126.4, 126.4, 125.9, 125.8, 121.4, 120.6, 119.3, and 112.4. HRMS-ESI, *m*/*z*: [M + H]^+^ calculated for C_28_H_18_^35^ClN_2_O^+^ 433.1102, obtained 433.1109.

For compound **24e**: yellow solid, 37%. ^1^H NMR (400 MHz, CDCl_3_): *δ* 10.53 (br. s, 1H), 8.58 (d, *J* = 4.9 Hz, 1H), 8.30 (s, 1H), 8.16 (d, *J* = 4.9 Hz, 1H), 7.89–7.98 (m, 3H), 7.77–7.81 (m, 1H), 7.72–7.76 (m, 1H), 7.64 (dd, *J* = 7.3, 1.7 Hz, 1H), 7.59 (t, *J* = 7.5 Hz, 1H), 7.50–7.56 (m, 3H), and 7.43–7.49 (m, 3H). ^13^C NMR (100 MHz, CDCl_3_): *δ* 197.6, 140.5, 140.0, 139.0, 138.3, 137.2, 135.7, 133.9, 133.8, 132.0, 131.9, 131.8, 131.2, 130.1, 129.9, 128.4, 127.7, 127.4, 126.4, 126.2, 126.0, 125.8, 125.4, 123.2, 120.9, 119.2, and 111.8. HRMS-ESI, *m*/*z*: [M + H]^+^ calculated for C_28_H_18_^35^ClN_2_O^+^ 433.1102, obtained 433.1100.

For compound **24f**: yellow solid, 22%. ^1^H NMR (400 MHz, CDCl_3_): *δ* 10.47 (br. s, 1H), 8.59 (d, *J* = 5.0 Hz, 1H), 8.43 (s, 1H), 8.22 (d, *J* = 5 Hz, 1H), 7.94 (dd, *J* = 8.5, 1.7 Hz, 1H), 7.80 (m, 2H), 7.75 (s, 1H), 7.73 (d, *J* = 2.7 Hz, 1H), 7.70 (m, 1H), 7.68 (s, 1H), 7.62 (dd, *J* = 7.4, 1.7 Hz, 1H), and 7.53–7.37 (m, 7H). ^13^C NMR (100 MHz, CDCl_3_): *δ* 197.6, 140.7, 140.6, 140.1, 139.9, 139.0, 138.3, 137.2, 135.7, 134.2, 132.0, 131.9, 131.3, 130.1, 139.9, 129.5, 129.0, 128.9, 127.7, 127.4, 127.1, 126.4, 121.4, 120.2, 119.2, 115.6, and 112.4. HRMS-ESI, *m*/*z*: [M + H]^+^ calculated for C_30_H_20_^35^ClN _2_O^+^ 459.1259, obtained 459.1265.

For compound **24g**: yellow solid, 69%. ^1^H NMR (400 MHz, CDCl_3_): *δ* 10.44 (br. s, 1H), 8.57 (d, *J* = 5.0 Hz, 1H), 8.32 (s, 1H), 8.20 (d, *J* = 5 Hz, 1H), 7.85 (dd, *J* = 8.5, 1.7 Hz, 1H), 7.62–7.68 (m, 4H), 7.41–7.53 (m, 3H), and 7.05 (d, *J* = 7.6 Hz, 2H). ^13^C NMR (100 MHz, CDCl_3_): *δ* 197.6, 159.0, 140.2, 138.9, 138.3, 137.2, 135.6, 134.4, 133.8, 132.0, 131.9, 131.2, 130.0, 129.9, 128.9, 128.3, 126.3, 121.3, 119.8, 119.2, 114.3, and 112.2. HRMS-ESI, *m*/*z*: [M + H]^+^ calculated for C_24_H_16_^35^ClN_2_O_2_^+^ 399.0895, obtained 399.0891.

For compound **24h**: yellow solid, 39%. ^1^H NMR (400 MHz, CDCl_3_): *δ* 10.55 (br. s, 1H), 8.73 (d, *J* = 5.0 Hz, 2H), 8.62 (d, *J* = 5.0 Hz, 1H), 8.47 (d, *J* = 0.8 Hz, 1H), 8.24 (d, *J* = 5.0 Hz, 1H), 7.95 (dd, *J* = 8.5, 1.8 Hz, 1H), 7.76 (d, *J* = 8.5 Hz, 1H), 7.66 (d, *J* = 6.1 Hz, 2H), 7.62 (dd, *J* = 7.3, 1.8 Hz, 1H), and 7.42–7.56 (m, 3H). ^13^C NMR (100 MHz, CDCl_3_): *δ* 197.6, 150.4, 148.3, 141.5, 139.3, 138.1, 137.2, 135.9, 131.8, 131.7, 131.3, 130.1, 129.9, 129.5, 128.5, 126.4, 121.7, 121.5, 120.5, 119.2, 116.0, and 112.7. HRMS-ESI, *m*/*z*: [M + H]^+^ calculated for C_23_H_15_^35^ClN_3_O^+^384.0898, obtained 384.0893.

#### 3.1.7. Preparation of Substituted Fascaplysins **7**, **15**, **16**, and **25a**–**25h**

Corresponding β-carboline (0.3 mmol) was dissolved in CHCl_3_ (5 mL), and SiO_2_ (100–200 µM, 1.0 g) was added, after which the solvent was evaporated under reduced pressure. The residue was kept at a temperature of 220–230 °C under argon for 40 min, after which it was cooled to room temperature and transferred to a Schott filter. The silica gel was first washed with CHCl_3_ to remove unreacted starting material, followed by washing with EtOH containing HCl. The ethanol extract was evaporated under reduced pressure, after which the target product was purified via either MPLC using CHCl_3_/EtOH 3/1 as an eluent or HPLC using 70% MeOH as an eluent.

For compound **7**: red solid, 54%. The spectral data are presented at 3.1.3.

For compound **15**: red solid, 77%. ^1^H NMR (400 MHz, CD_3_OD): *δ* 9.10 (br. s, 1H), 8.25 (d, *J* = 6.5 Hz, 1H), 8.03 (d, *J* = 7.1 Hz, 1H), 7.88–7.99 (m, 3H), 7.81 (d, *J* = 8.3 Hz, 1H), 7.72 (t, *J* = 7.3 Hz, 1H), 7.64 (br. s, 5H), and 7.39 (d, *J* = 7.1 Hz, 1H). ^13^C NMR (100 MHz, CD_3_OD): *δ* 181.8, 147.9, 147.1, 141.1, 140.9, 138.6, 136.8, 134.3, 132.1, 131.3, 129.0, 128.8, 128.4, 125.7, 125.4, 124.2, 124.2, 120.1, 117.7, 116.0, 115.1, and 112.2. HRMS-ESI, *m*/*z*: [M]^+^ calculated for C_24_H_15_N_2_O^+^ 347.1179, obtained 347.1173.

For compound **16**: red solid, 91%. ^1^H NMR (400 MHz, CD_3_OD): *δ* 9.37 (br. s, 1H), 8.92 (br. s, 1H), 8.52 (d, *J* = 7.4 Hz, 1H), 8.33 (d, *J* = 7.7 Hz, 1H), 8.03 (d, *J* = 7.2 Hz, 1H), 7.92–8.01 (m, 2H), 7.80 (d, *J* = 7.4 Hz, 3H), 7.73 (t, *J* = 7.2 Hz, 1H), 7.53 (t, *J* = 7.3 Hz, 2H), and 7.41–7.48 (m, 1H). ^13^C NMR (100 MHz, CD_3_OD): *δ* 181.8, 148.1, 147.8, 147.3, 141.2, 139.6, 136.9, 131.2, 128.9, 128.6, 127.2, 125.4, 124.4, 124.1, 122.8, 122.0, 119.7, 118.9, 116.0, 116.0, 115.2, and 110.7. HRMS-ESI, *m*/*z*: [M]^+^ calculated for C_24_H_15_N_2_O^+^ 347.1179, obtained 347.1177.

For compound **25a**: brick solid, 43%. ^1^H NMR (400 MHz, MeOH-d4): *δ* 9.27 (s, 1H), 8.92 (s, 1H), 8.41 (s, 1H), 8.25 (d, *J* = 7.3 Hz, 1H), 7.99–7.92 (m, 3H), 7.73–7.67 (m, 2H), 7.48 (d, *J* = 7.2 Hz, 2H), 7.14 (d, *J* = 7.1 Hz, 2H), and 2.24 (s, 3H). ^13^C NMR (100 MHz, MeOH-d4): *δ* 183.1, 148.7, 147.9, 142.8, 139.1, 138.4, 137.3, 137.3, 134.6, 133.3, 132.8, 131.0. 127.7, 127.6, 126.7, 125.5, 123.4, 122.0, 121.8, 121.6, 117.6, 116.6, 115.0, and 21.3. HRMS-ESI, *m*/*z*: [M]^+^ calculated for C_25_H_17_N_2_O^+^ 361.1335, obtained 361.144.

For compound **25b**: red solid, 31%. ^1^H NMR (400 MHz, MeOH-d4): *δ* 9.37 (d, *J* = 6.2 Hz, 1H), 9.02 (d, *J* = 6.2 Hz, 1H), 8.69 (s, 1H), 8.33 (d, *J* = 8.0 Hz, 1H), 8.15 (dd, *J* = 8.6, 1.6 Hz, 1H), 8.04 (d, *J* = 7.4 Hz, 1H), 7.97 (t, *J* = 7.4 Hz, 1H), 7.85 (d, *J* = 8.7 Hz, 1H), 7.74 (t, *J* = 7.5 Hz, 1H), 7.57 (s, 1H), 7.53 (d, *J* = 7.8 Hz, 1H), 7.36 (t, *J* = 7.6 Hz, 1H), 7.20 (d, *J* = 7.6 Hz, 1H), and 2.44 (s, 3H). ^13^C NMR (100 MHz, MeOH-d4): *δ* 181.8, 147.3, 146.7, 141.5, 139.6, 138.5, 136.9, 136.8, 133.9, 132.0, 131.2, 128.6, 128.0, 127.4, 126.0, 125.4, 124.1, 123.9, 121.4, 120.4, 119.9, 115.9, 115.0, 113.4, and 20.1. HRMS-ESI, *m*/*z*: [M]^+^ calculated for C_25_H_17_N_2_O^+^ 361.1335, obtained 361.1334.

For compound **25c**: red solid, 32%. ^1^H NMR (400 MHz, MeOH-d4): *δ* 9.38 (d, *J* = 6.2 Hz, 1H), 8.99 (d, *J* = 6.2 Hz, 1H), 8.43 (s, 1H), 8.34 (d, *J* = 8.0 Hz, 1H), 8.04–8.10 (m, 2H), 7.97 (t, *J* = 7.4 Hz, 1H), 7.86 (d, *J* = 0.6 Hz, 2H), 7.72–7.78 (m, 1H), 7.30–7.32 (m, 3H), and 2.30 (s, 3H). ^13^C NMR (100 MHz, MeOH-d4): *δ* 181.9, 147.5, 146.4, 141.6, 140.5, 137.6, 137.1, 136.1, 135.2, 132.1, 131.5, 130.2, 129.7, 127.6, 125.8, 125.6, 124.3, 124.1, 122.7, 120.6, 120.0, 116.0, 115.7, 112.9, and 19.5. HRMS-ESI, *m*/*z*: [M]^+^ calculated for C_25_H_17_N_2_O^+^ 361.1335, obtained 361.1429.

For compound **25d**: brown solid, 60%. ^1^H NMR (400 MHz, (CD_3_)_2_SO): δ 13.58 (br. s, 1H), 9.70 (s, 1H), 9.23 (s,1H), 9.05 (s, 1H), 8.49 (d, *J* = 4.0 Hz, 1H), 8.35–8.32 (m, 2H), 8.05–7.94 (m, 6H), 7.89 (d, *J* = 7.2 Hz, 1H), 7.72 (br. s, 1H), and 7.54 (t, *J* = 7.3 Hz, 2H). ^13^C NMR (100 MHz, (CD_3_)_2_SO): *δ* 182.2, 166.3, 147.0, 146.3, 140.6, 137.1, 136.5, 134.8, 133.4, 133.2, 132.2, 131.4, 128.7, 128.1, 127.5, 126.9, 126.6, 126.3, 125.6, 125.3, 125.0, 124.0, 122.9, 122.3, 120.6, 120.2, 115.7, 115.5, and 114.2. HRMS-ESI, *m*/*z*: [M]^+^ calculated for C_28_H_17_N_2_O^+^ 397.1335, obtained 397.1330.

For compound **25e**: red solid, 54%. ^1^H NMR (400 MHz, CD_3_OD): *δ* 9.32 (br. s, 1H), 8.99 (br. s, 1H), 8.59 (br. s, 1H), 8.33 (br. s, 1H), 8.07 (t, *J* = 3.5 Hz, 2H), 7.95–8.02 (m, 2H), 7.87–7.93 (m, 3H), 7.76 (t, *J* = 6.7 Hz, 2H), and 7.39–7.61 (m, 4H). ^13^C NMR (100 MHz, CD_3_OD): *δ* 181.8, 166.7, 147.3, 146.7, 141,4, 138.7, 136.9, 136.7, 136.2, 133.9, 131.5, 131.3, 129.1, 128.1, 127.9, 127.2, 126.5, 126.1, 125.7, 125.4, 125.1, 124.9, 124.6, 124.2, 120.1, 120.1, 115.1, and 113.0. HRMS-ESI, *m*/*z*: [M]^+^ calculated for C_28_H_17_N_2_O^+^ 397.1335, obtained 397.1339.

For compound **25f**: brown solid, 47%. ^1^H NMR (400 MHz, (CD_3_)_2_SO): *δ* 13.59 (br. s, 1H), 9.70 (s, 1H), 9.23 (s, 1H), 8.99 (s, 1H), 8.50 (s, 1H), 8.28 (d, *J* = 6.8, 1H), 8.07–8.02 (m, 2H), 7.91–7.84 (m, 5H), 7.76–7.75 (m, 3H), 7.50 (br. s, 2H), and 7.40 (t, *J =* 4.9, 1H). ^13^C NMR (100 MHz, (CD_3_)_2_SO): *δ* 182.2, 166.3, 147.0, 146.3, 140,5, 139.3, 139.2, 138.1, 137.0, 134.5, 133.1, 131.4, 129.0, 127.6, 127.2, 126.9, 126.5, 125.6, 124.0, 123.0, 121.9, 120.6, 120.1, 115.6, 115.5, and 114.1. HRMS-ESI, *m*/*z*: [M]^+^ calculated for C_30_H_19_N_2_O^+^ 423.1492, obtained 423.1490.

For compound **25g**: red solid, 57%. ^1^H NMR (400 MHz, MeOD): *δ* 9.35 (d, *J* = 6.2 Hz, 1H), 8.99 (d, *J* = 6.2 Hz, 1H), 8.62 (d, *J* = 1.0 Hz, 1H), 8.32 (d, *J* = 8.1 Hz, 1H), 8.11 (dd, *J* = 8.7, 1.6 Hz, 1H), 8.05 (d, *J* = 7.4 Hz, 1H), 7.97 (t, *J* = 7.8 Hz, 1H), 7.82 (d, *J* = 8.7 Hz, 1H), 7.74 (t, *J* = 7.5 Hz, 1H), 7.60 (d, *J* = 8.6 Hz, 2H), and 6.91 (d, *J* = 8.6 Hz, 2H). ^13^C NMR (100 MHz, MeOD): *δ* 181.9, 157.3, 147.4, 146.4, 141.5, 137.0, 136.8, 133.7, 131.3, 131.0, 127.9, 125.9, 125.4, 124.2, 120.5, 119.8, 115.5, 115.0, 113.4, and 112.0. HRMS-ESI, *m*/*z*: [M]^+^ calculated for C_24_H_15_N_2_O_2_^+^ 363.1128, obtained 363.1122.

For compound **25h**: red solid, 88%. ^1^H NMR (400 MHz, D_2_O): *δ* 9.04 (d, *J* = 6.2 Hz, 1H), 8.93 (s, 1H), 8.67 (d, *J* = 6.0 Hz, 3H), 8.50 (s, 1H), 7.91–8.04 (m, 3H), 7.78 (t, *J* = 7.6 Hz, 1H), 7.72 (d, *J* = 7.4 Hz, 1H), 7.62 (d, *J* = 8.6 Hz, 1H), and 7.48–7.54 (m, 1H). ^13^C NMR (100 MHz, D_2_O): *δ* 182.3, 147.0, 146.3, 143.0, 140.7, 139.6, 137.9, 133.0, 132.0, 131.7, 128.7, 127.3, 126.8, 126.1, 123.0, 122.3, 121.3, 120.7, 119.9, 116.0, 116.0, 115.3, and 114.4. HRMS-ESI, *m*/*z*: [M]^+^ calculated for C_23_H_15_N_3_O^+^ 348.1131, obtained 348.1125.

### 3.2. Biological Assay

#### 3.2.1. MIC Values Determination

The antimicrobial activities of fascaplysin (**1**) and its derivatives **7**, **15**, **16**, a**nd 25a**–**h** were studied in comparison to vancomycin on a wide panel of bacteria (*S. aureus* ATCC 29213, *B. cereus* ATCC 10702, *E. faecalis* ATCC 29212, *E. faecium* 132, *E. faecium* 130, *E. faecalis* 583 (VanR), MRSA 88, MRSA PE3R (resistant to doripenem), *S. epidermidis* 2001 MR, *S. aureus* 21555, *M. smegmatis* ATCC 607, and *E. coli* ATCC 25922). The minimum inhibitory concentration (MIC) for the test compounds was determined via the broth microdilution method according to CLSI guidelines [48], and vancomycin and rifampicin were used as controls. The reproducibility of the results of three independent repetitions did not extend beyond one dilution, which is acceptable for this method.

#### 3.2.2. Antiproliferative Activity against Tumor Cells

The K562 (human chronic myelognous leukemia) and A549 (human lung carcinoma) cell lines were purchased from the American Type Culture Collection (ATCC). The K562/4 (multi-drug-resistant (MDR) subline, kind gift of Dr. Alexander Shtil, Blokhin N.N. National Medical Research Center of Oncology, Moscow, Russia) was obtained via the stepwise selection of K562 cells for survival under the continuous exposure to doxorubicin. The reagents were purchased from Sigma-Aldrich (St. Louis, Missouri, USA), unless specified otherwise. The compounds (**1**, **7**, or **25a**–**h**) and doxorubicin (DOX) were dissolved in DMSO (AppliChem) as 10 mM stock solutions, followed by serial dilutions in water immediately before experiments. Adherent cells (A549) were cultured using in Dulbecco’s modified Eagle’s medium (PanEco, Moscow, Russia) supplemented with 10% fetal bovine serum (HyClone, Logan, Utah, USA), 2 mM L-glutamine, 100 U/mL penicillin, and 100 mg/mL streptomycin at 37 °C and 5% CO_2_ in a humidified atmosphere. Suspension cells (K-562 and K-562/4) were propagated in RPMI-1640 (PanEco) with the same supplements. Cells in the logarithmic phase were used in all experiments. The antiproliferative activity was determined in a formazan assay (MTT-test) using the standard method [49].

The cells placed were in a 96-well plate (Nalge NUNC International, Rochester, NY, USA) (5 × 10^3^ in 190 µL of culture medium) incubated for 24 h at 37 °C and 5% CO_2_ in a humidified atmosphere. Then, the cells were treated with 0.1% DMSO (vehicle control) or tested compounds (0.10–50 µM) and incubated for 72 h (each concentration was studied with three replications). Cells without drugs served as controls. After the completion of the incubation of 10 μL of aqueous solution, a MTT-reagent (MTT, PanEco; 5 mg/mL) was added to the cells. Then, the culture medium was removed from the cells (suspension cells K562 were pre-centrifuged on a plate centrifuge (Thermo FS, Waltham, MA, USA)), the cells were suspended in 100 μL of DMSO, and the optical density of the solutions was measured (microplate ELx800 photometer (BioTek, Winooski, Vermont, USA) at a wavelength of 570 nm). The number of living cells in each sample (N, %) was calculated as a percentage of the control using the formula N = (IE/IK) × 100%, where IE is the optical density in each experimental well and IK is the optical density in the control wells (samples not treated with test compounds, the values N of which are taken as 100%). Dose–response curves were plotted and analyzed. The IC_50_ (50% growth inhibitory concentration) was defined as the concentration of the compound that inhibited MTT conversion by 50%. The antiproliferative activity of the compounds was characterized by the concentration of half-maximal inhibition (IC_50_).

#### 3.2.3. Molecular Docking

Molecular docking of compounds **7**, **15**, and **16** was performed using Smina [50] with default parameters. We used the structure of FtsZ from *Staphylococcus aureus* from PDB ID 4DXD [36]. Protein structure processing included adding missing atoms and removing non-protein molecules using PDBFixer v. 1.8.1 (https://github.com/openmm/pdbfixer, accessed on 27 September 2023), adding hydrogens and converting them into the PDBQT format using Open Babel v. 3.1.1 [51]. SMILES of compounds **15**, **7**, and **16** were generated from the drawn 2D structure in Marvin JS v. 23.16.1 (https://marvinjs-demo.chemaxon.com/latest/demo.html, accessed on 27 September 2023) and then converted into 3D conformers in RDKit (release 2023.03.1, https://www.rdkit.org/, accessed on 27 September 2023). A single conformer was generated for every compound. Finally, using Open Babel, we added hydrogens and partial charges (MMFF94 force field) and saved ligands into the PDBQT format. The small molecule PC190723 (PDB ligand name 9PC) from PDB ID 4DXD was supplied to Smina for the generation of the binding site box with 10 Angstroms of buffer space around the ligand. Analysis of protein–ligand interactions was performed using PLIP v. 2.3.0 [52]. For molecular visualization, we used UCSF Chimera v. 1.16 [53]. Data and code are available through the link https://github.com/kluwik/fascaplysin_ftsz (accessed on 10 December 2023).

#### 3.2.4. In Vivo Efficiency Study of Antibacterial Activity

The animal study was performed in accordance with the European Convention for the Protection of Vertebrate Animals, Directive 86/609/EEC [54], the European Convention for Humane Methods for Animal Welfare and Maintenance [55], and the National Standard of the Russian Federation 33044-2014 “Good Laboratory Practice” [56]. The ethical aspects of animal experimentation were reviewed and approved by the local ethics committee of the Gause Institute of New Antibiotics, with protocol number 03/2021, dated 12 March 2021.

A comparative study of the efficacy of fascaplysin (**1**), 9-phenylfascaplysin (**7**) and vancomycin was carried out in a mouse staphylococcal sepsis model. Healthy female mice of the SHK colony weighing 18–20 g after a two-week quarantine period were randomized into groups (*n* = 10) and received *S. aureus* (strain 10, clinical isolate, adapted for growth in vivo via five-fold passaging in mice) as the infectious agent. In the experiment, female mice of the SHK colony weighing 18–20 g were used. Initially, the lethal dose (LD_100_) of staphylococcus was determined for this mouse line using the intravenous infection route. The mice deaths were counted daily for 14 days. Thus, the lethal dose (LD_100_) was defined as 8 × 10^8^ CFU/mouse. Afterwards, the mice were seated in cages of 10 heads and infected intravenously with S. aureus at a lethal dose, and the efficacy of the tested drugs was determined by the ED_50_ value (i.e., the dose at which 50% of the experimental animals survive). Then, 30 min after infection, the mice were injected intravenously with fascaplysin (**1**) or 9-phenylfascaplysin (**7**) at single doses ranging from 0.1 to 4.0 mg/kg or vancomycin at doses ranging from 2.5 to 7.5 mg/kg. As a control dose, a group of untreated animals infected with a lethal dose of *S. aureus* was present in the experiment. The determination of the ED_50_ of the tested compounds was carried out in one experiment under a single control using the Behrens method [57]. The animals were observed for 28 days, and the deaths were counted daily.

#### 3.2.5. In Vivo Study of Antitumor Activity

Animal procedures were performed using female CD-1 (total 145 animals) albino mice weighing 30 ± 3 g at 8–10 weeks of age, originally obtained from the vivarium of the Institute of Bioorganic Chemistry, Pushchino, Russia. All animals were housed in a standard animal facility under controlled environmental conditions at a temperature of 22 ± 2 °C and under a 12-hour light–dark cycle and controlled humidity of 50%, and the animals were provided with free access to water and a balanced diet in accordance with the National Standard of the Russian Federation 33216-2014 “Guidelines for accommodation and care of animals. Species-specific provisions for laboratory rodents and rabbits” [58]. Mice were kept in the following conditions: plastic cages, on a bed of small wood shavings, three individuals per 42 × 25 × 14 cm (length × height × width) cage, and in an open mode. Studies using experimental animals were performed under the European Commission’s legislation for the protection of animals used for scientific purposes (Directive 2010/63/EU on the protection of animals used for scientific purposes [55]) and in accordance with the rules of with and the National Standard of the Russian Federation 33044-2014 “Good Laboratory Practice” [56]. All experiments were approved by the Ethical Committee for Animal Research of the G.B Elyakov Pacific Institute of Bioorganic Chemistry of the Russian Academy of Science, with the protocol number 07/2021, dated 8 November 2021.

A murine model of ascite Ehrlich adenocarcinoma was presented. EACCs were obtained as described in the previous section. Experiments were performed using CD-1 albino mice weighing 31 ± 3 g. Mice were inoculated with 0.2 mL containing 1 × 10^6^ viable EACCs in the right hind limb (thigh) subcutaneously. The animals were randomized and divided into 4 groups (*n* = 10): (i) C ont(-), negative control; (ii) DOX, 0.25 mg/kg; (iii) compound **7**, 5 mg/kg; (iv) DOX, 0.25 mg/kg + compound **7**, 2.5 mg/kg. The treatment started when the primary tumor reached a size of 207–277 mm^3^. All tested remedies were injected i.p. as 0.5 mL (0.2 mL for Dox) portions of aqueous solutions (20% water–ethanol solution for compound **7**) for 5 days (one injection a day). Tumor volume was measured from the 6th day of tumor induction, and then every 4 days for a period of 20 days. Tumor growth was assessed by measuring the volume of the solid tumor using a digital calliper, and it was calculated using the formula:

$$V=\frac{\pi }{6}\times L\times W\times H,$$

where V, tumor volume; L, tumor length; W, tumor width; H, tumor height.

The evaluation of the chemotherapeutic efficacy of the tested remedies was carried out using the tumor growth index in each tumor-bearing group to determine the growth rate as a percentage of the value of a tumor over time. The tumor growth index was calculated using the formula:

$$Tumor\;Growth\;Index\left(\%\right)=\left[\frac{Vc-Vi}{Vi}\right]\times 100,$$

where Vc is the tumor volume on the 10, 13, 17, and 20 days after tumor inoculation, and Vi is the tumor volume on first treated day.

On the termination day (20nd day of tumor induction), experimental animals were euthanized via carbon dioxide inhalation, and the tumor mass was removed for visual assessment of the tumor. To assess the effectiveness of antitumor therapy using the studied drugs, the tumor growth inhibition (TGI, in %) in each treated group of animals was measured. TGI was calculated as described in [59] using the following formula:

$$TGI=\left(1-\frac{Te}{Tc}\right)\times 100$$

where Te means the weight of the treated tumors, and Tc means the weight of the tumors in negative control group of animals.

#### 3.2.6. Acute Toxicity

A toxicity assessment was performed using CD-1 albino mice, weighing 29 ± 2 g. MT was administered once intraperitoneally in doses of 10, 20, 30, and 40 mg/kg/mice (5 mice in each group). After the introduction of compound **7**, the animals were observed continuously for 1 day; then, the condition of the animals was noted 2 times a day for 14 days, and the number of dead animals was recorded. Mortality and changes in basic physiological parameters, such as body temperature, breathing frequency, wool characteristics, physical activity (motility, coordination and speed of movement), and behavioral responses (excitation or inhibition, eating and drinking water, grooming), were visually registered in each group of animals. LD_50_ values were calculated 14 days after administration using the following formula:

LD_50_ = LD_100_−ΣZd/n,

where LD_100_ is the maximum dose that causes the death of all animals in the group, Z is the arithmetic average of the number of animals in which mortality is noted under the influence of two adjacent doses, d is the interval between two adjacent doses, and n is the number of animals in each group [60].

### 3.3. Statistic Calculation

All data are expressed as mean ± SEM. The one-way analysis of variance (ANOVA), followed by Tukey’s post hoc test, was performed to determine statistical significance.

## 4. Conclusions

In this study, we assessed the therapeutic potential of a synthetic derivative **7** of the marine alkaloid fascaplysin, featuring a phenyl substituent at C-9. Our results revealed that this compound exhibits remarkably high antimicrobial activity against Gram-positive bacteria, including antibiotic-resistant strains, when examined in vitro. The positioning of a substituent at C-9 is of pivotal significance, as shifting it to adjacent positions results in a significant decline in antibacterial efficacy, underscoring the presence of specific therapeutic targets in Gram-positive bacteria. In an animal model of acute bacterial sepsis, we observed that the test compound possesses an ED_50_ value nine times lower than that of vancomycin. However, in vivo, the compound exhibits only a slightly superior efficacy compared to the unsubstituted fascaplysin, which is significantly less effective than compound **7** in vitro. Similarly, in tests of its antitumor activity against various variants of Ehrlich carcinoma in mice, the compound showed substantial effectiveness during the initial stages of treatment, but this efficacy decreased rapidly after the treatment course. Our data suggest that a key limitation of this compound lies in its rapid inactivation or clearance from the bloodstream or tissues, resulting in diminished effective concentrations. Additionally, the compound’s low solubility in water posed a significant challenge for in vivo testing. The introduction of polar fragments capable of forming hydrogen bonds to the phenyl substituent would overcome this limitation but led to a sharp decrease in the target activity in vitro. An increase in the volume of the lipophilic substituent also does not lead to an additional increase in activity in vitro. Therefore, further study of alternative chemical modifications of 9-phenylfascaplysin is necessary to improve its pharmacokinetic characteristics, for example, via the addition of polar sp^3^-containing substituents. Virtual modeling against FTS appears promising for predicting superior analogs to involve the substitution of the fascaplysin scaffold by sp^3^ groups or modification of the heterocyclic framework. The development of new drug formulations and the optimization of treatment regimens also can be necessary to harness the outstanding potential of this lead compound for practical usage.

## Figures and Tables

**Table 1:** Antimicrobial activity (MIC *, µg/mL) of fascaplysin (**1**) and its derivatives.

Compound	MIC, µg/mL											
	*S. aureus* ATCC 29213	*B. cereus* ATCC 10702	*E. faecalis* ATCC 29212	*E. faecium* 132	*E. faecium* 130 (VRE)	*E. faecalis* 583 (VRE)	*S. aureus* 88 (MRSA)	*S. aureus* PE3R (MRSA)	*S. epidermidis* 2001 MR	*S. aureus* 21555	*M. smegmatis* ATCC 607	*E. coli* ATCC 25922
Van	0.5	1.0	2.0	0.5	>32	32.0	0.5	1.0	1.0	2.0	-	-
Rif	0.018	0.25	-	-	-	-	-	-	-	-	0.03	8.0
**1**	1.0	0.125	8.0	1.0	1.0	≥8.0	1.0	0.5	0.0075	0.03	0.03	8.0
**7**	0.03	0.03	0.25	4.0	2.0	0.25	0.015	0.03	0.00375	0.03	0.25	8.0
**15**	0.125	2.0	16.0	8.0	8.0	16.0	0.5	0.5	0.0075	0.25	2.0	>16.0
**16**	0.125	0.125	0.25	8.0	16.0	0.13	0.125	0.06	0.03	0.03	1.0	>16.0
**25a**	0.06	0.03	0.25	8.0	8.0	0.125	0.03	0.03	0.03	0.03	0.5	>16.0
**25b**	0.06	0.06	0.125	0.5	0.5	0.125	0.03	0.06	0.015	0.03	0.5	>16.0
**25c**	0.25	0.25	2.0	16	16	2.0	0.25	0.25	0.25	0.25	4.0	>16.0
**25d**	0.06	0.5	0.5	16	8.0	1.0	0.125	0.06	0.015	0.06	8.0	>16.0
**25e**	0.06	0.25	0.125	0.5	0.5	0.25	0.06	0.06	0.015	0.06	0.5	8.0
**25f**	0.25	1.0	0.25	0.25	0.25	0.25	0.5	0.25	0.03	0.06	16.0	>16.0
**25g**	0.5	0.125	>16.0	16.0	16.0	>16.0	1.0	0.5	0.06	0.25	8.0	>16.0
**25h**	4.0	0.25	>16.0	>16.0	>16.0	>16.0	8.0	2.0	0.25	0.5	4.0	>16.0

* Modal values of minimum inhibitory concentration (MIC, µg/mL) of fascaplysin (**1**) and its derivatives determined via the broth microdilution method; vancomycin (Van) and rifampicin (Rif) were used as reference drugs.

**Table 2:** Antitumor activity of 9-phenylfascaplysin (**7**), DOX, and combinations of DOX and **7** on ascitic Ehrlich adenocarcinoma.

Group	MST, Days	ILS, %	Survival, %
Control (-)	16.1 ± 5.0	-	0
**7** (1.25 mg/kg)	24.9 ± 19.6	54.6	20
**7** (2.5 mg/kg)	31.9 ± 20.8	98.0	30
**7** (5.0 mg/kg)	58.4 ± 1.1 *	262.5	80
DOX (0.25 mg/kg)	62.0 ± 0.0 **	284.8	100
**7** + DOX (1.25 + 0.25 mg/kg)	54.0 ± 10.7 *	235.2	50

MST—mean survival time of animals with standard deviation; ILS—increase in life span, %; (-)—negative control (without treatment); DOX—doxorubicin treatment; **7**—9-phenylfascaplysin treatment; **7** + DOX—co-administration of doxorubicin and 9-phenylfascaplysin. Results are presented as mean ± SEM, *n* = 10 for each group. The significance of differences was estimated via one-way ANOVA, followed by Tukey’s test versus the saline group. Significant differences are presented as * *p* < 0.05 and ** *p* < 0.01.

**Table 3:** Antitumor efficacy of 9-phenylfascaplysin (**7**), DOX, and combinations of DOX and **7** against Ehrlich adenocarcinoma.

Group	Dose, mg/kg	Tumor Weight, g	TGI, %
Control (-)	-	2.54 ± 0.71	-
DOX	0.25	1.76 ± 0.94 *	31
**7**	5	1.75 ± 0.89 *	31
**7** + DOX	2.5 + 0.25	1.67 ± 0.75 *	34

TGI—tumor growth inhibition, %; Control (-)—negative control (without treatment); DOX—doxorubicin treatment; **7**—9-phenylfascaplysin treatment; **7** + DOX—co-administration of doxorubicin and 9-phenylfascaplysin. Results are presented as mean ± SEM, *n* = 10 for each group. The significance of differences was estimated via one-way ANOVA, followed by Tukey’s test versus the saline group. Significant differences are presented as * *p* < 0.05.

**Table 4:** Body weight changes in mice implanted with EACCs.

Group	Body Weight of Animals, g			
	Initial	6 Day	13 Day	20 Day
Saline	32.74 ± 3.09	32.32 ± 3.65	30.71 ± 5.70	27.56 ± 6.95
**7** (5.00 mg/kg)	30.04 ± 2.69	30.11 ± 3.90	29.06 ± 4.85	28.49 ± 7.14
DOX (0.25)	31.48 ± 2.90	31.36 ± 3.25	29.95 ± 5.31	28.05 ± 4.91
**7** + DOX (2.50 + 0.25)	30.12 ± 3.31	30.10 ± 2.53	29.84 ± 2.53	27.94 ± 5.79

Body weight was measured on days 6, 13, and 20 after tumor inoculation. Saline, negative control (without treatment); DOX, doxorubicin treatment; **7**, 9-phenylfascaplysin treatment; **7** + DOX, joint administration of doxorubicin and 9-phenylfascaplysin. Results are presented as mean ± SEM, *n* = 10 for each group.

**Table 5:** Acute toxicity of LD_50_ and MTD (LD_10_) of 9-phenylfascaplysin (**7**) in mice.

Parameter	9-Phenylfascaplysin (7)
LD_50_ (mg/kg)	25
MTD (LD_10_) (mg/kg)	12

## Data Availability

The original data are available from the corresponding author on request.

## Supplementary Materials

The following supporting information can be downloaded via this link: https://www.mdpi.com/article/10.3390/md22020053/s1. Table S1. Antimicrobial and antiproliferative activities (MIC and IC_50_ correspondingly) of fascaplysin (**1**) and its derivatives in vitro. Table S2. Efficacy (ED_50_ value, mg/kg) of 9-phenylfascaplysin (**7**), fascaplysin (**1**) and vancomycin (Van) on a mouse model of staphylococcal sepsis. Spectra Data.
