# Supplementary material for: Comparative Evaluation of the Antibacterial and Antitumor Activities of 9-Phenylfascaplysin and Its Analogs
Source: Mar Drugs. 2024 Jan 23;22(2):53. doi: 10.3390/md22020053 (PMC10890213; doi:10.3390/md22020053)

Supporting Information

Comparative Evaluation of the Antibacterial and Antitumor Activities of 9-Phenylfascaplysin and Its Analogs

Maxim E. Zhidkov 1,*, Maria A. Sidorova 1, Polina A. Smirnova 1, Oleg A. Tryapkin 1, Andrey V. Kachanov 1, Alexey V. Kantemirov 1, Lyubov G. Dezhenkova 2, Natalia E. Grammatikova 2, Elena B. Isakova 2, Andrey E. Shchekotikhin 2, Marina A. Pak 1, Olga N. Styshova 3, Anna A. Klimovich 3 and Aleksandr M. Popov 3

1 Department of Chemistry and Materials, Institute of High Technologies and Advanced Materials, FEFU Cam-pus, Far Eastern Federal University, Ajax Bay 10, Russky Island, 690922 Vladivostok, Russia;

sidorova_ma@dvfu.ru (M.A.S.); smirnova_pa@dvfu.ru (P.A.S.); triapkin_oa@dvfu.ru (O.A.T.);

kachanov.av@dvfu.ru (A.V.K.); t0ym45ter@gmail.com (A.V.K.); marina.a.pak@mail.ru (M.A.P.)

2 Laboratory of Chemical Transformation of Antibiotics, Gause Institute of New Antibiotics,

119021 Moscow, Russia; dezhenkovalg@yahoo.com (L.G.D.); ngrammatikova@yandex.ru (N.E.G.);

ebisakova@yandex.ru (E.B.I.); shchekotikhin@mail.ru (A.E.S.)

3 Departments of Biotechnology and Marine Natural Compounds Chemistry, G.B. Elyakov Pacific Institute of Bioorganic Chemistry, Far Eastern Branch of The Russian Academy of Sciences, 690922 Vladivostok, Russia; krivoshapkoon@mail.ru (O.N.S.); annaklim_1991@mail.ru (A.A.K.); popovam@piboc.dvo.ru (A.M.P.)

* Correspondence: zhidkov.me@dvfu.ru

Contents

Table S1. Antimicrobial and antiproliferative activities (MIC and IC_{50} correspondingly) of fascaplysin (**1**) and its derivatives *in vitro* 3-4

Table S2. Efficacy (ED_{50} value, mg/kg) of 9-phenylfascaplysin (**7**), fascaplysin (**1**) and vancomycin (Van) on a mouse model of staphylococcal sepsis 5

Spectra Data ……………………………………… ………………..……..… 6-59

Table S1. Antimicrobial and antiproliferative activities (MIC[*] and IC_{50}[**] correspondingly) of fascaplysin *(1)* and its derivatives *in vitro*.

Compound	MIC, µg/mL												IC_{50}, µM		
	***S. aureus* ATCC 29213**	***B. cereus* ATCC 10702**	***E. faecalis* ATCC 29212**	***E. faecium* 132**	***E. faecium* 130 (VRE)**	***E. faecalis* 583 (VRE)**	***S. aureus* 88 (MRSA)**	***S. aureus* PE3R (MRSA)**	***S. epidermidis* 2001 MR**	***S. aureus* 21555**	***M. smegmatis* ATCC 607**	***E. coli* ATCC 25922**	**K-562**	**K-562/4**	**A-549**
**Van**	0.5	1.0	2.0	0.5	>32	32.0	0.5	1.0	1.0	2.0	-	-	-	-	-
**Rif**	0.018	0.25	-	-	-	-	-	-	-	-	0.03	8.0	-	-	-
**DOX**	-	-	-	-	-	-	-	-	-	-	-	-	0.40±0.05	6.4±0.8	0.40±0.06
**1**	1.0	0.125	8.0	1.0	1.0	≥8.0	1.0	0.5	0.0075	0.03	0.03	**8.0**	0.19±0.03	0.30±0.05	0.20±0.03
**7**	**0.03**	**0.03**	**0.25**	**4.0**	**2.0**	**0.25**	**0.015**	**0.03**	**0.00375**	**0.03**	**0.25**	**8.0**	**0.10±0.02**	**0.22±0.03**	**0.20±0.02**
**15**	0.125	2.0	16.0	8.0	8.0	16.0	0.5	0.5	0.0075	0.25	2.0	>16.0	1.2±0.2	4.0±0.5	0.60±0.08
**16**	0.125	0.125	0.25	8.0	16.0	0.13	0.125	0.06	0.03	0.03	1.0	>16.0	0.60±0.08	3.2±0.4	2.1±0.3
**25a**	0.06	0.03	0.25	8.0	8.0	0.125	0.03	0.03	0.03	0.03	0.5	>16.0	1.6±0.2	1.3±0.2	0.50±0.06
**25b**	0.06	0.06	0.125	0.5	0.5	0.125	0.03	0.06	0,015	0.03	0.5	>16.0	1.1±0.2	1.5±0.2	0.60±0.07

**25c**	0.25	0.25	2.0	16	16	2.0	0.25	0.25	0.25	0.25	4.0	>16.0	0.60±0.06	0.8±0.1	0.30±0.04
**25d**	0.06	0.5	0.5	16	8.0	1.0	0.125	0.06	0.015	0.06	8.0	>16.0	2.2±0.3	2.3±0.3	1.4±0.2
**25e**	0.06	0.25	0.125	0.5	0.5	0.25	0.06	0.06	0.015	0.06	0.5	8.0	0.50±0.07	0.8±0.1	0.30±0.04
**25f**	0.25	1.0	0.25	0.25	0.25	0.25	0.5	0.25	0.03	0.06	16.0	>16.0	4.5±0.6	3.9±0.5	0.25±0.03
**25g**	0.5	0.125	>16.0	16.0	16.0	>16.0	1.0	0.5	0.06	0.25	8.0	>16.0	1.2±0.2	1.3±0.2	3.8±0.5
**25h**	4.0	0.25	>16.0	>16.0	>16.0	>16.0	8.0	2.0	0.25	0.5	4.0	>16.0	4.5±0.6	5.4±0.7	9.0±1.3

* Modal values of minimum inhibitory concentration (MIC).

** Mean 50% growth inhibitory concentration (IC_{50} ± S.D.) of 3 independent experiments, MTT-test.

Table S2. Efficacy (ED$_{50}$ value, mg/kg) of 9-phenylfascaplysin (7), fascaplysin (1) and vancomycin (Van) on a mouse model of staphylococcal sepsis.

**Compound**	**Dose, mg/kg**	**Survival*, %**	**ED$_{50}$, mg/kg**
Fascaplysin (**1**)	0.10	20	0.55
	0.25	40	
	0.50	50	
	1.0	70	
	2.0	70	
	4.0	80	
9-Phenylfascaplysin (**7**)	0.10	30	0.48
	0.25	40	
	0.50	50	
	1.0	70	
	2.0	80	
	4.0	90	
Van	2.5	30	4.2
	3.5	40	
	4.5	50	
	5.5	70	
	6.5	90	
	7.5	100	
Control dose *S. aureus*	-	0	

***n=10 for each group.**

Spectra Data

^{1}H NMR spectra of 1-(2'-bromobenzoyl)-6-iodo-β-carboline (9)

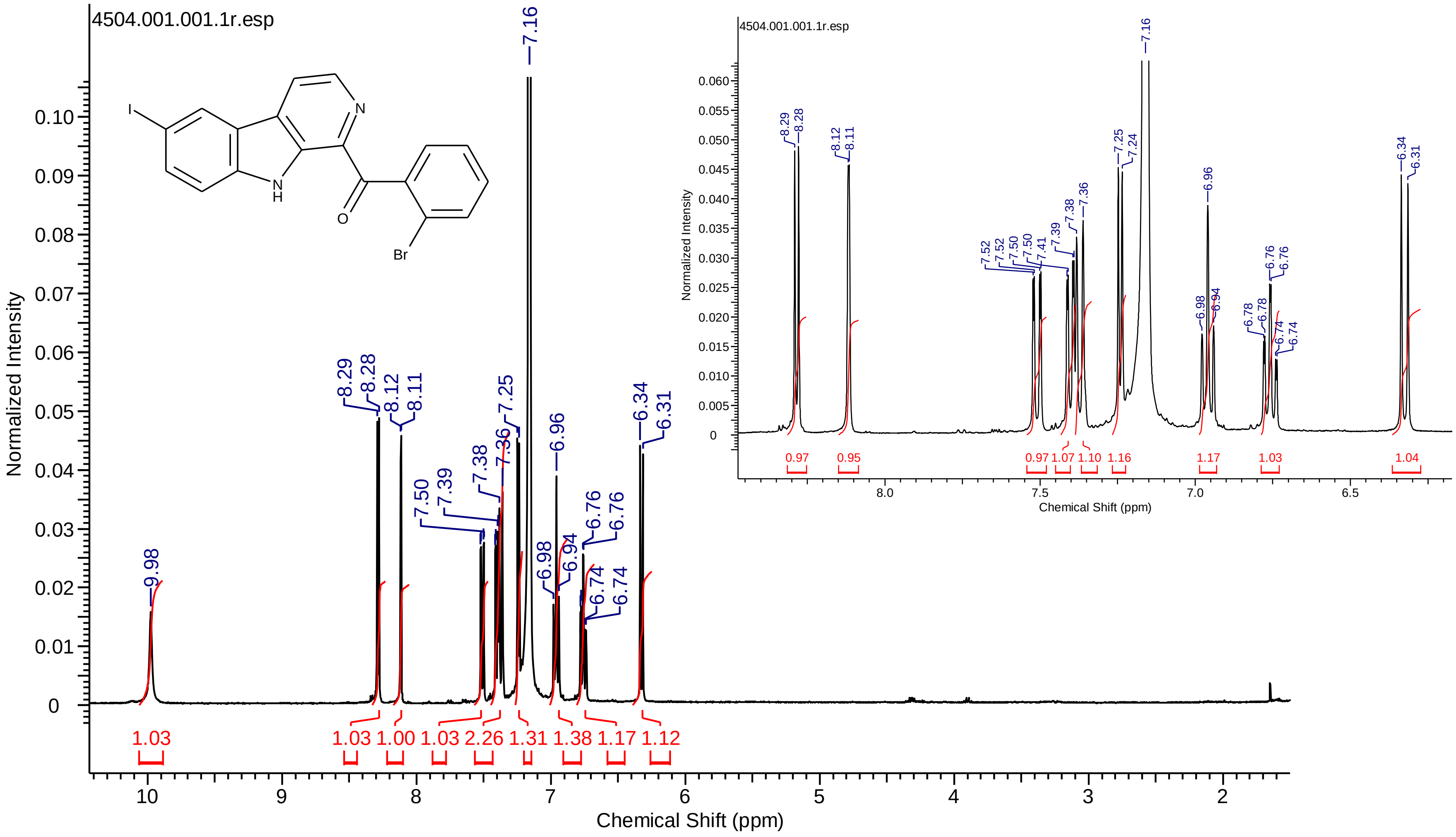

^{13}C NMR spectra of 1-(2'-bromobenzoyl)-6-iodo-β-carboline (9)

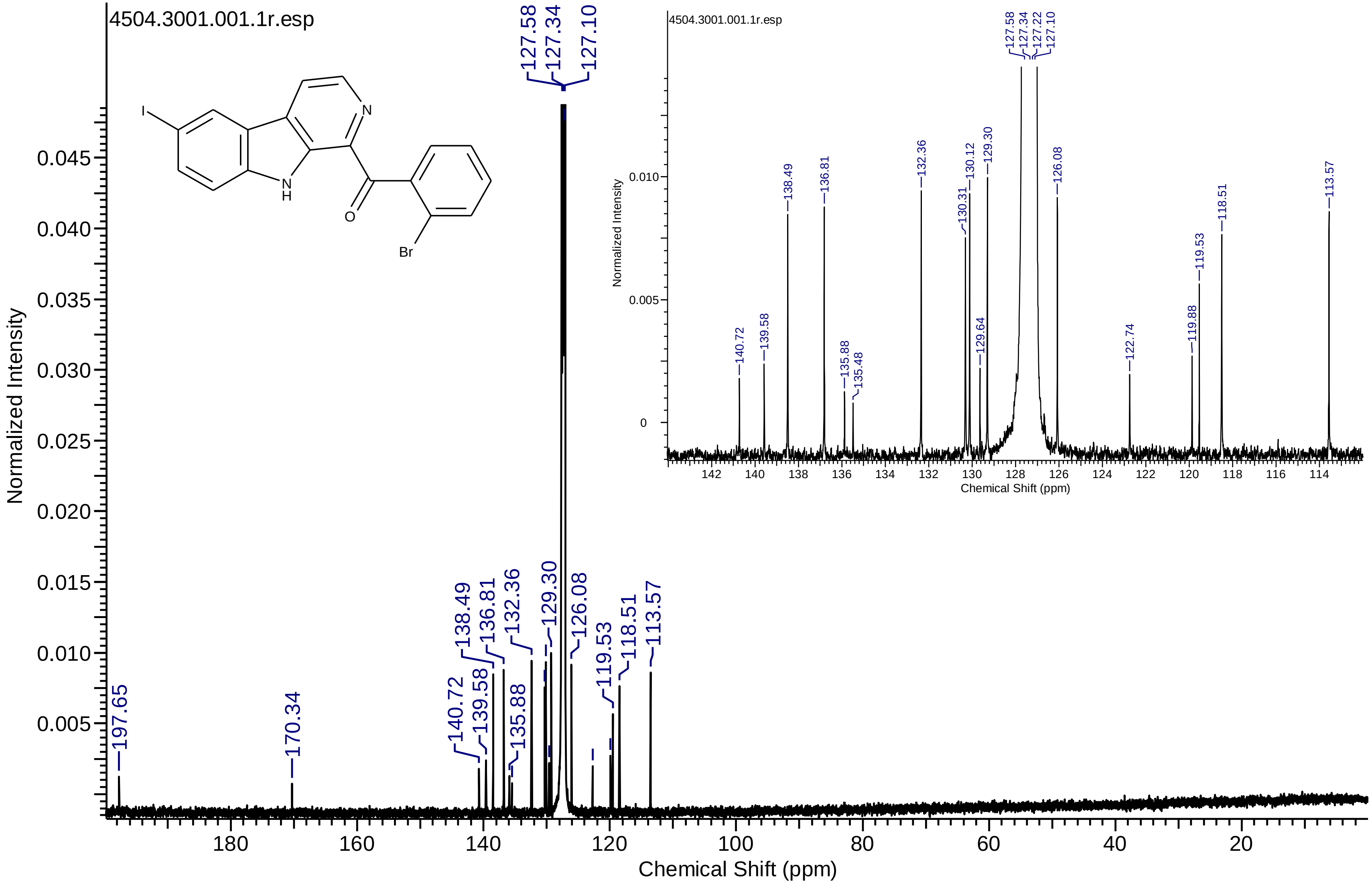

^{1}H NMR spectra of 1-(2’-chlorobenzoyl)-6-iodo-β-carboline (13)

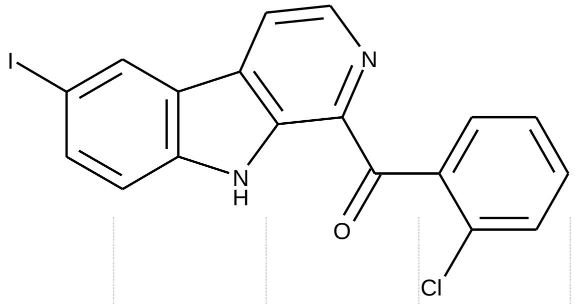

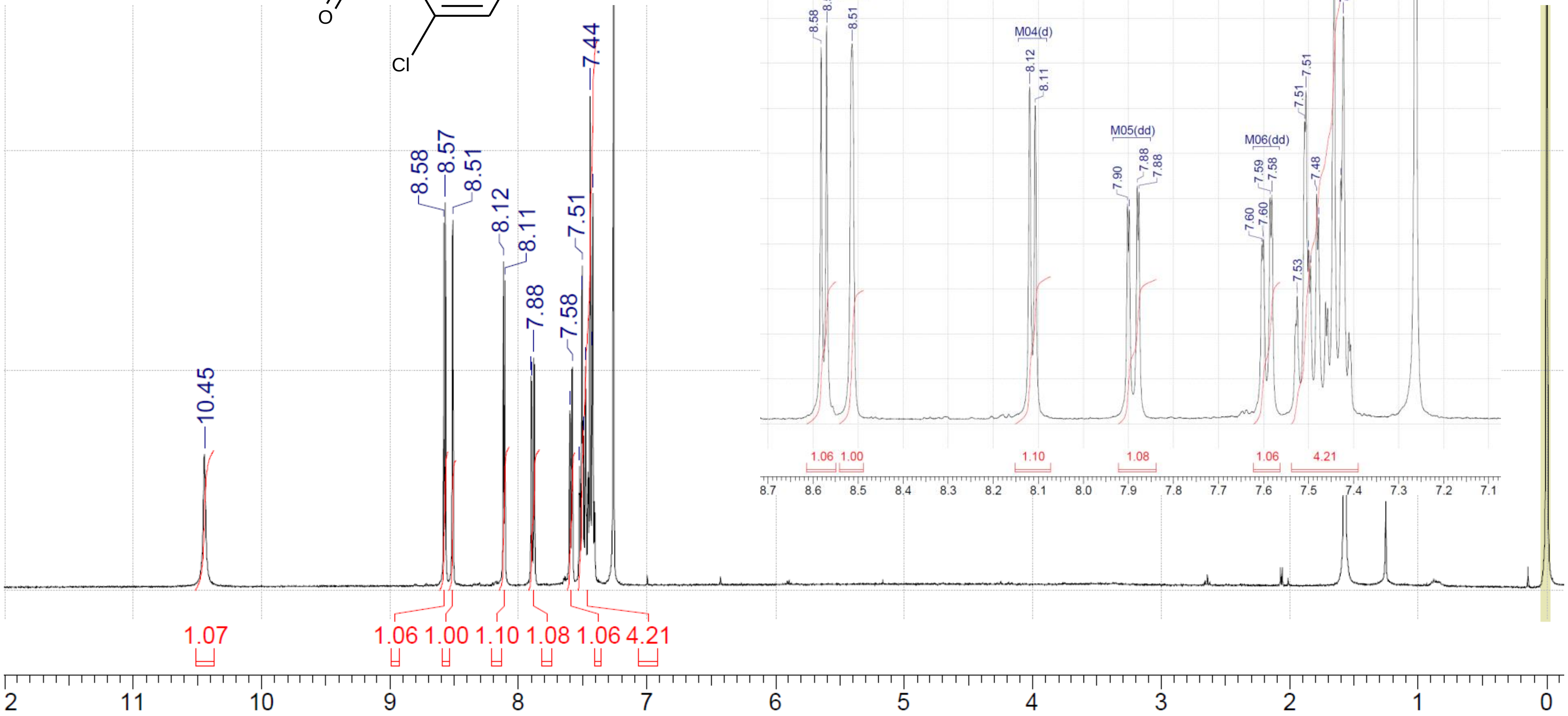

^{13}C NMR spectra of 1-(2'-chlorobenzoyl)-6-iodo-β-carboline (13)

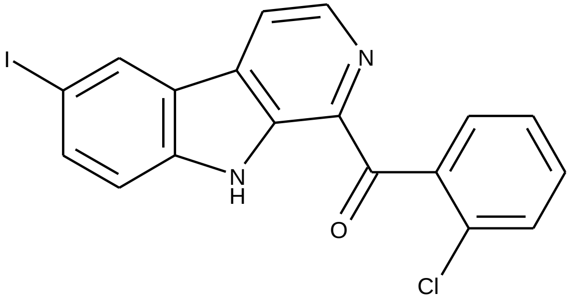

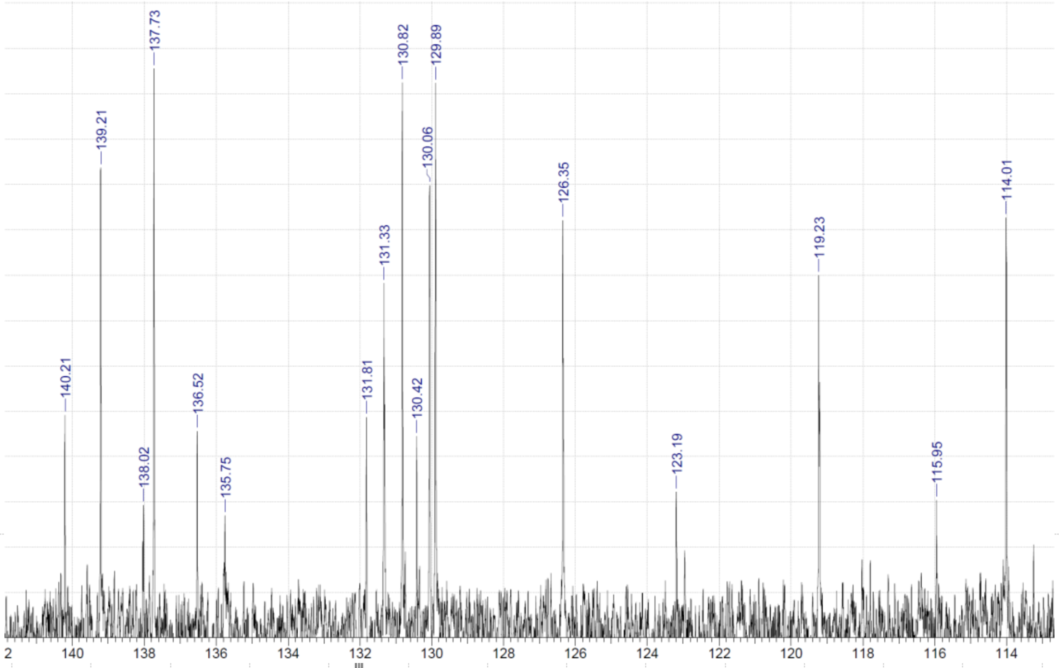
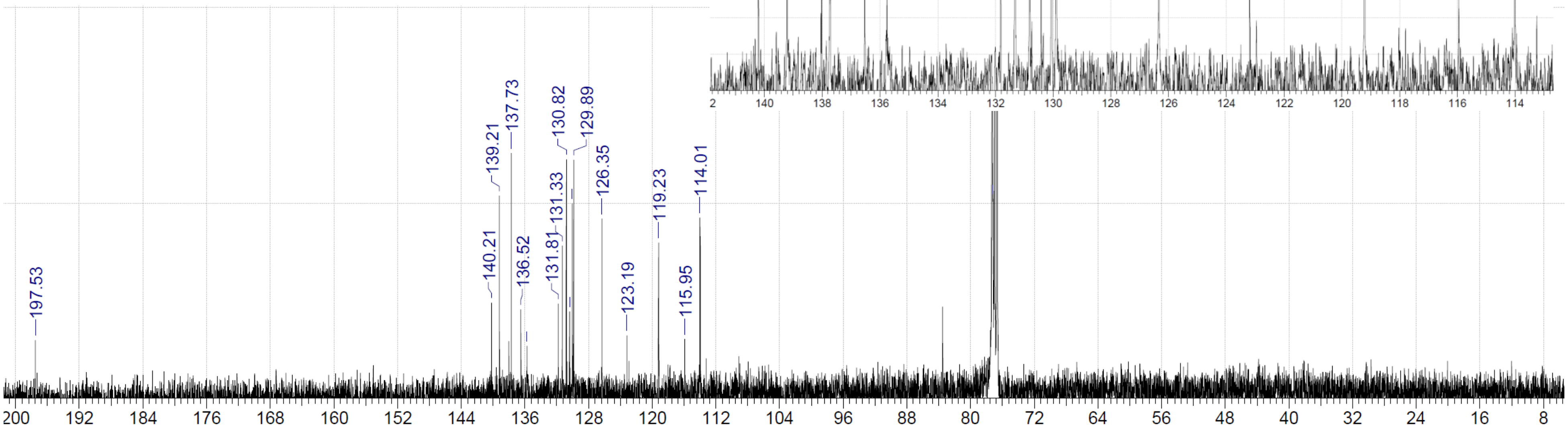

^{1}H NMR spectra of 1-(2'-bromobenzoyl)-6-phenyl-β-carboline (10)

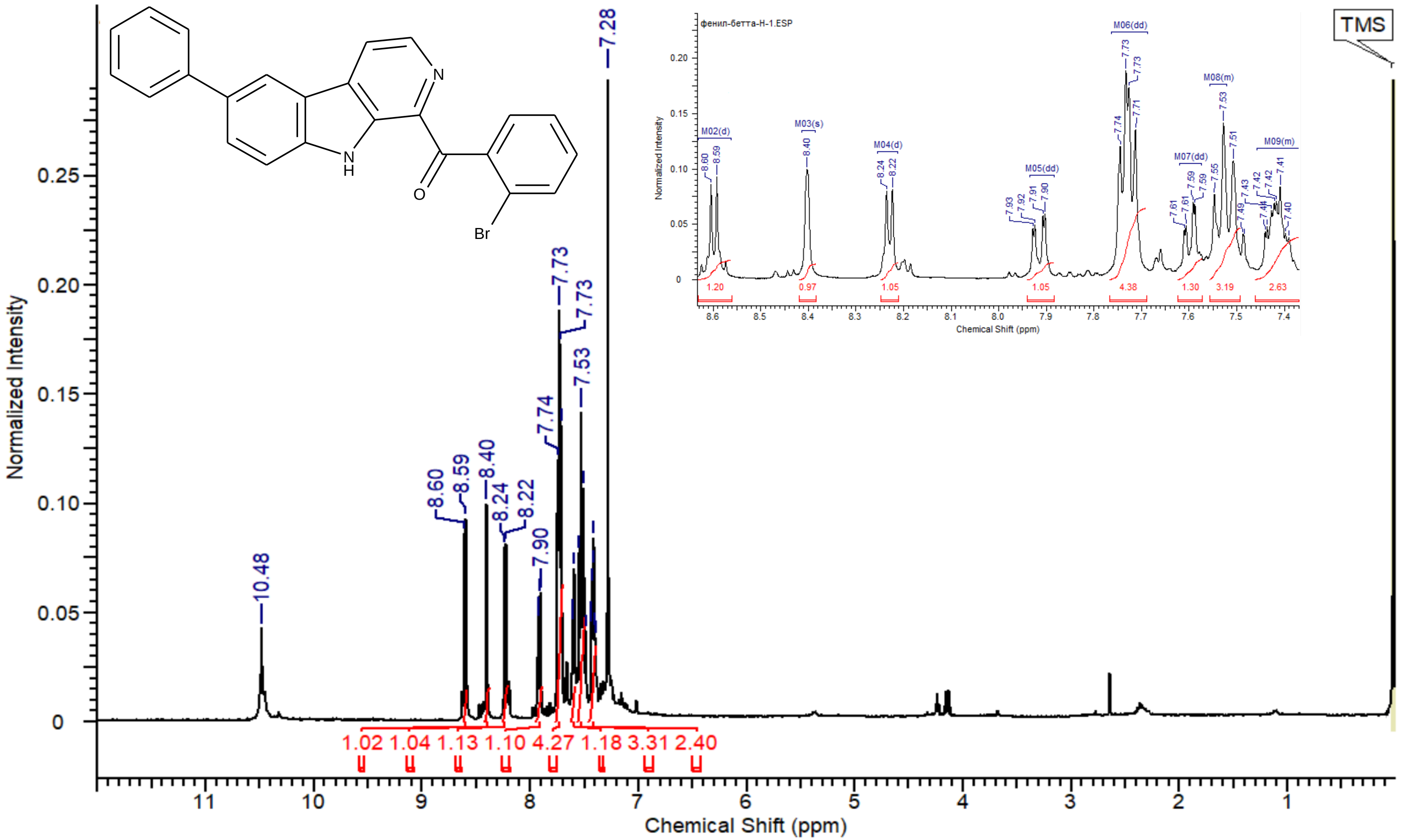

^{13}C NMR spectra of 1-(2'-bromobenzoyl)-6-phenyl-β-carboline (10)

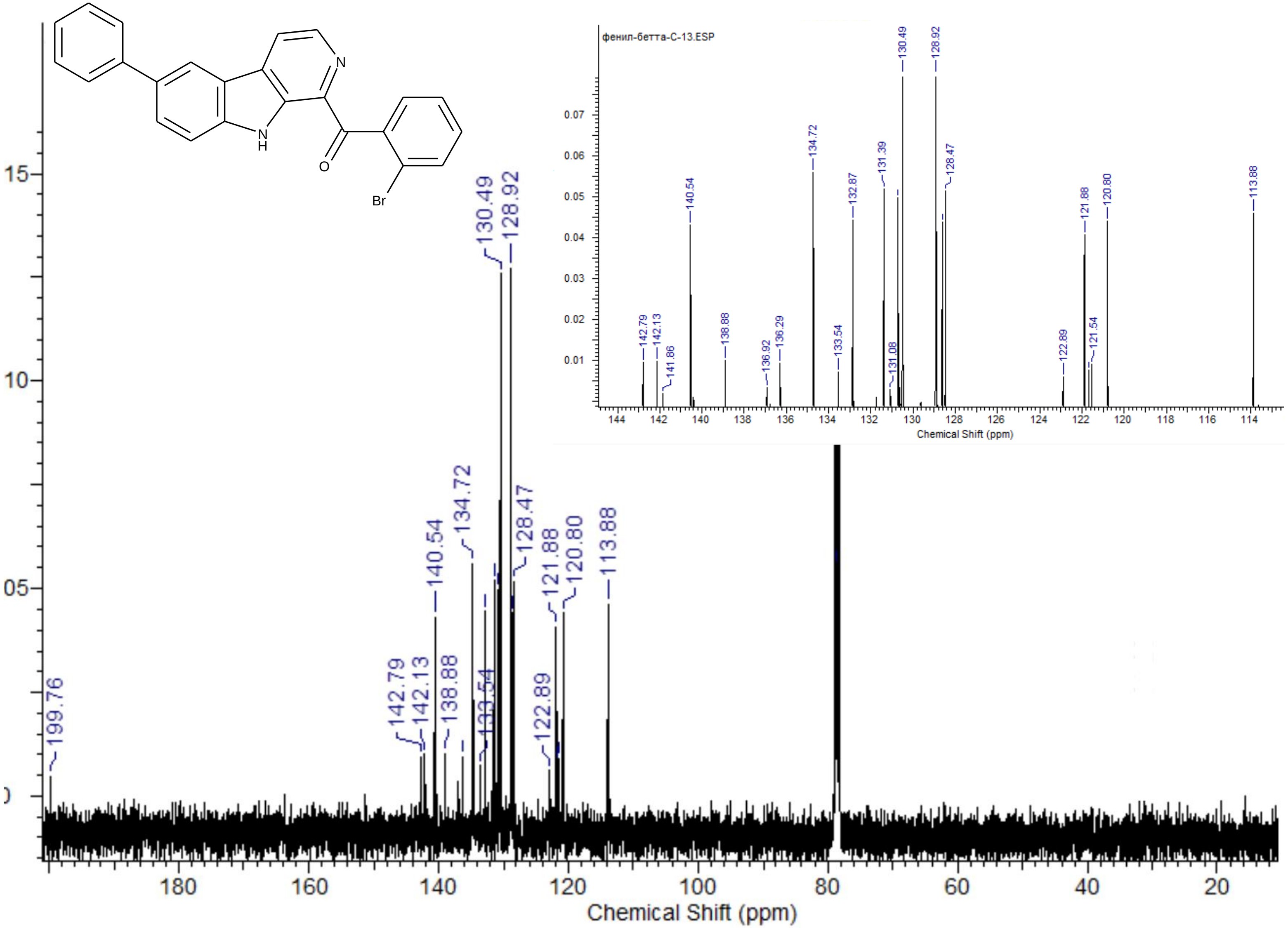

^{1}H NMR spectra of 9-phenylfascaplysin (7)

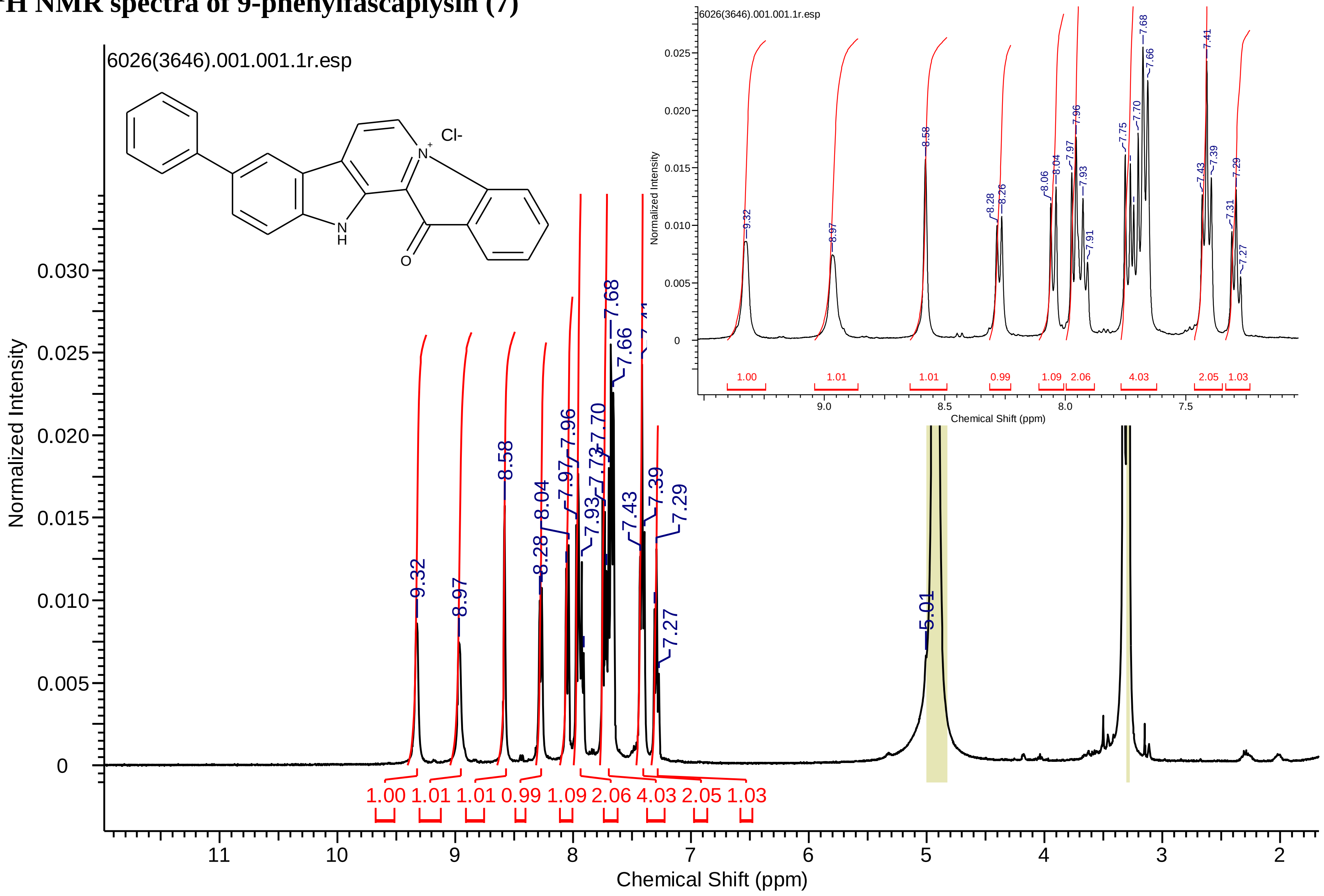

^{13}C NMR spectra of 9-phenylfascaplysin (7)

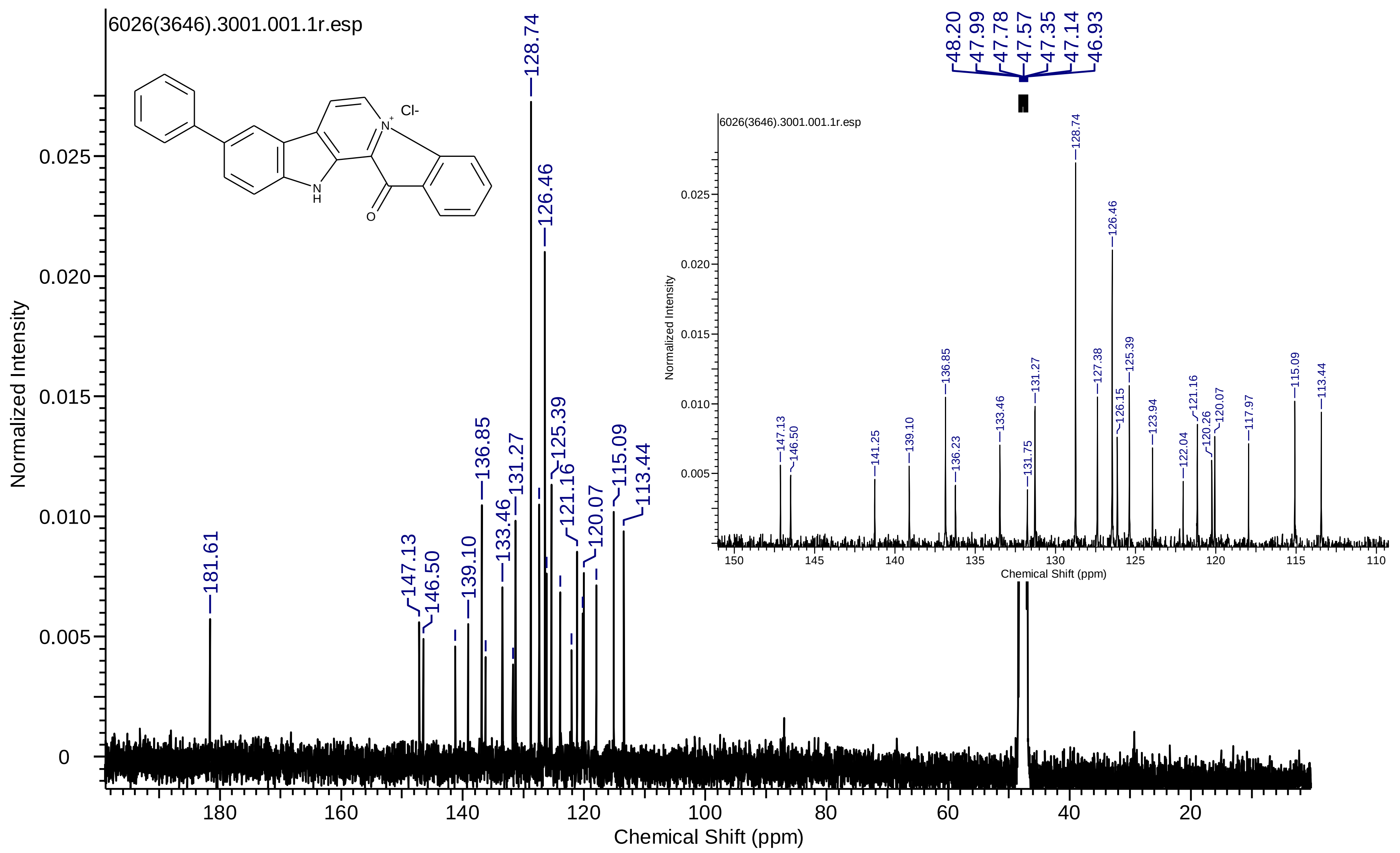

^{1}H NMR spectra of 5-bromo-1-(2-chlorobenzoyl)-β-carboline (20)

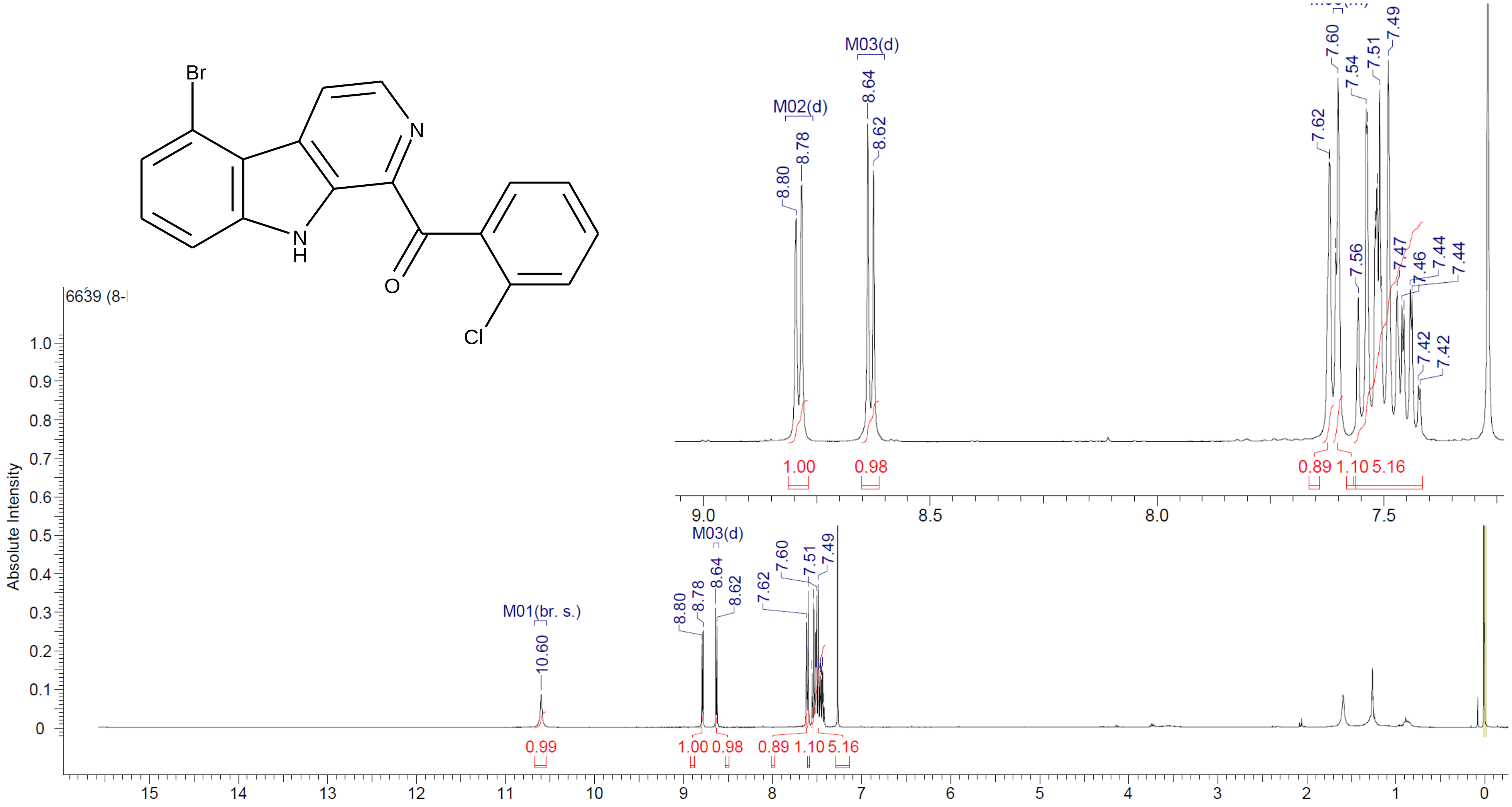

^{13}C NMR spectra of 5-bromo-1-(2-chlorobenzoyl)-β-carboline (20)

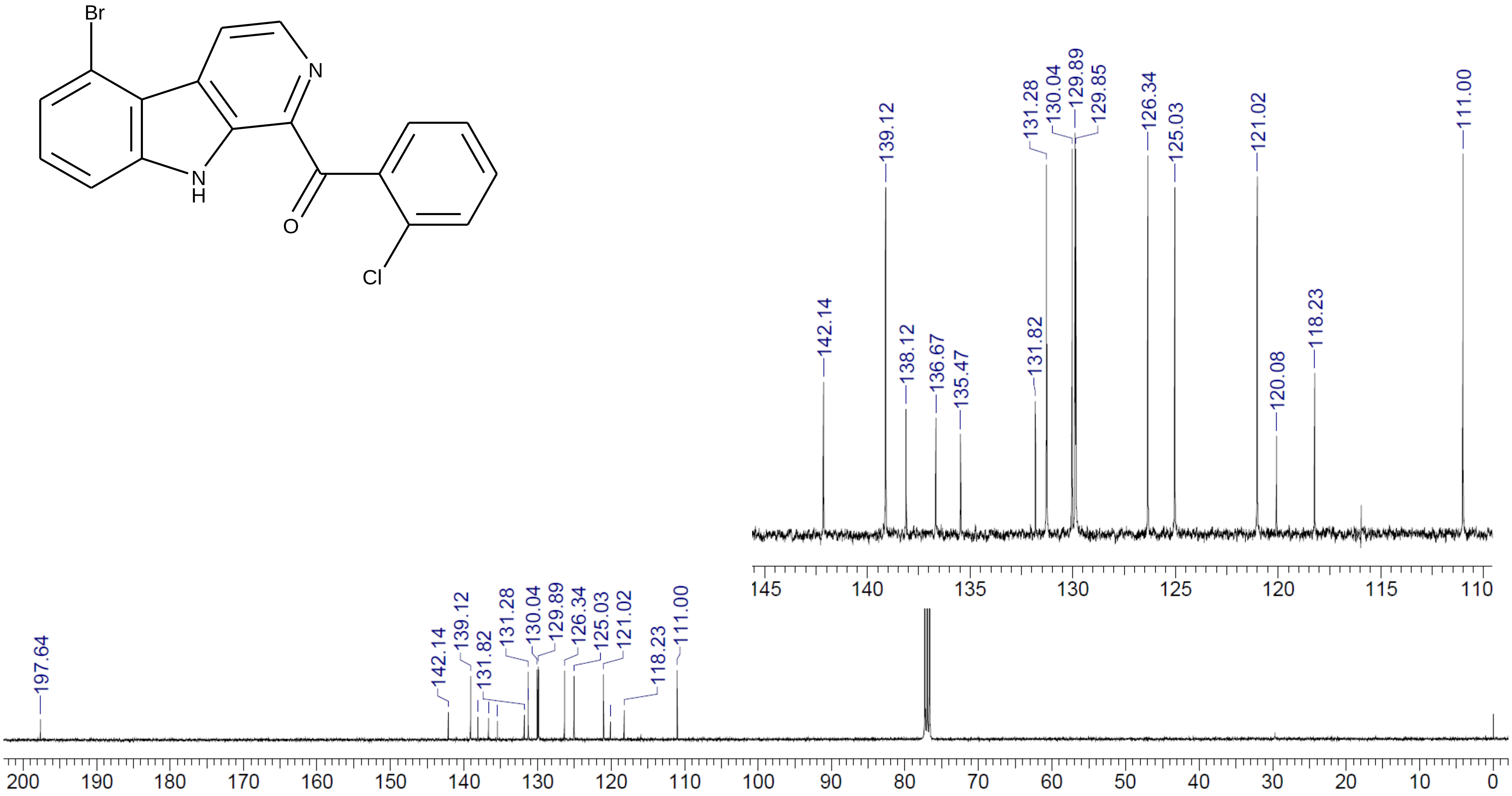

^{1}H NMR spectra of c7-bromo-1-(2-chlorobenzoyl)-β-carboline (21)

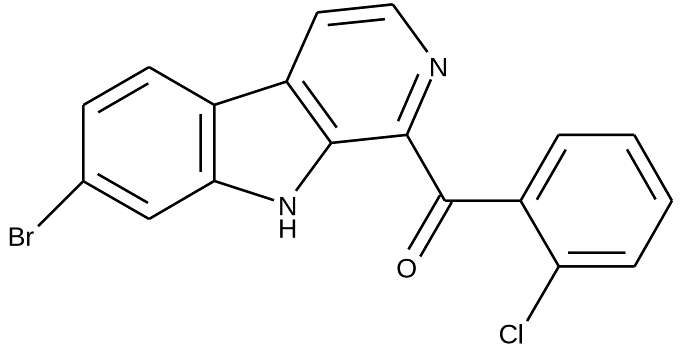

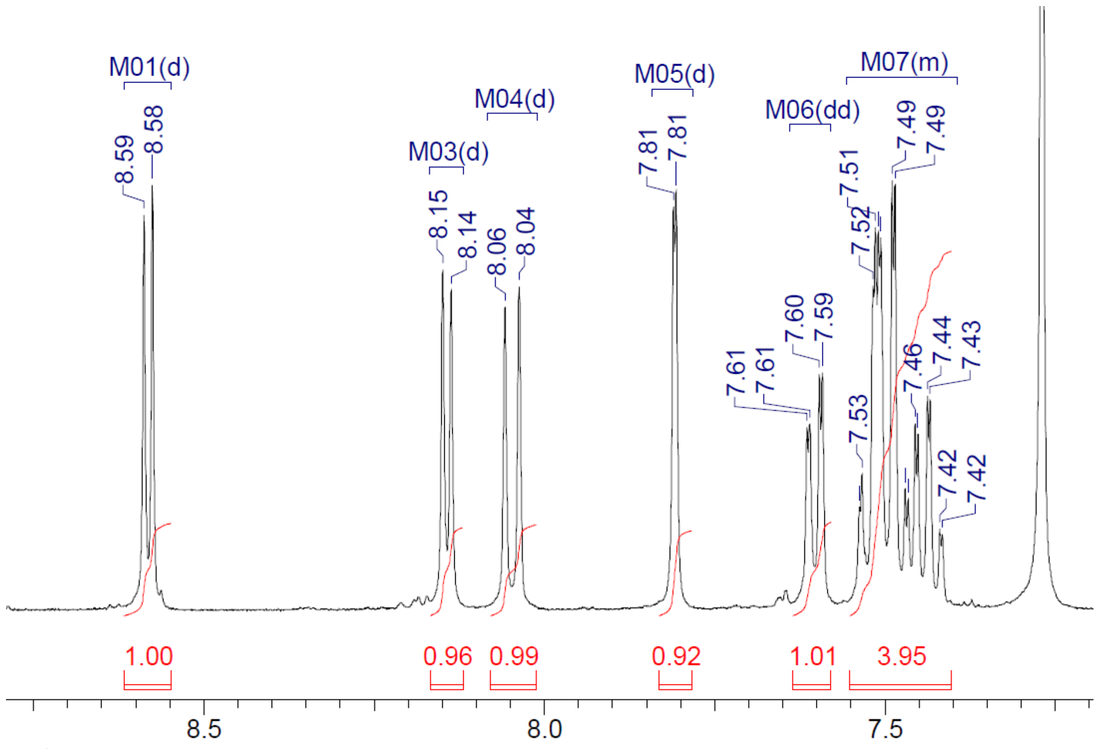

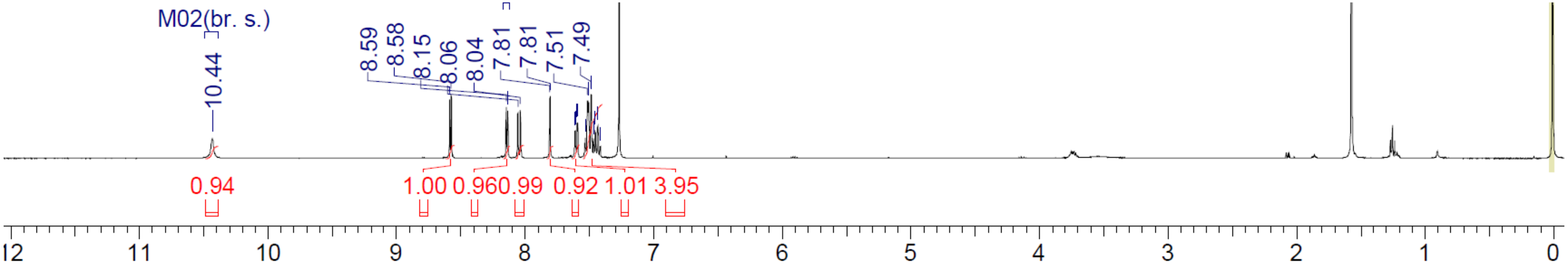

^{13}C NMR spectra of 7-bromo-1-(2-chlorobenzoyl)-β-carboline (21)

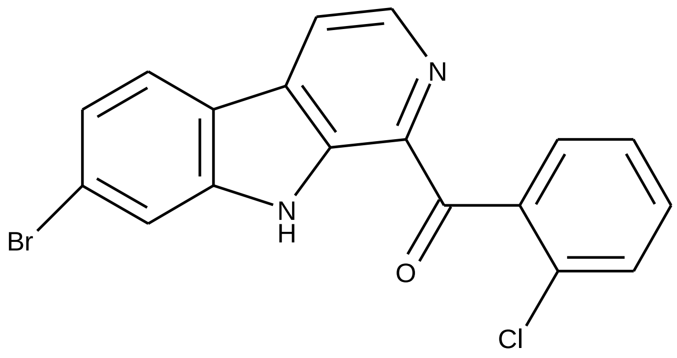

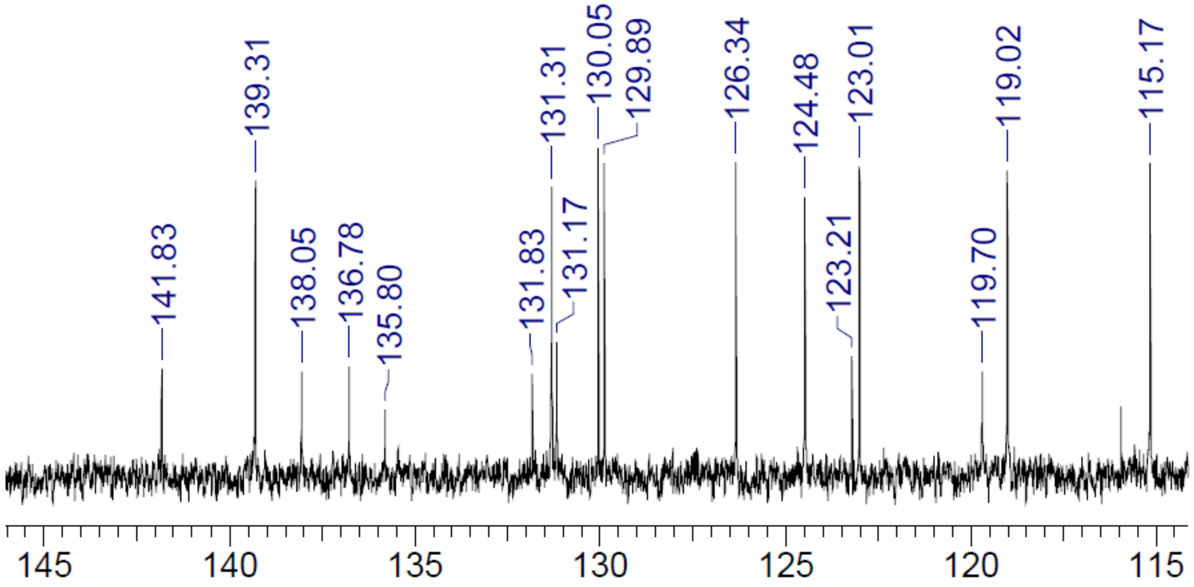

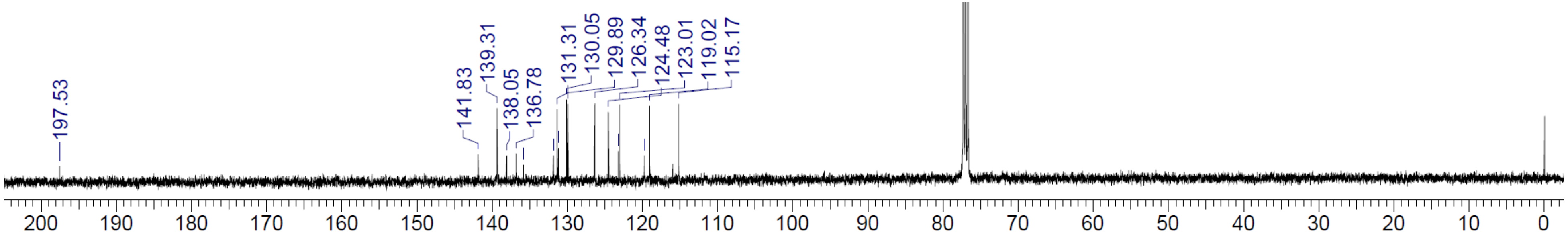

^{1}H NMR spectra of compound 14

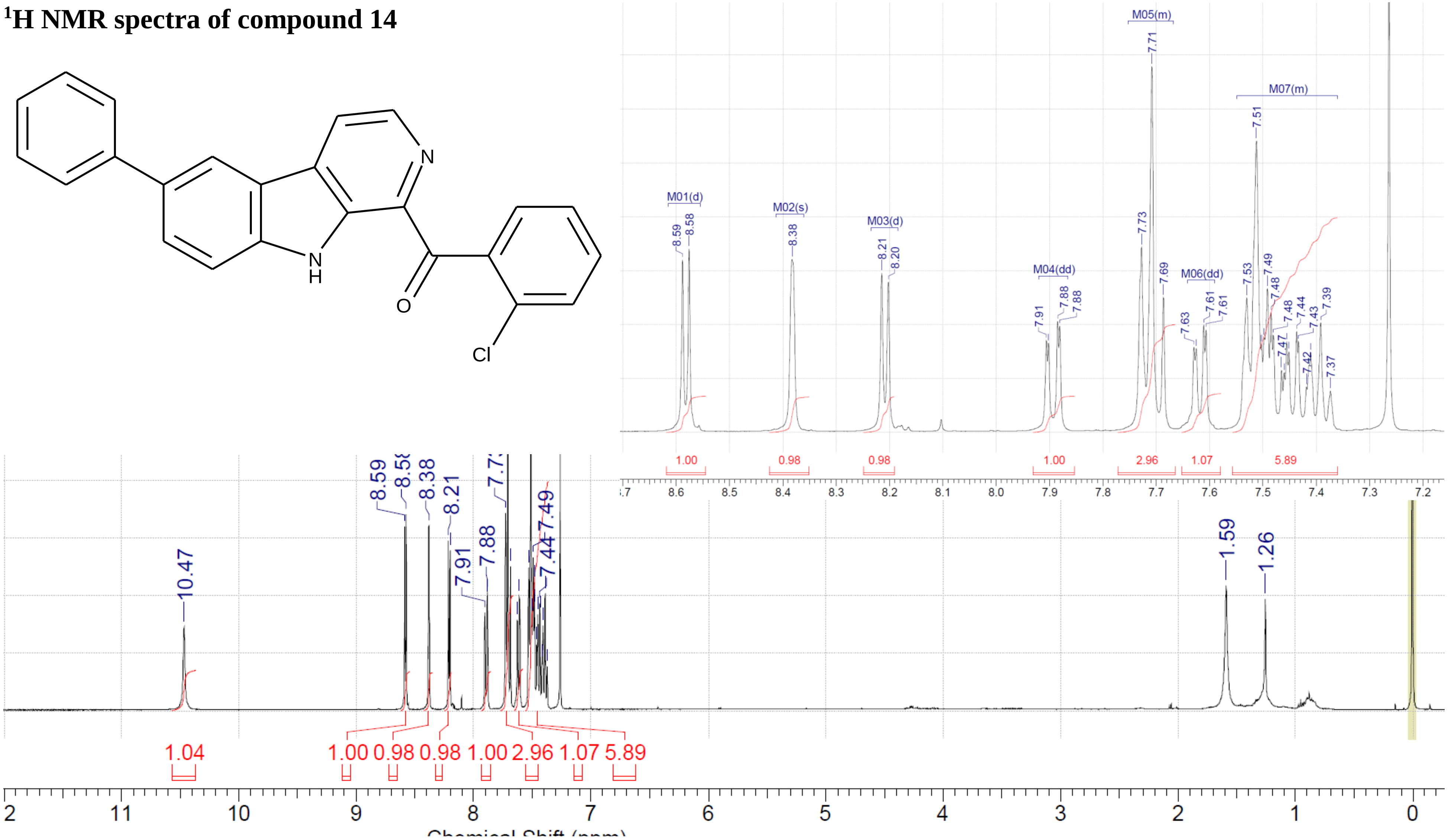

^{13}C NMR spectra of compound 14

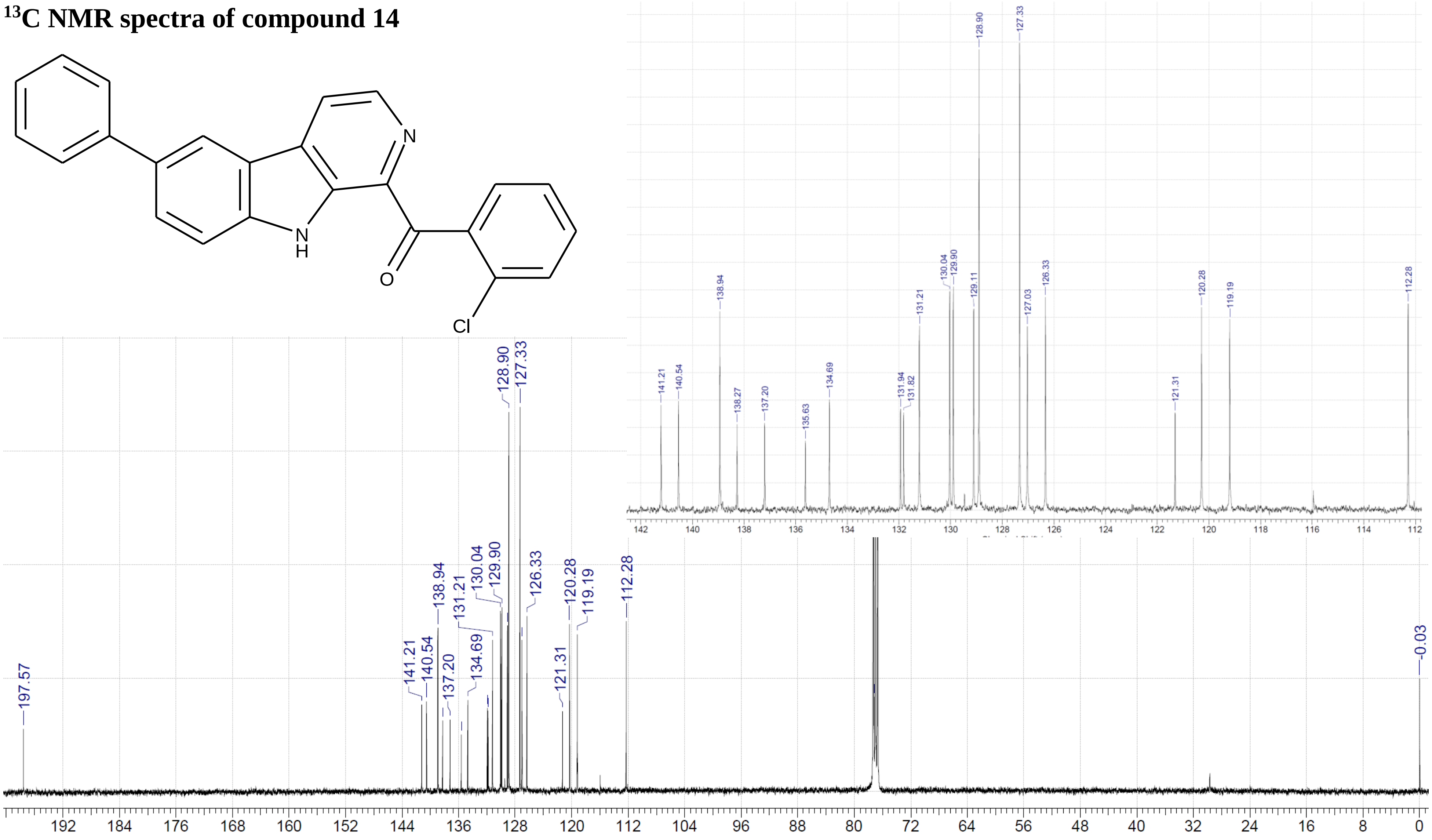

^{1}H NMR spectra of compound 22

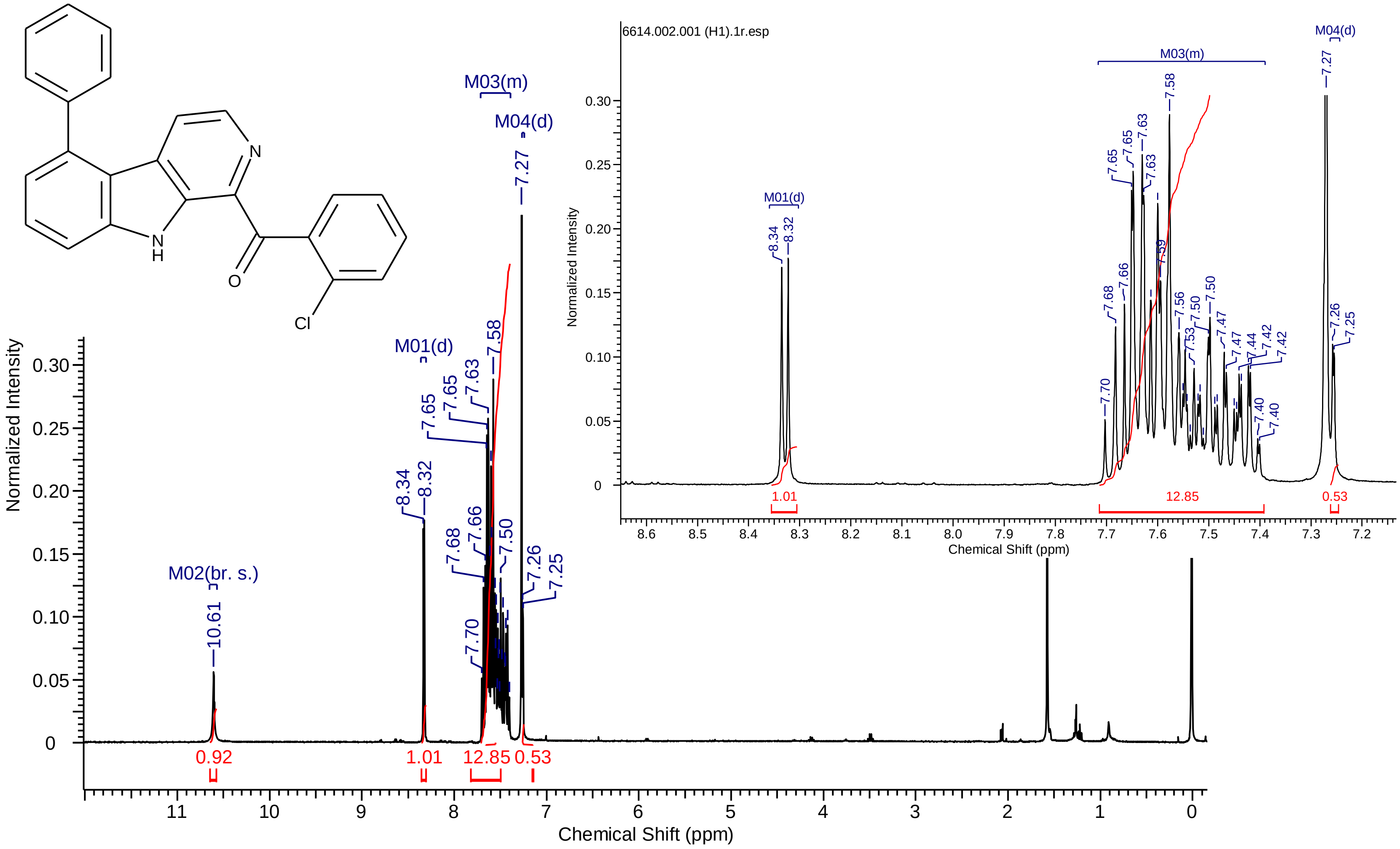

^{13}C NMR spectra of compound 22

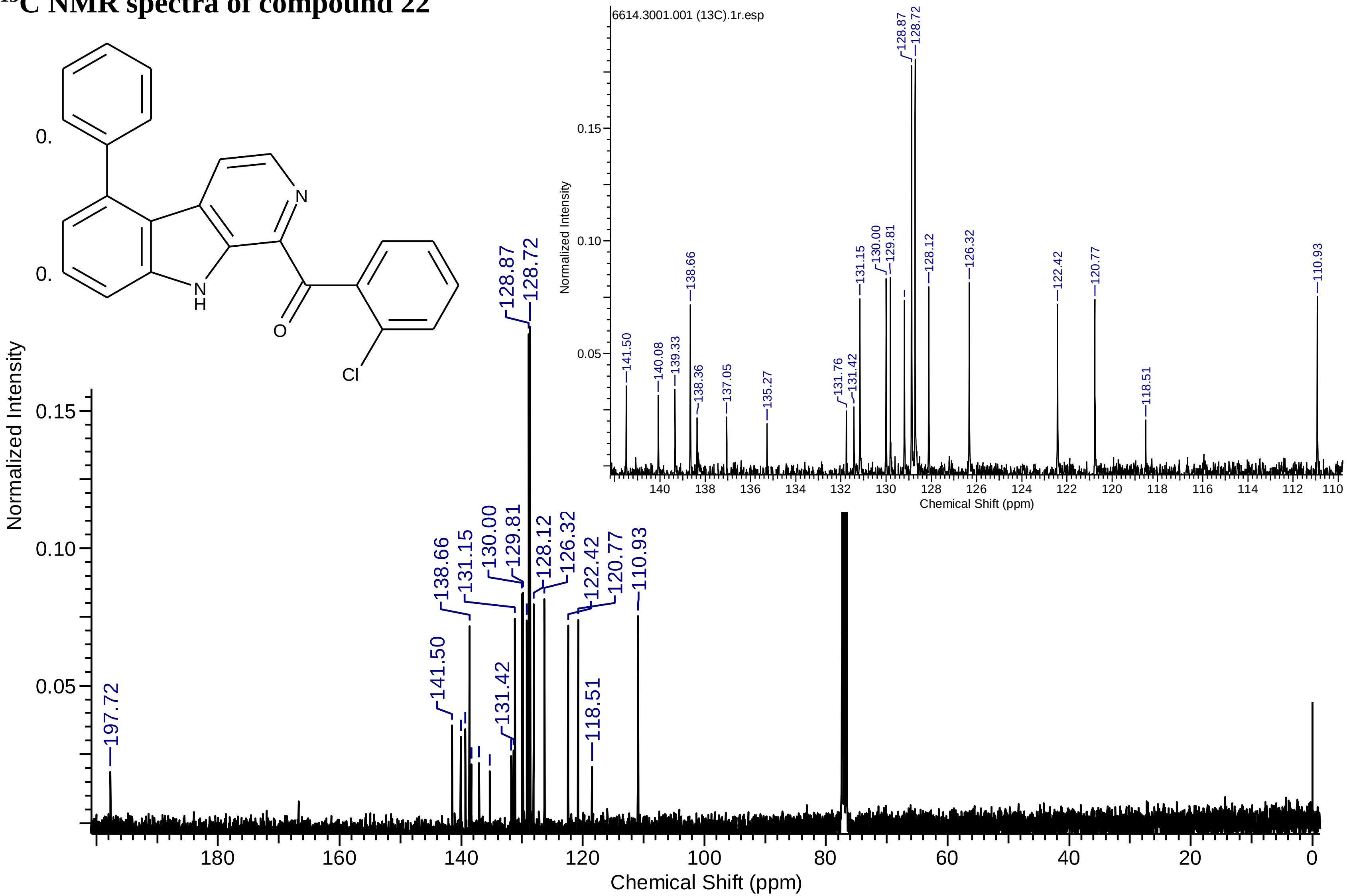

^{1}H NMR spectra of compound 23

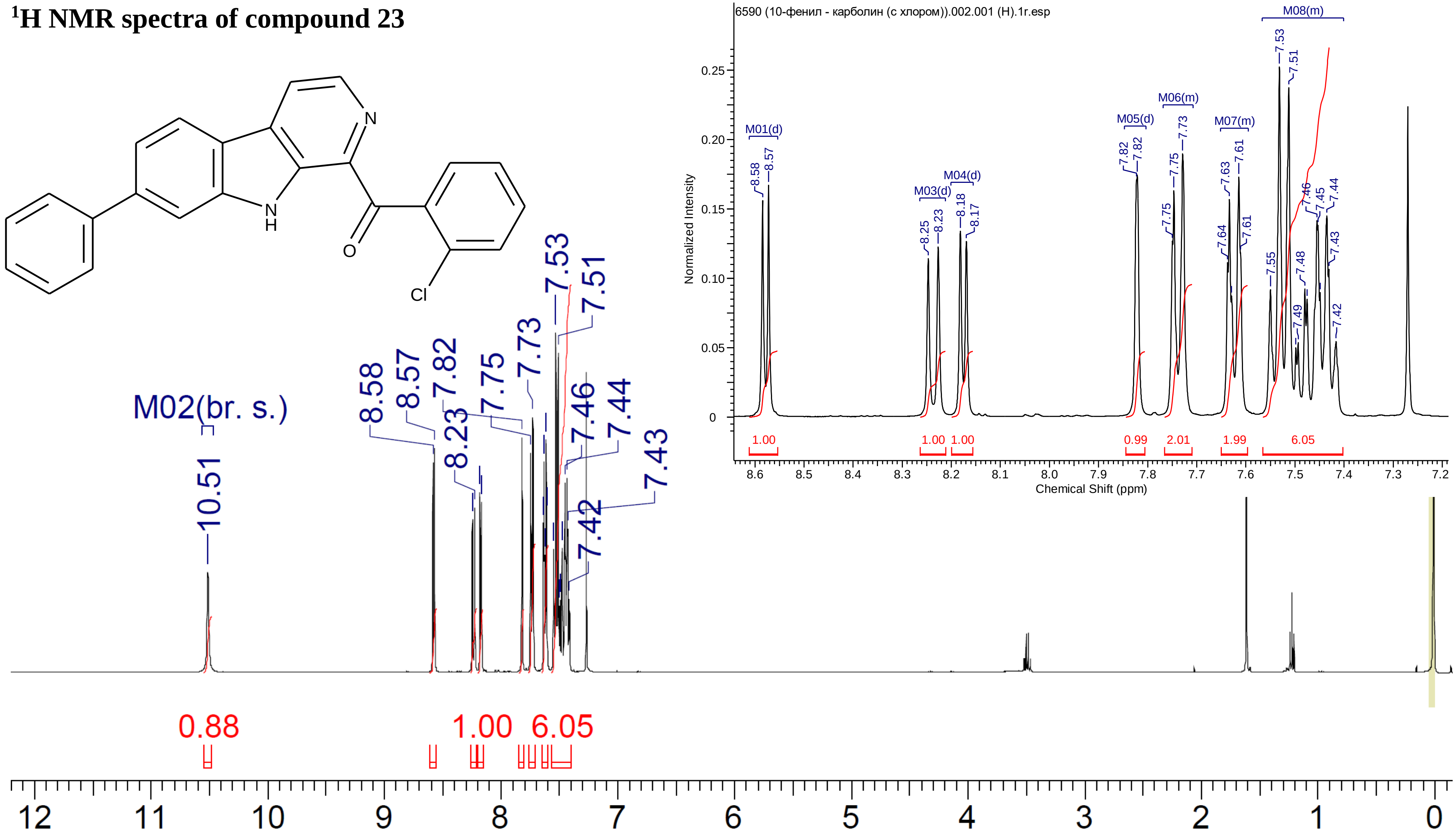

^{13}C NMR spectra of compound 23

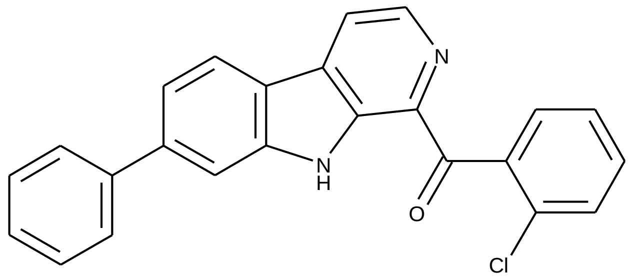

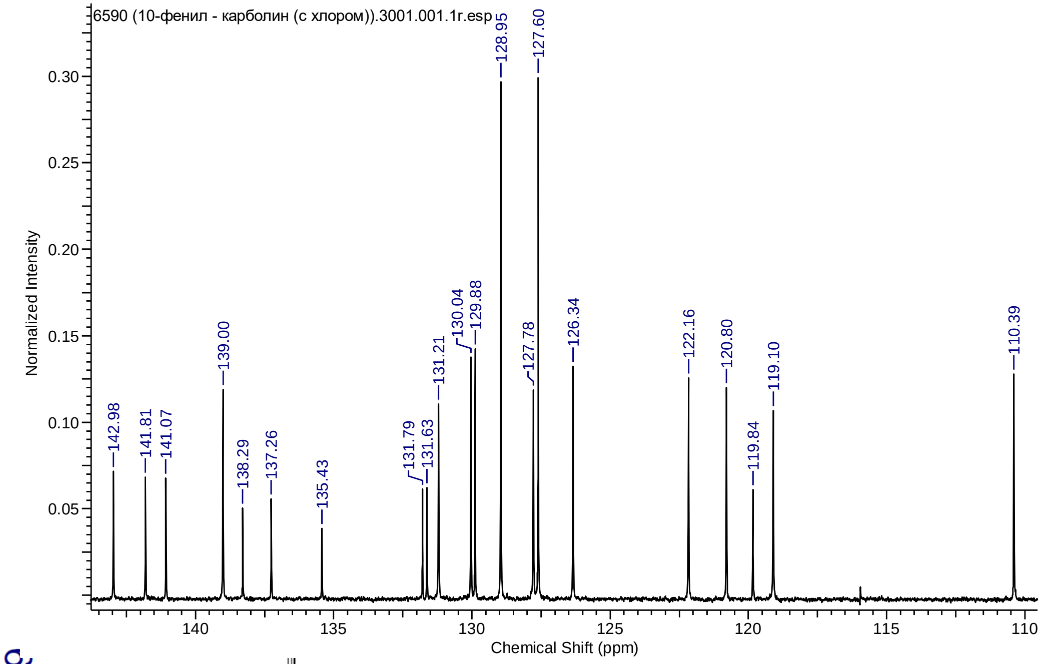

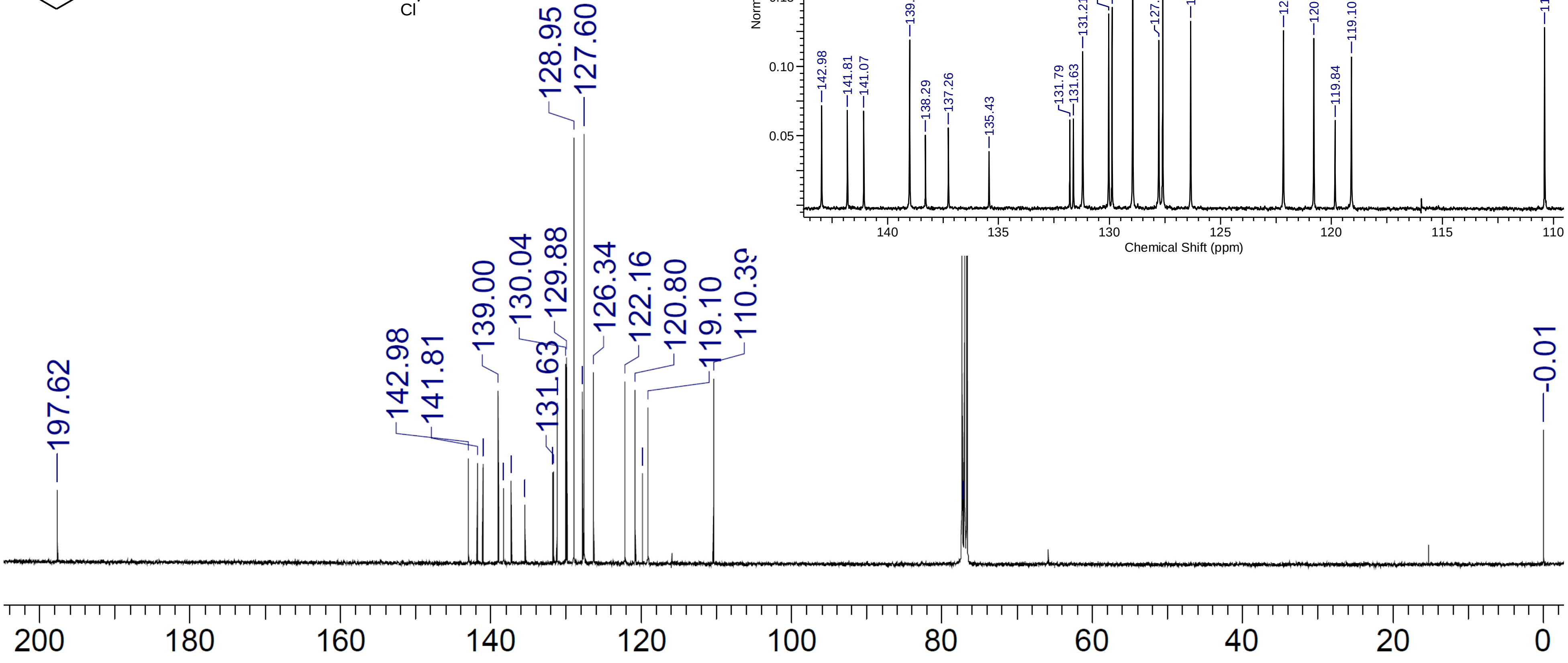

^{1}H NMR spectra of compound 24a

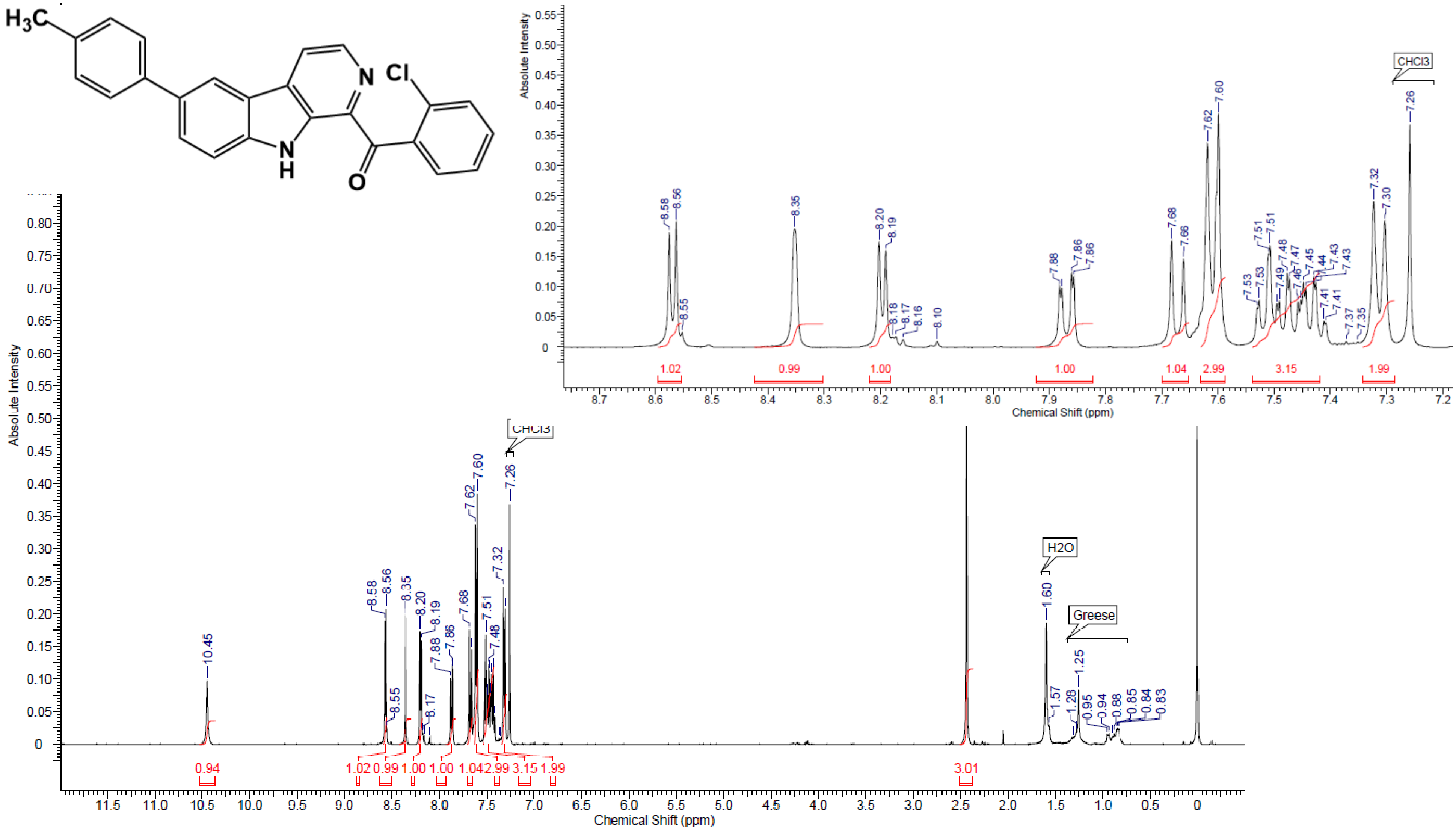

^{13}C NMR spectra of compound 24a

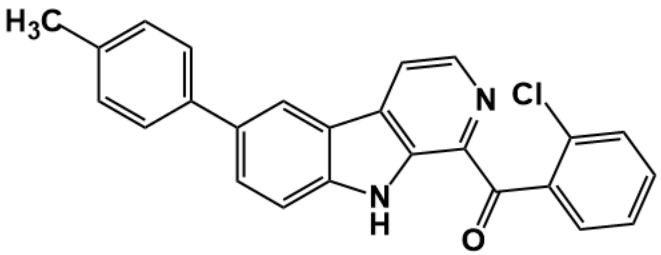

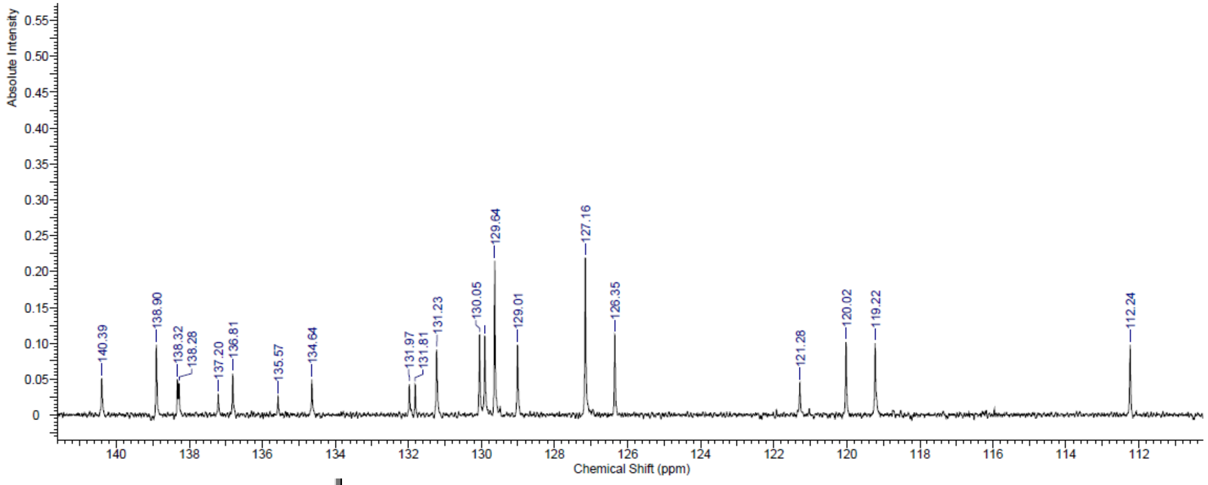

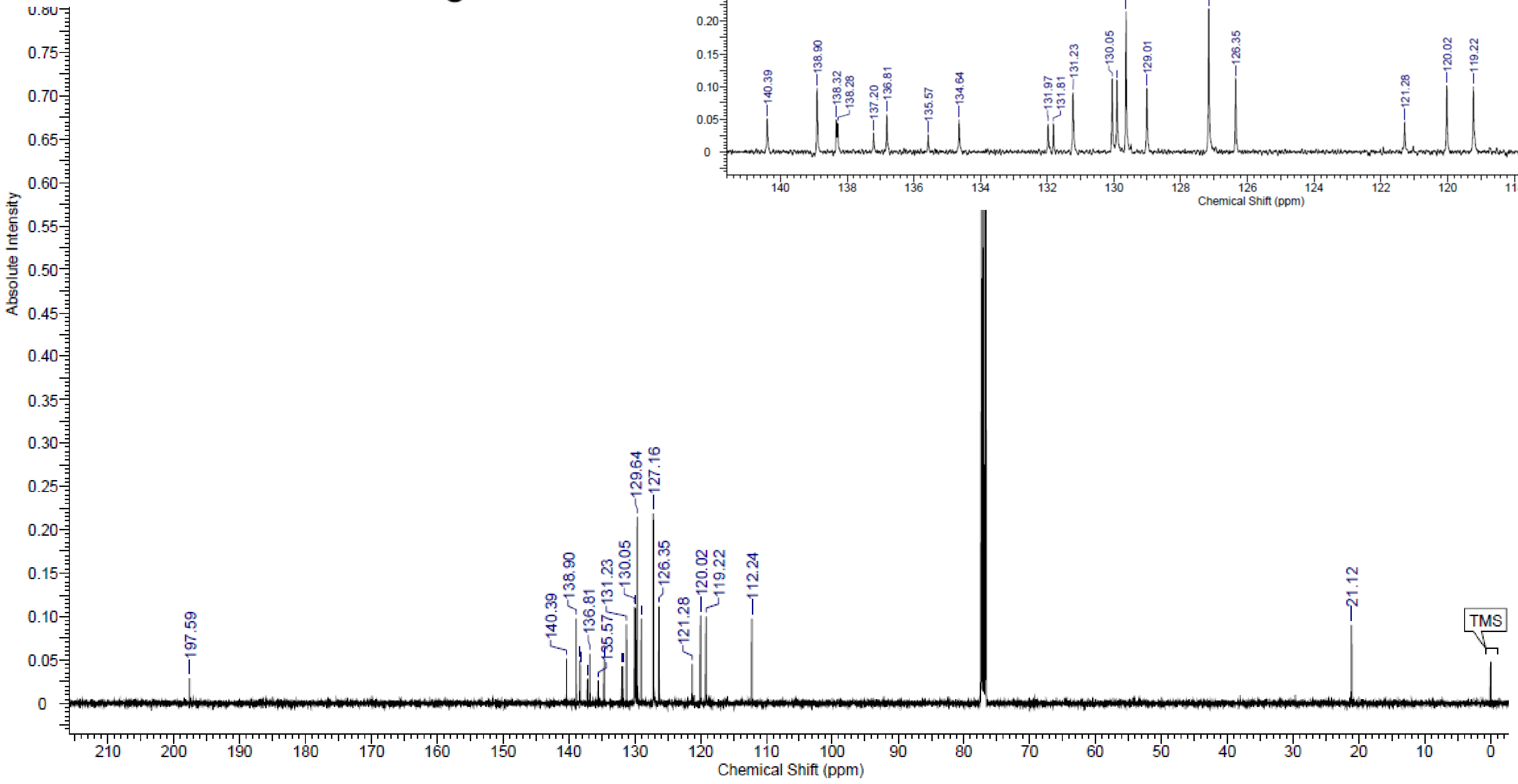

^{1}H NMR spectra of compound 24b

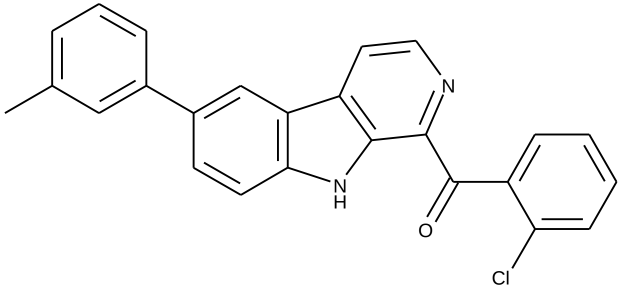

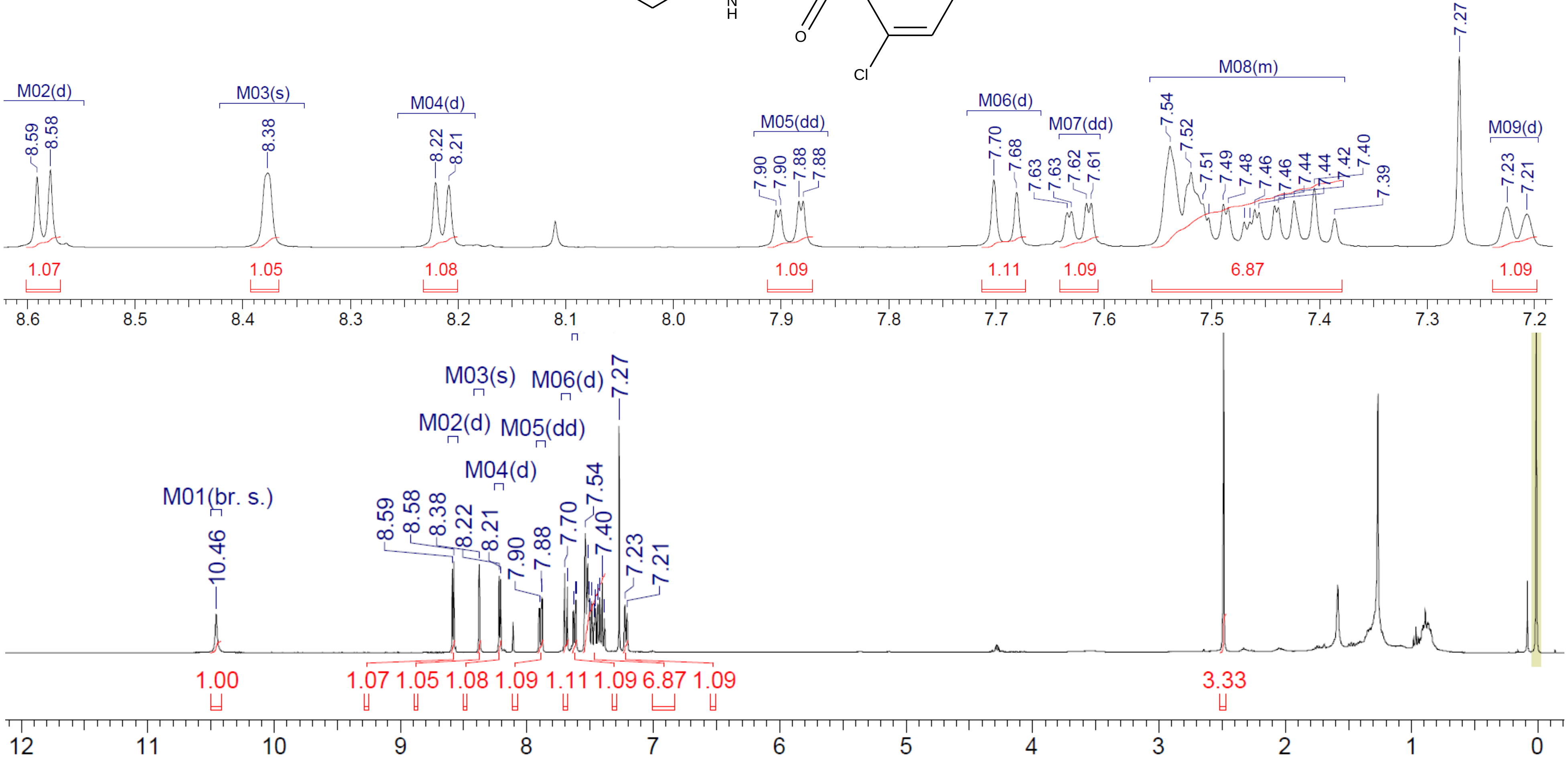

^{13}C NMR spectra of compound 24b

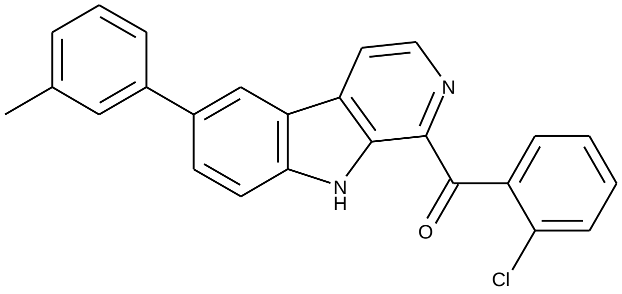

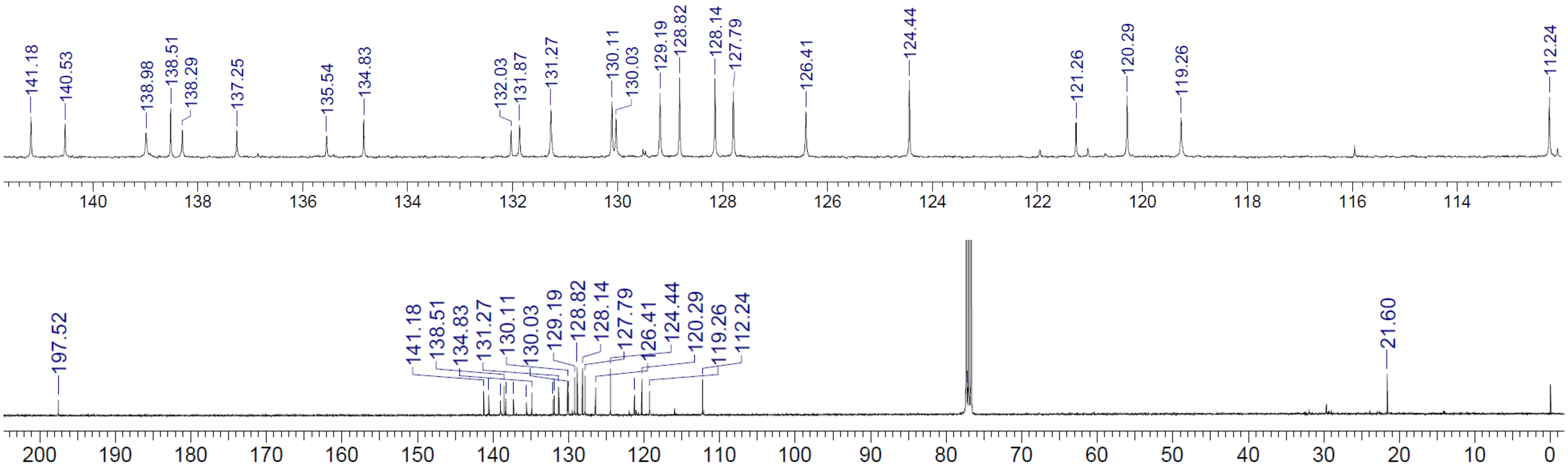

^{1}H NMR spectra of compound 24c

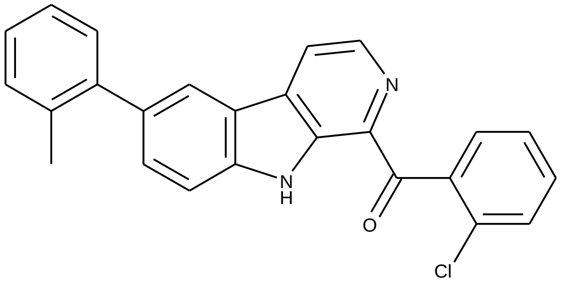

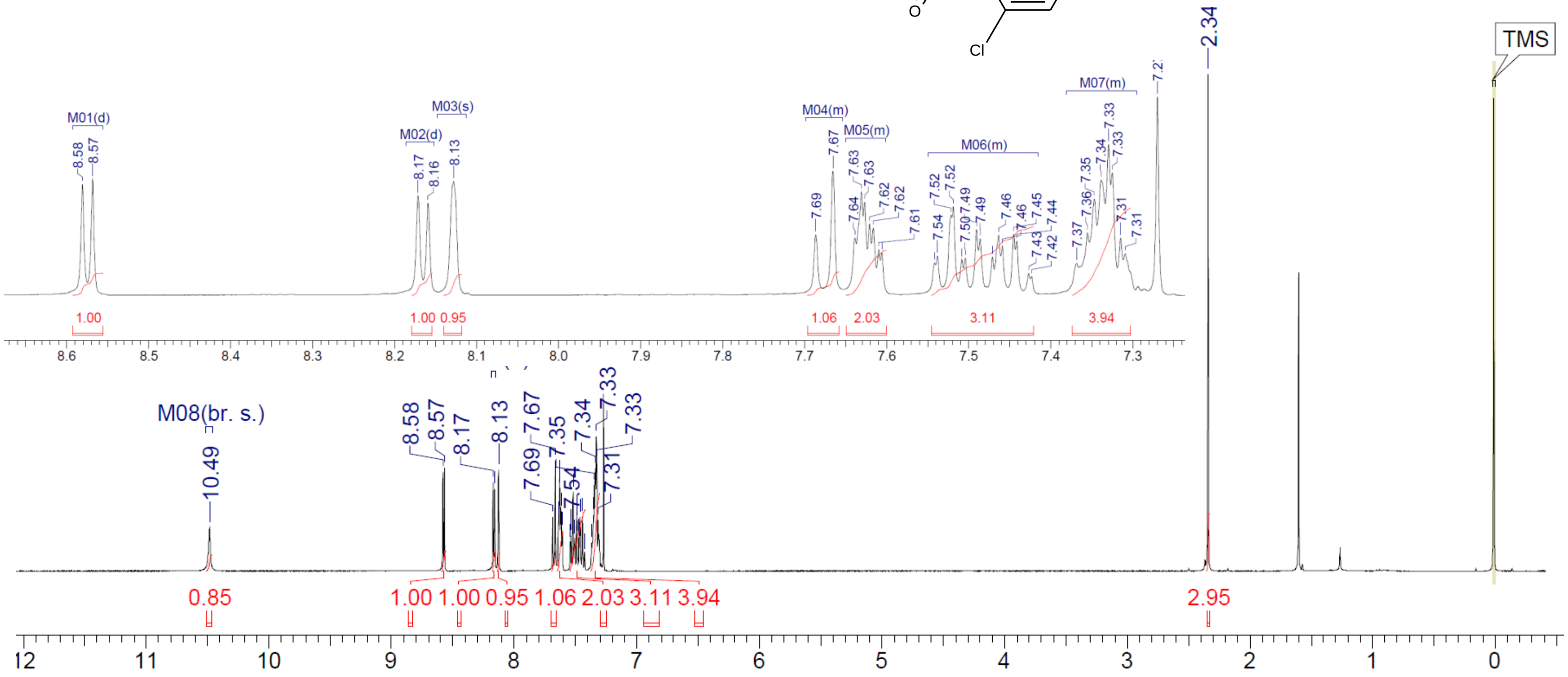

^{13}C NMR spectra of compound 24c

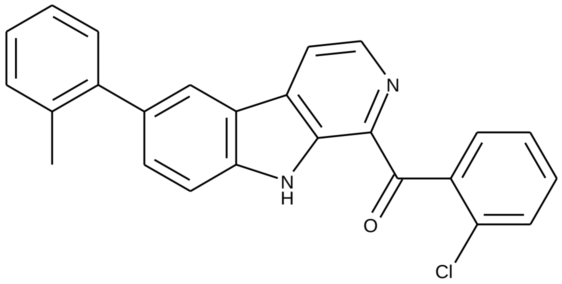

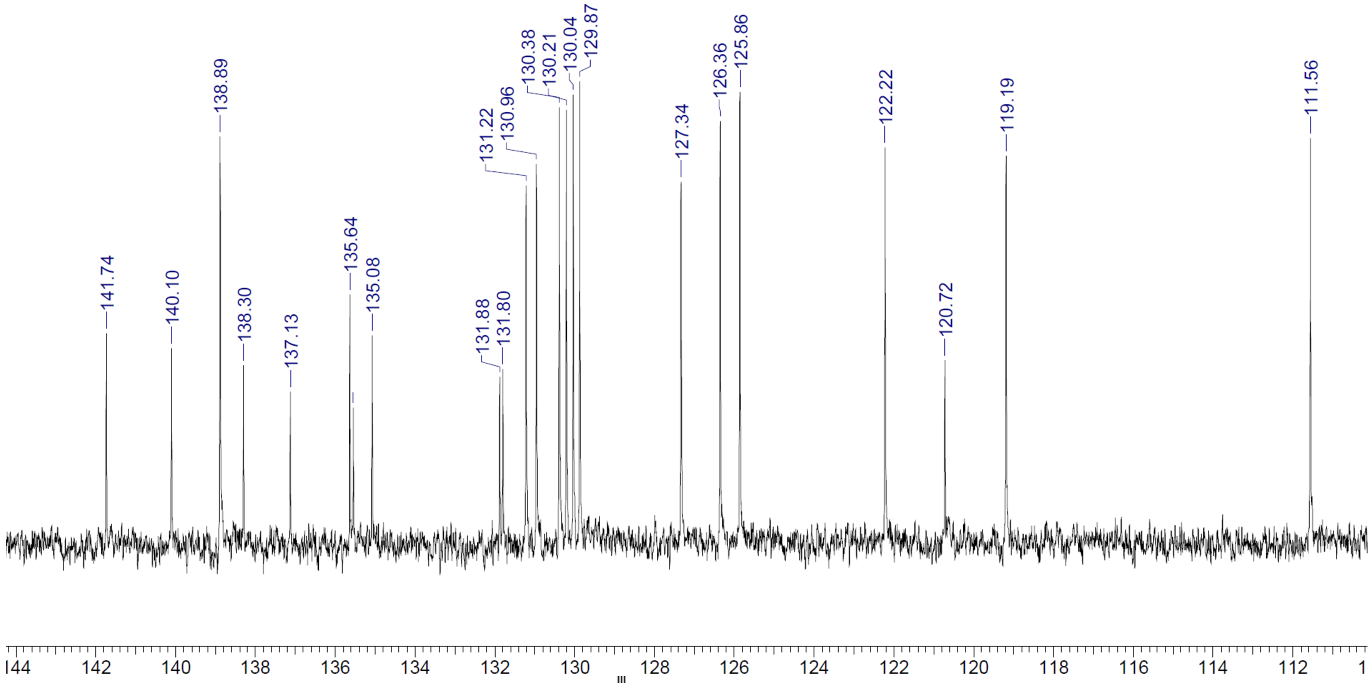

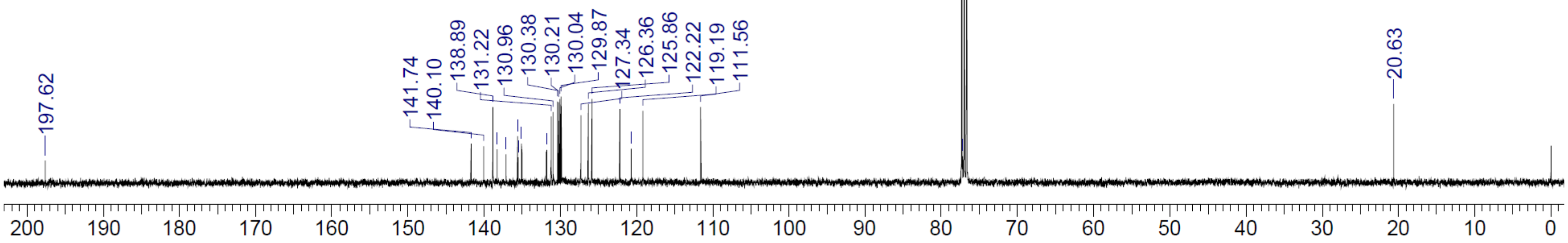

^{1}H NMR spectra of compound 24d

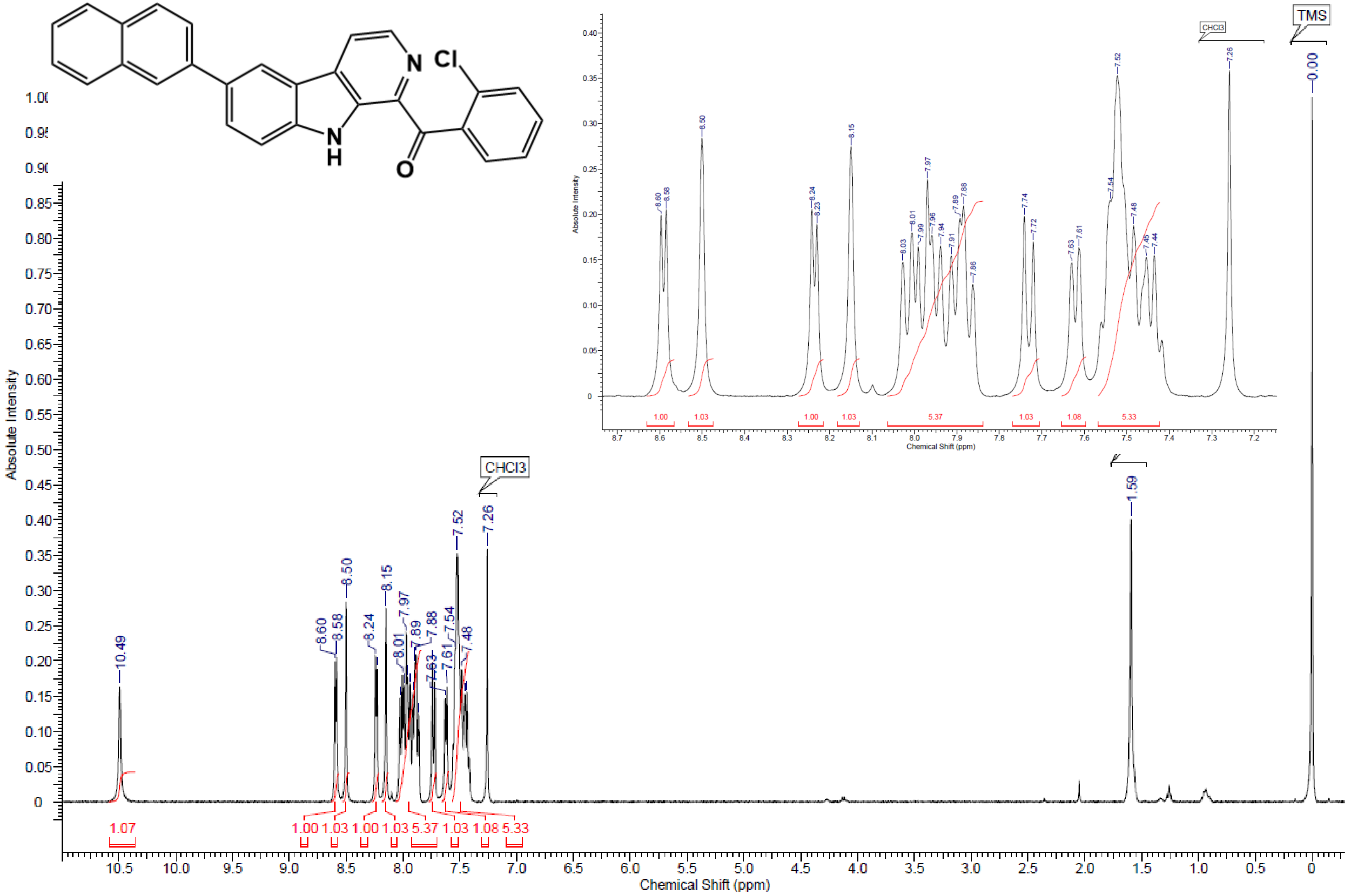

^{13}C NMR spectra of compound 24d

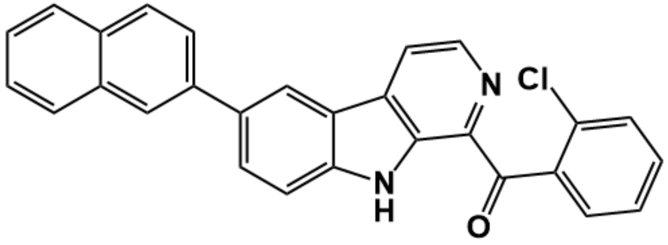

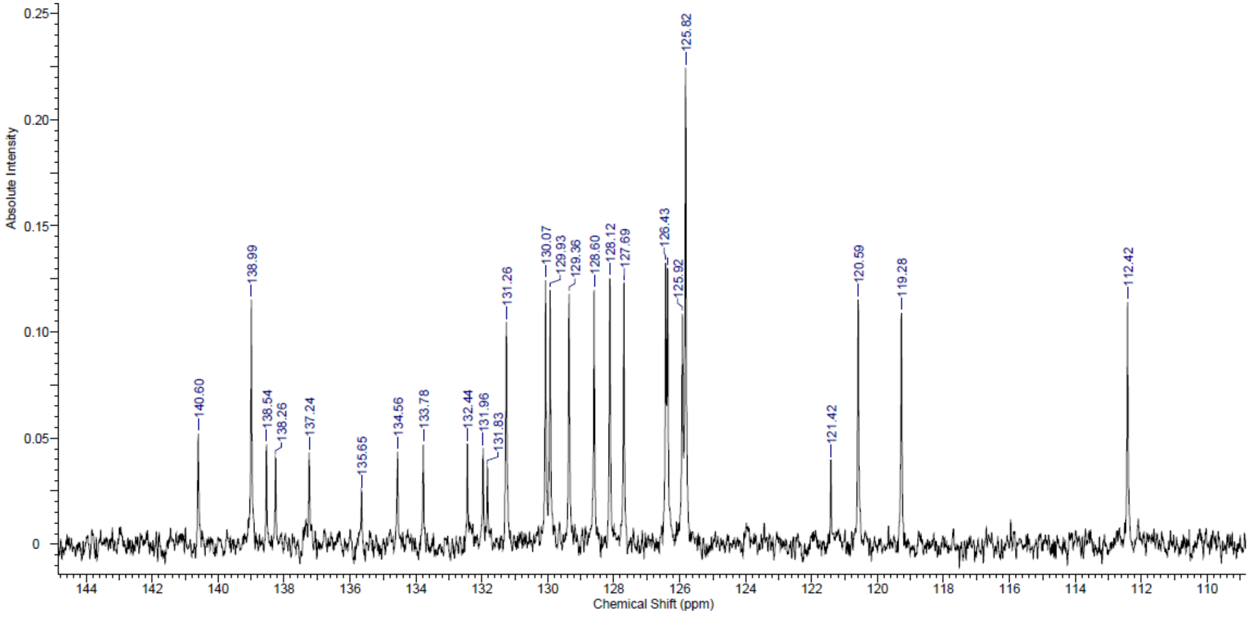

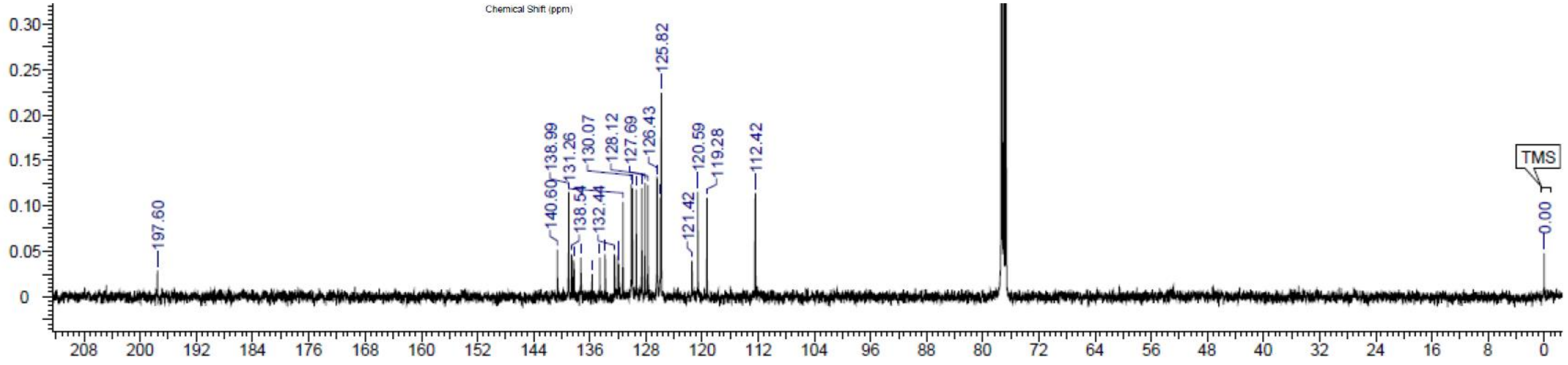

^{1}H NMR spectra of compound 24e

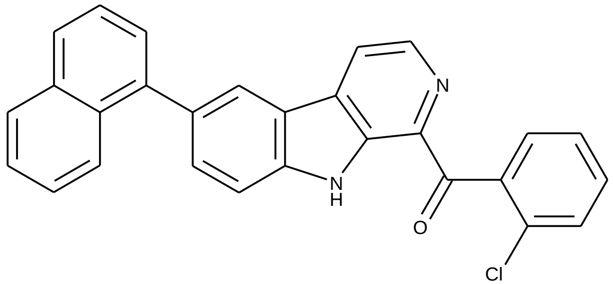

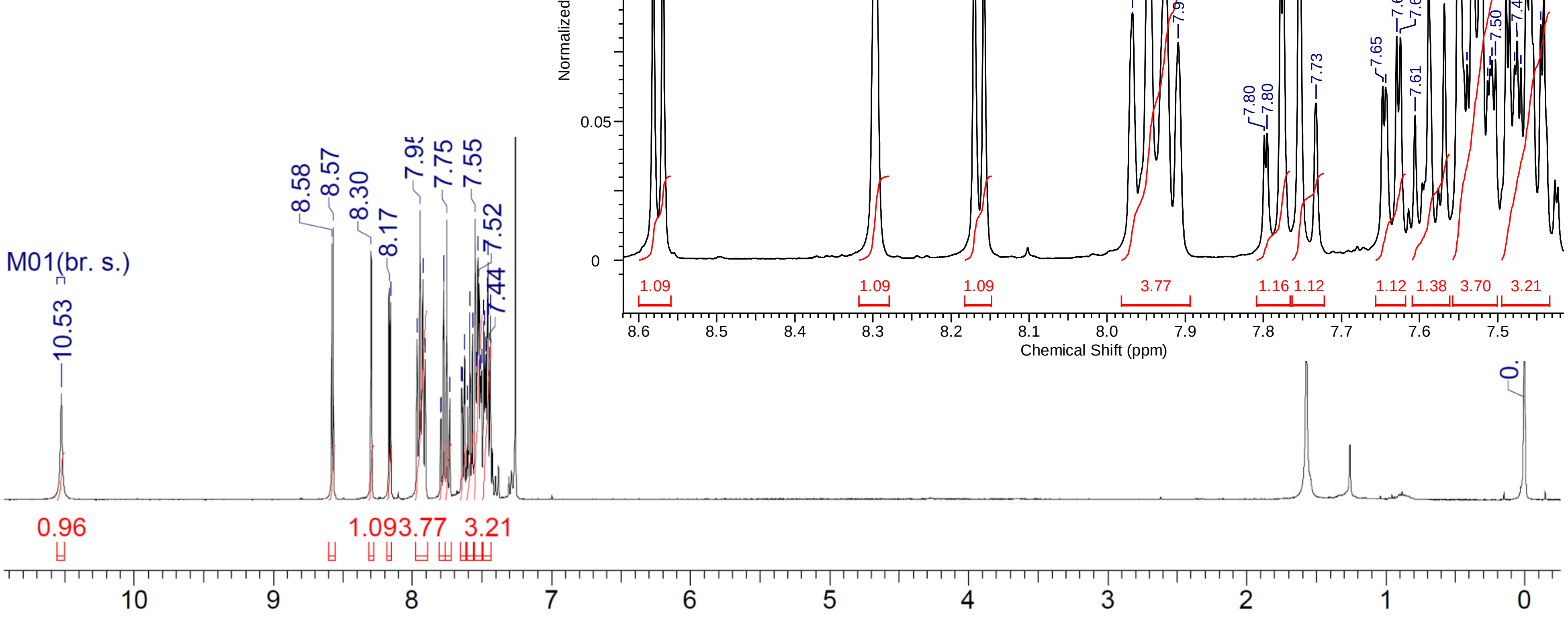

^{13}C NMR spectra of compound 24e

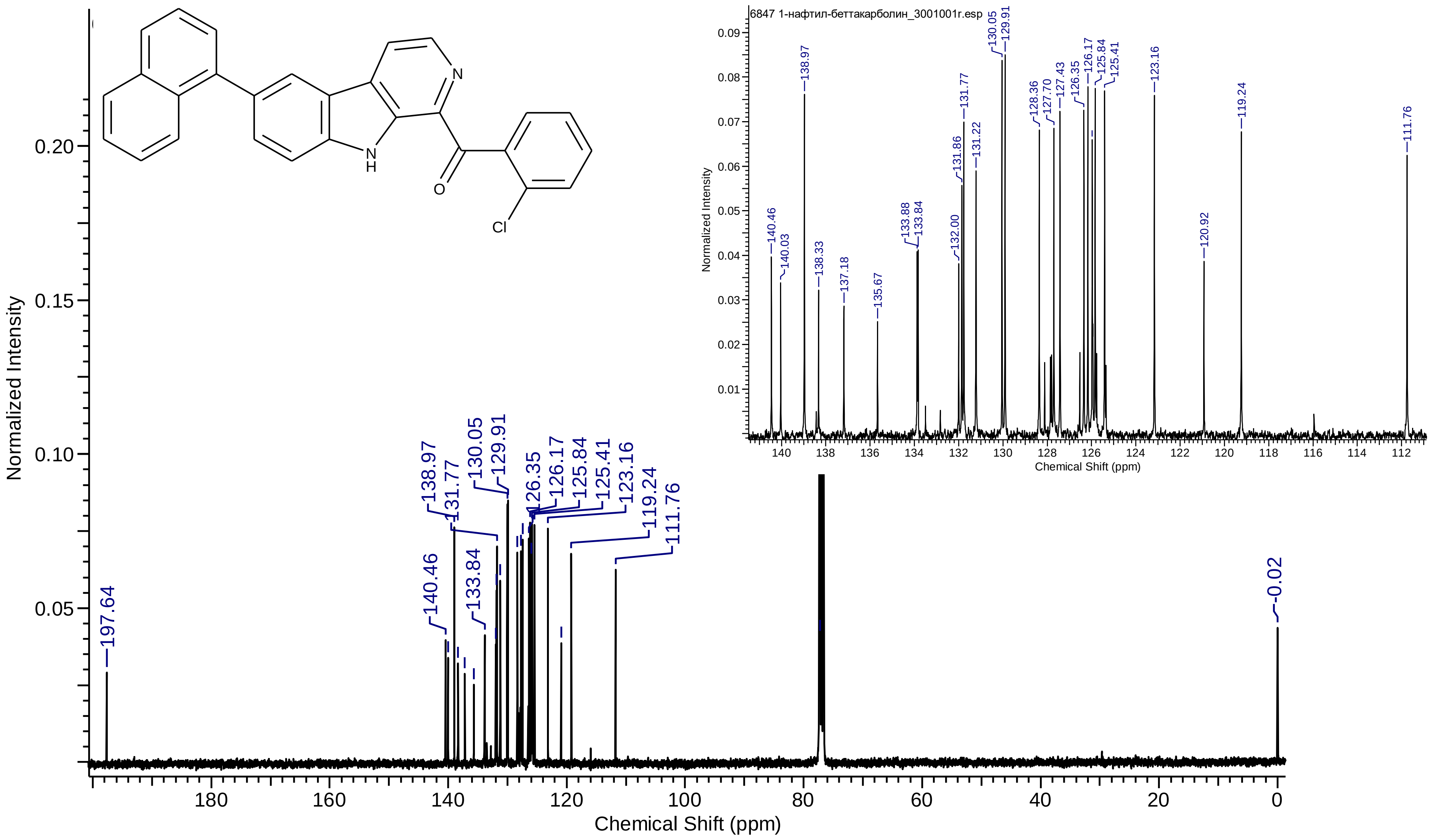

^{1}H NMR spectra of compound 24f

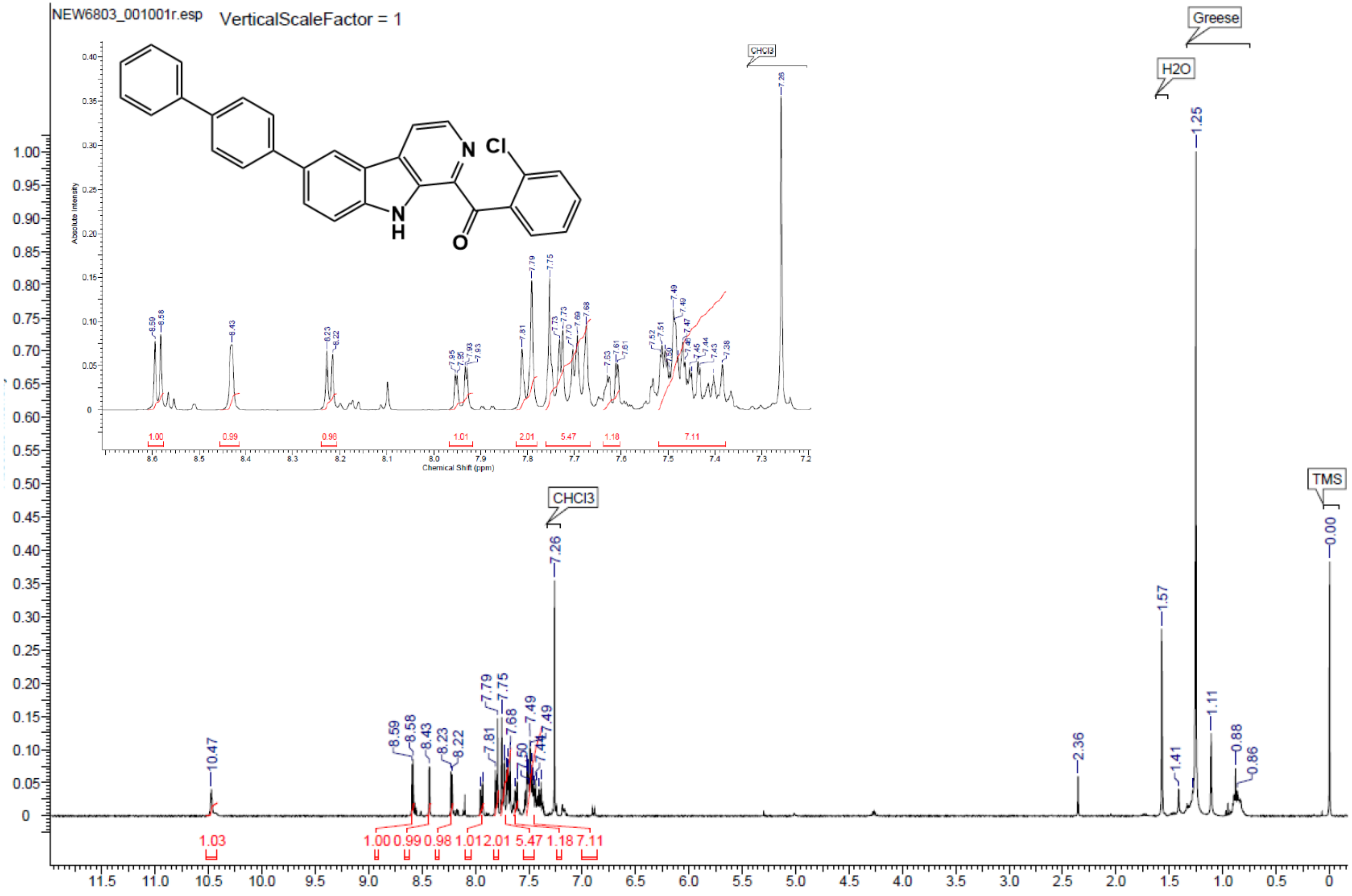

^{13}C NMR spectra of compound 24f

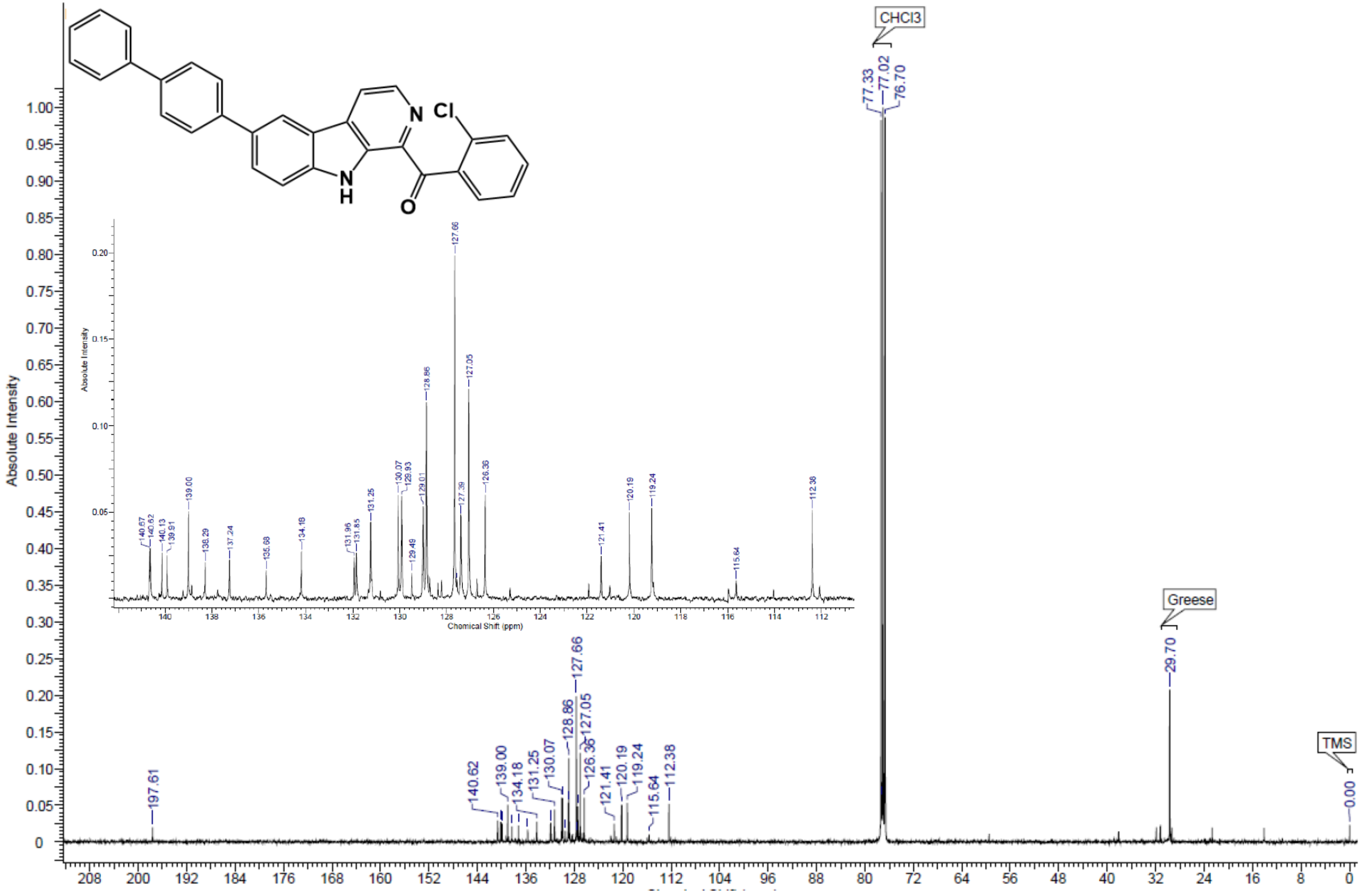

^{1}H NMR spectra of compound 24g

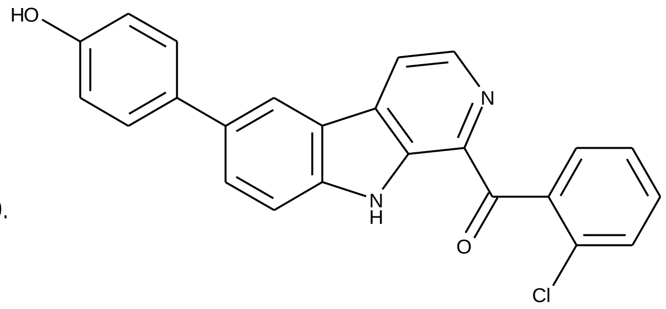

0.

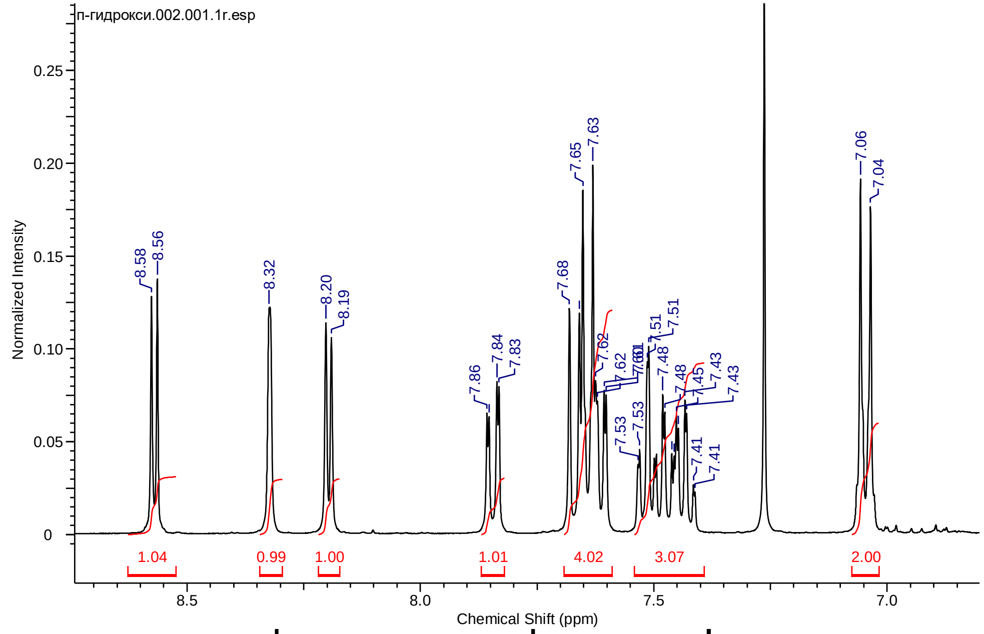
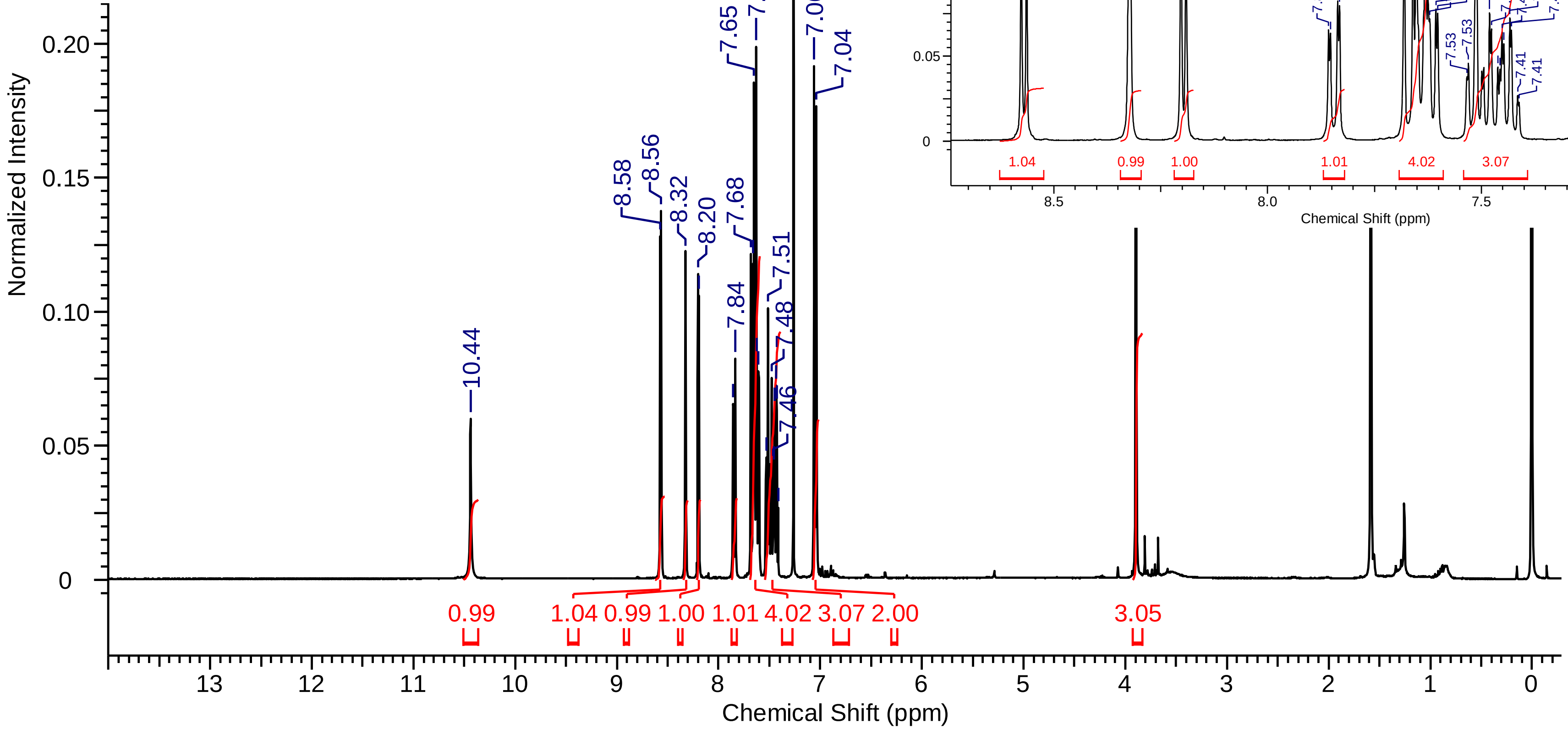

^{13}C NMR spectra of compound 24g

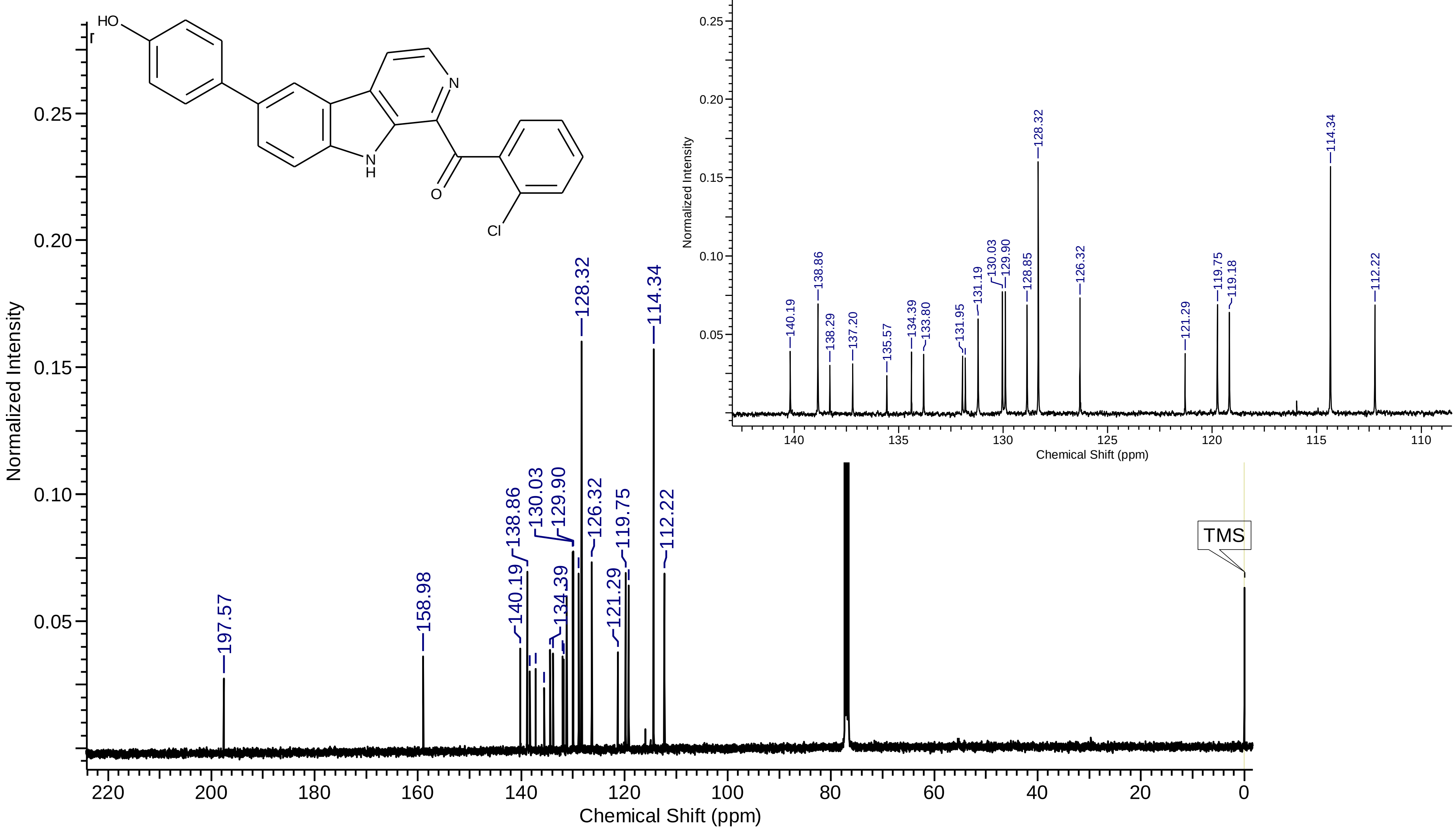

^{1}H NMR spectra of compound 24h

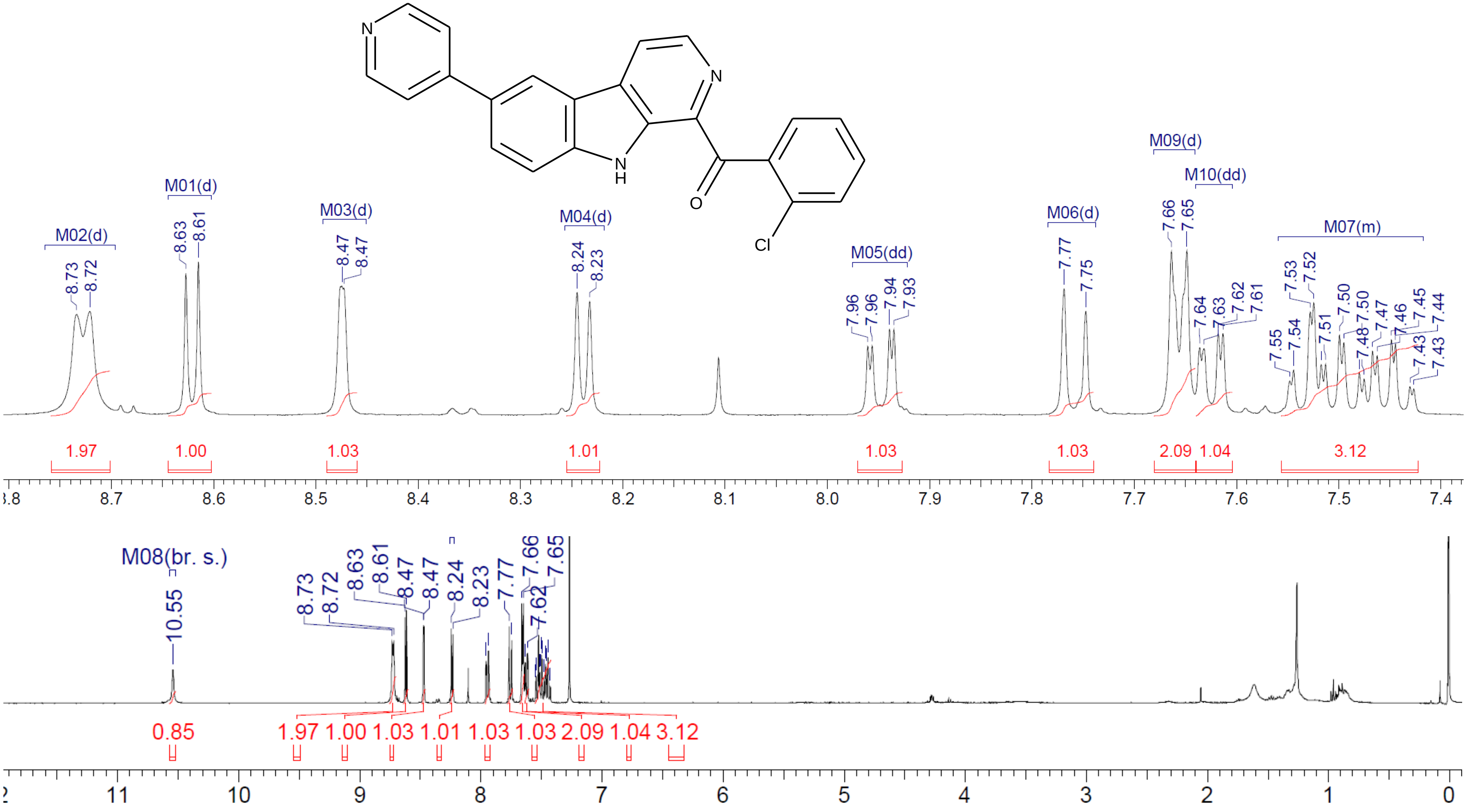

^{13}C NMR spectra of compound 24h

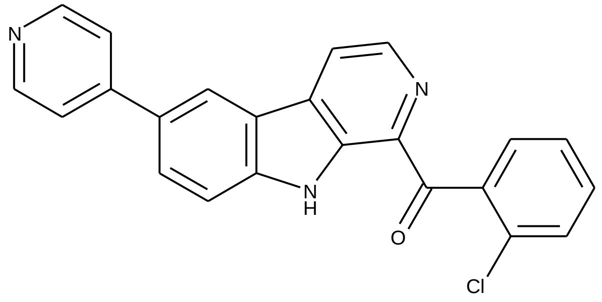

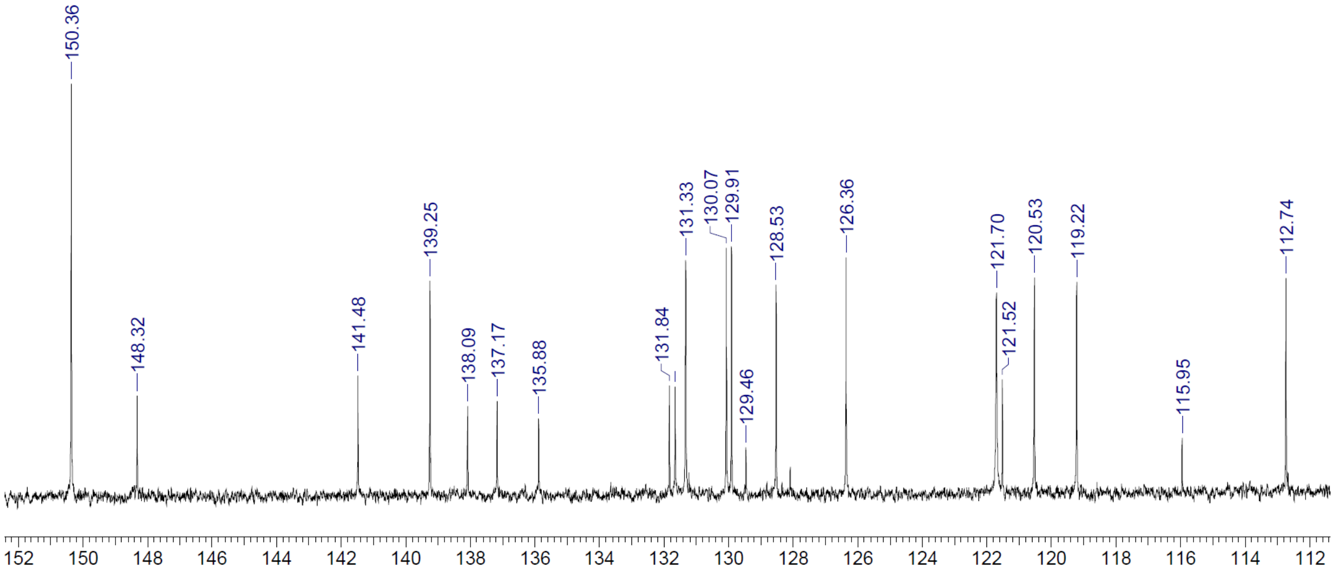

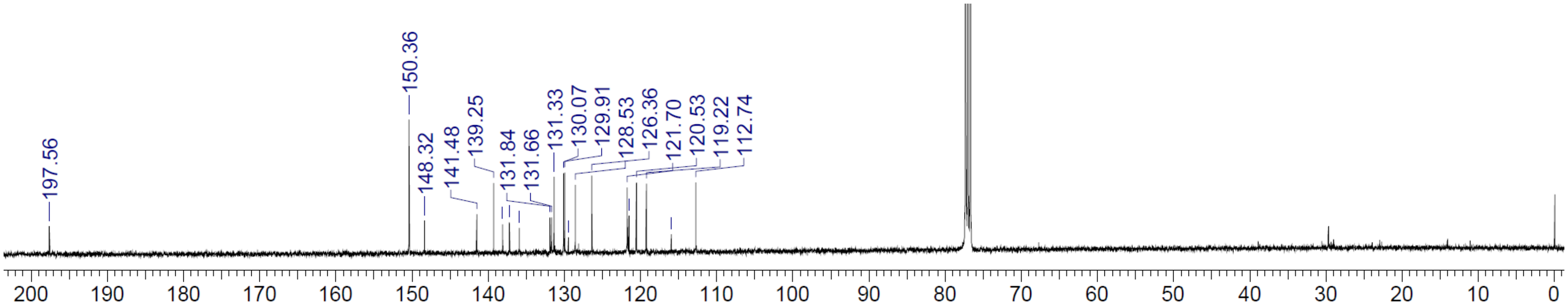

^{1}H NMR spectra of compound 15

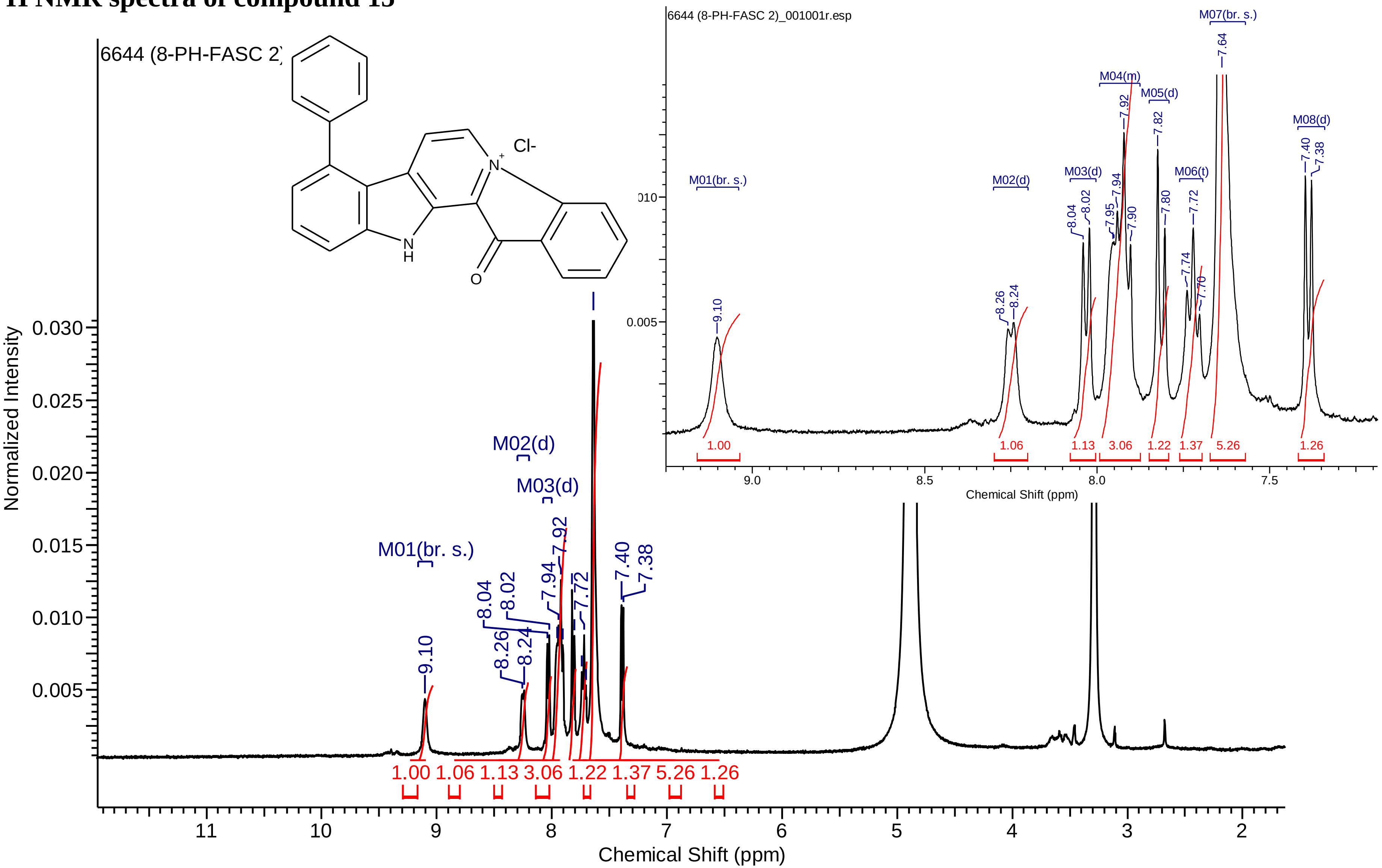

^{13}C NMR spectra of compound 15

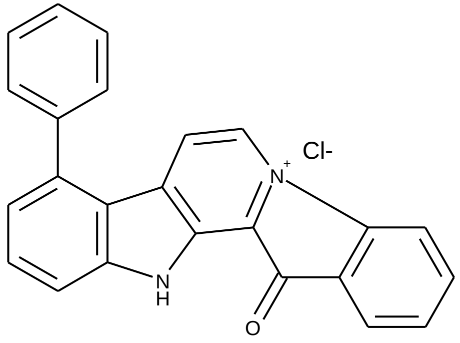

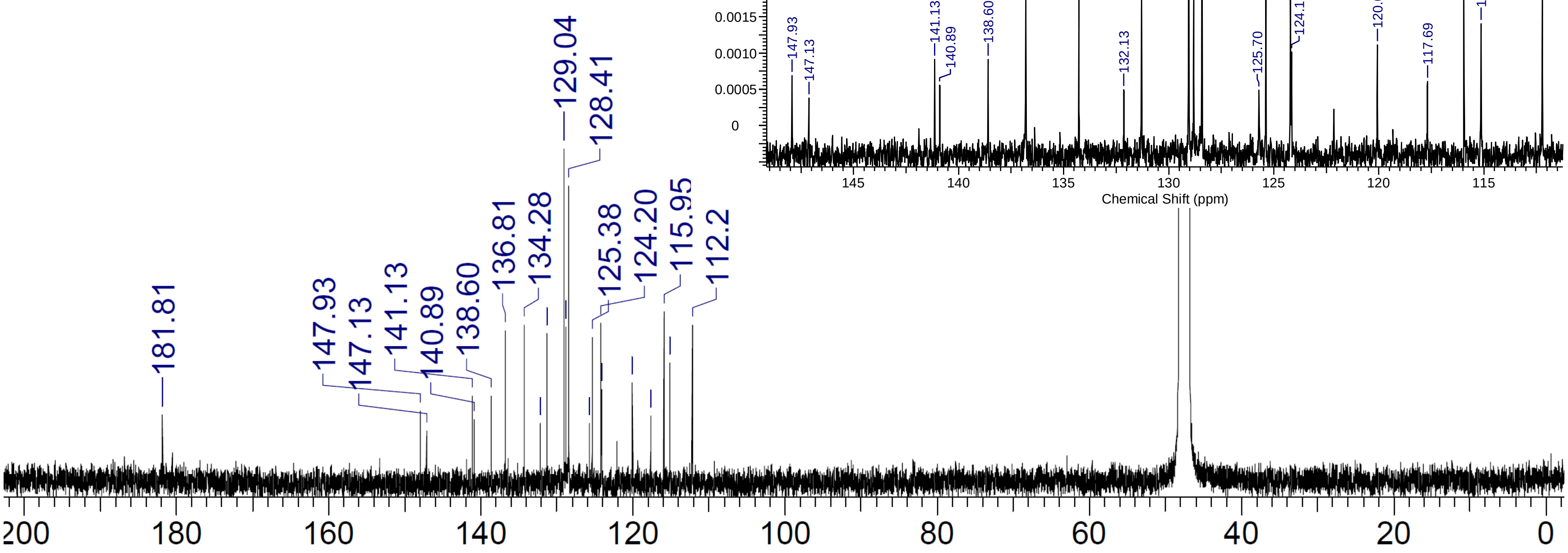

^{1}H NMR spectra of compound 16

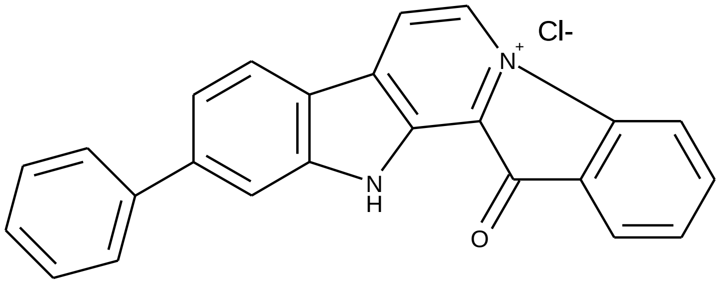

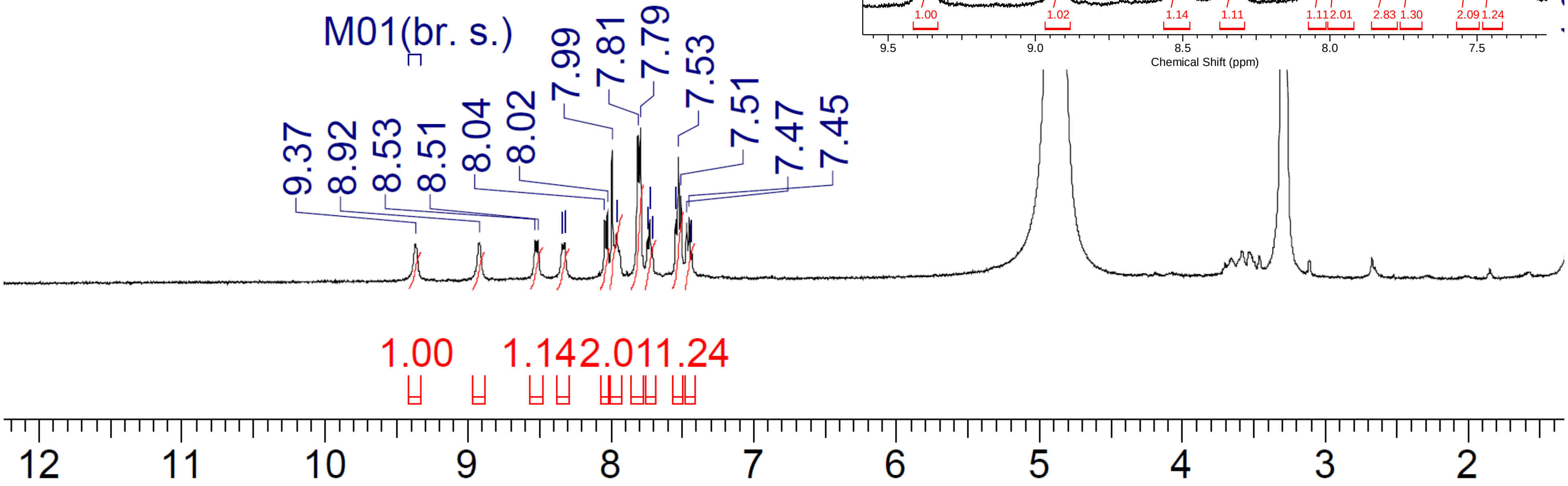

^{13}C NMR spectra of compound 16

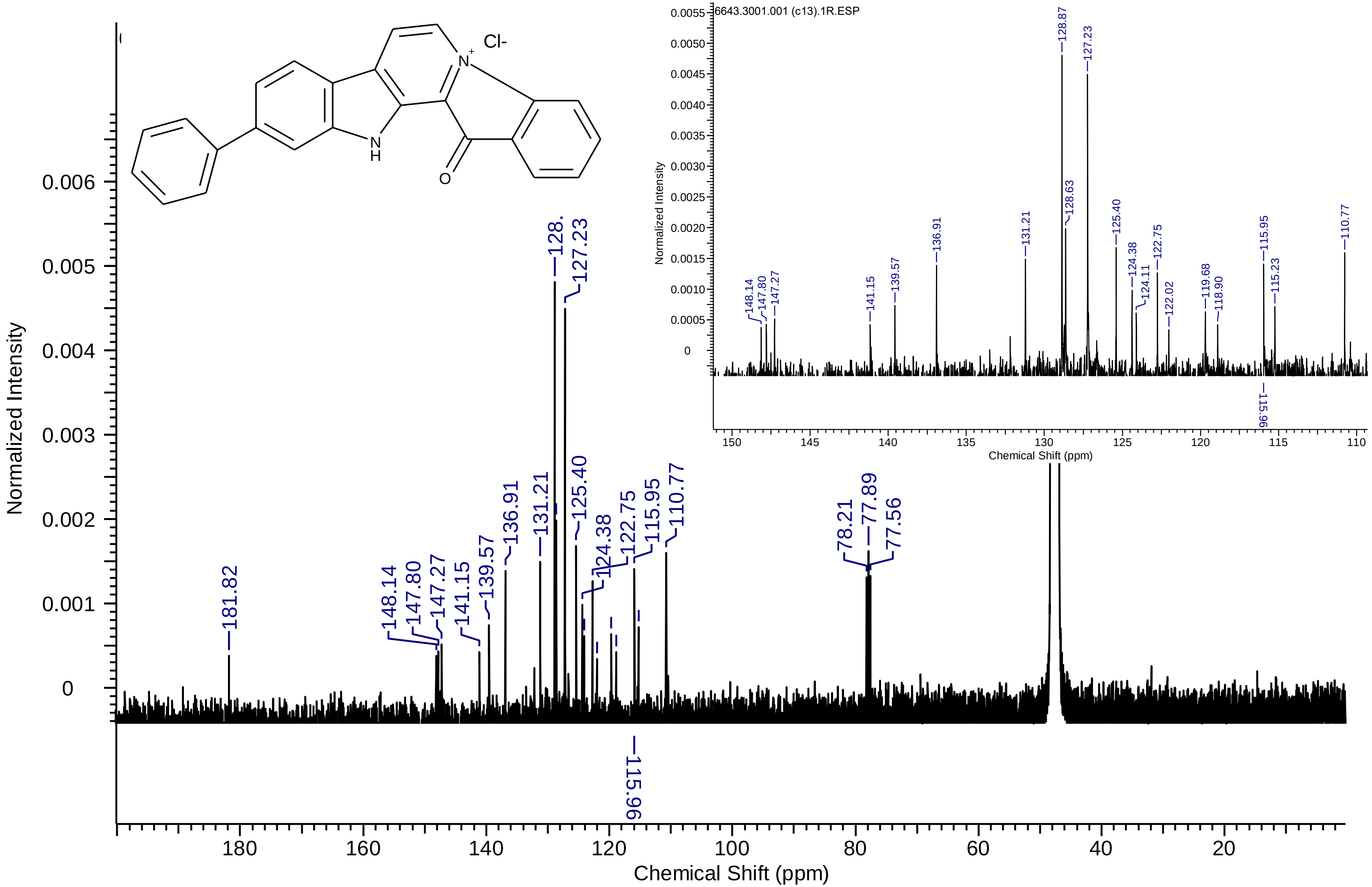

^{1}H NMR spectra of compound 25a

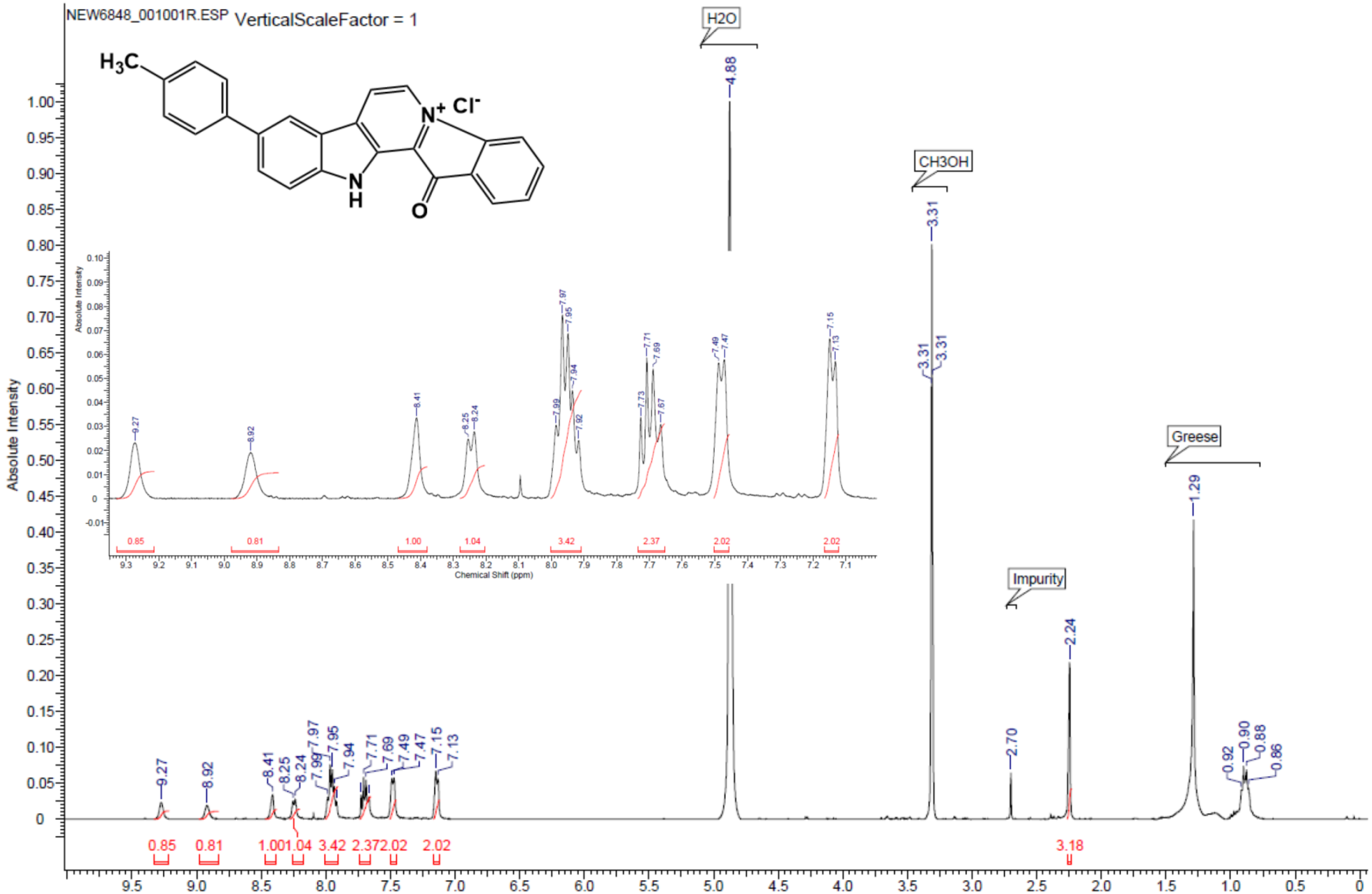

^{13}C NMR spectra of compound 25a

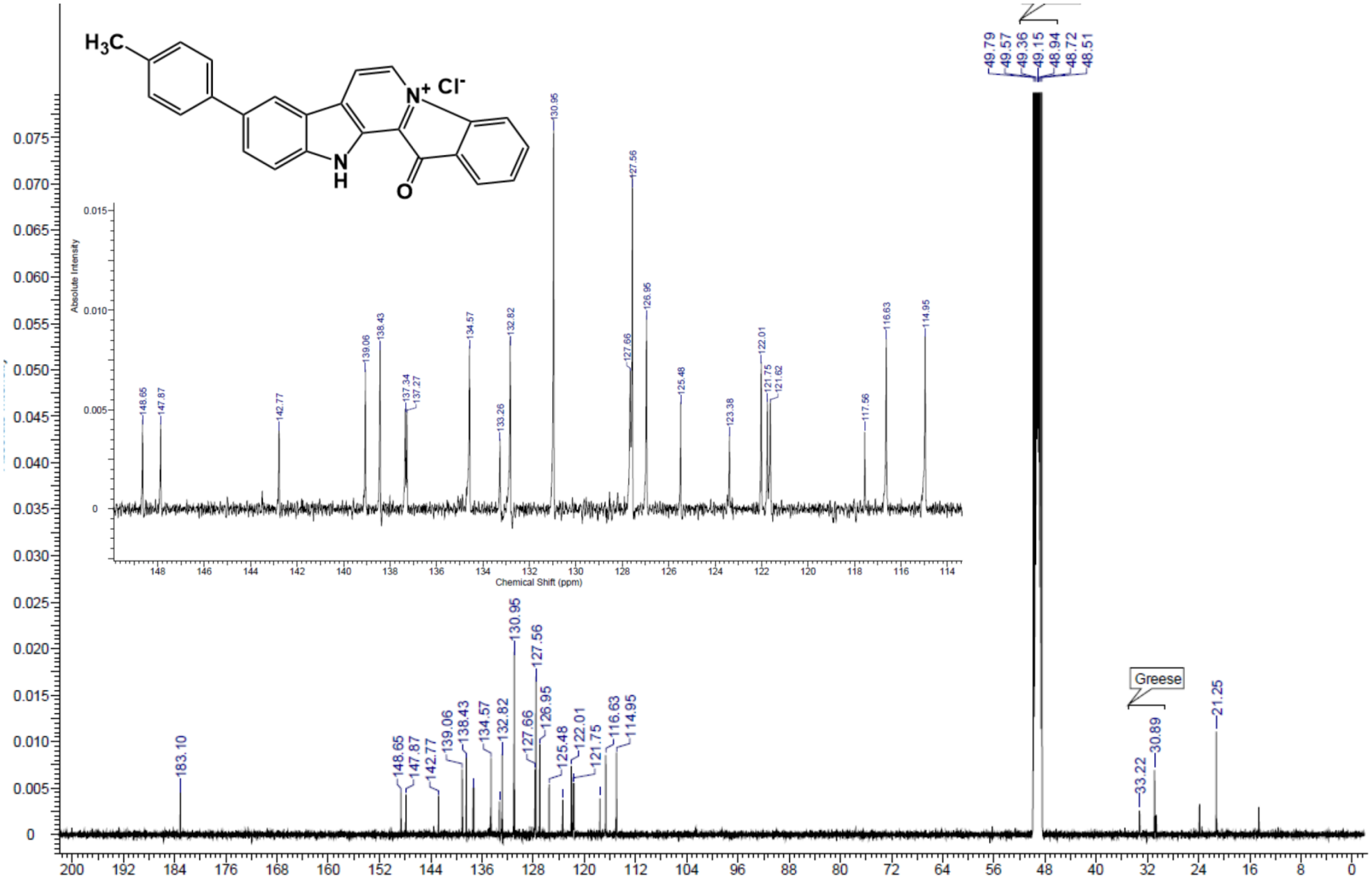

^{1}H NMR spectra of compound 25b

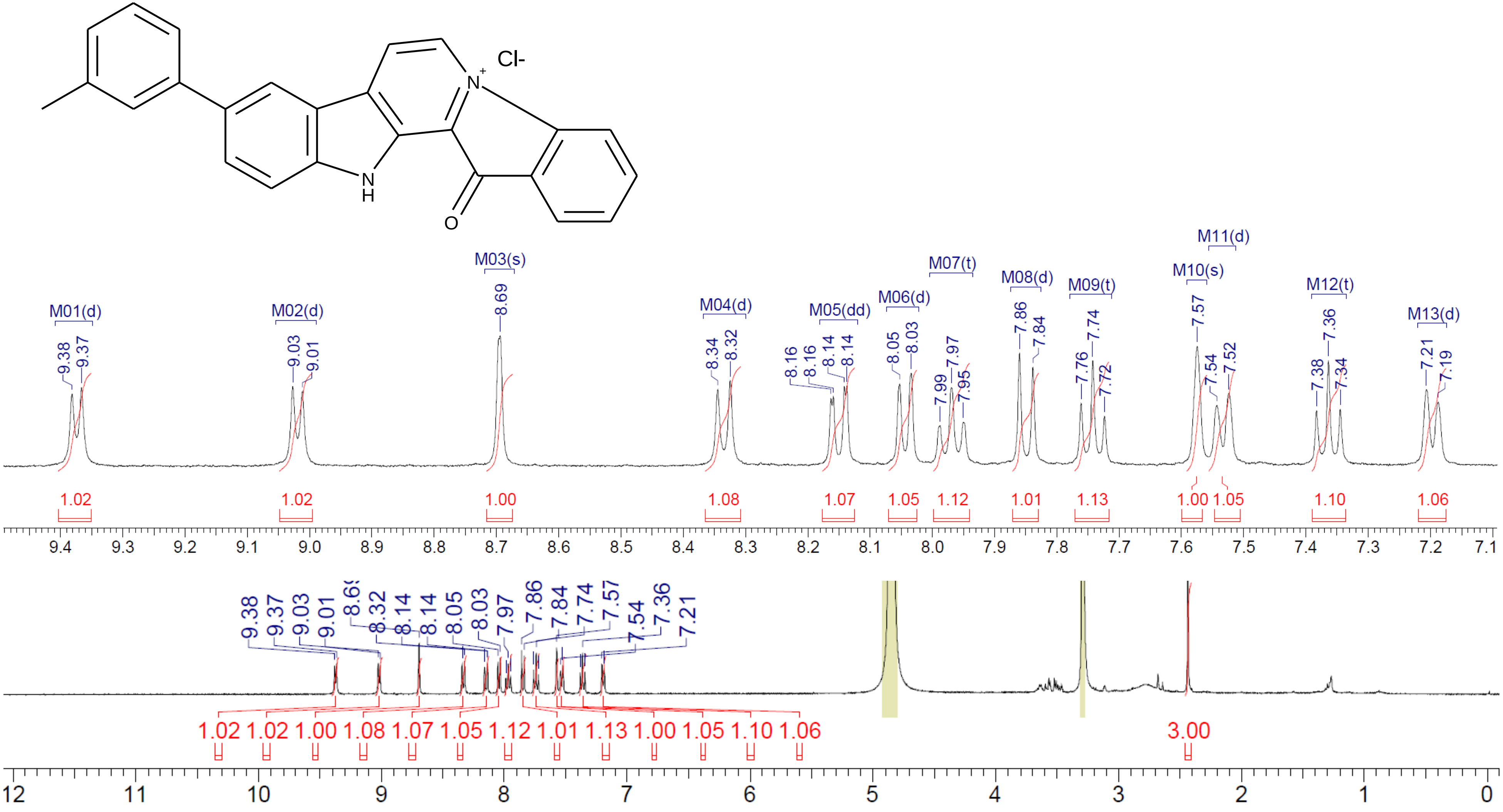

^{13}C NMR spectra of compound 25b

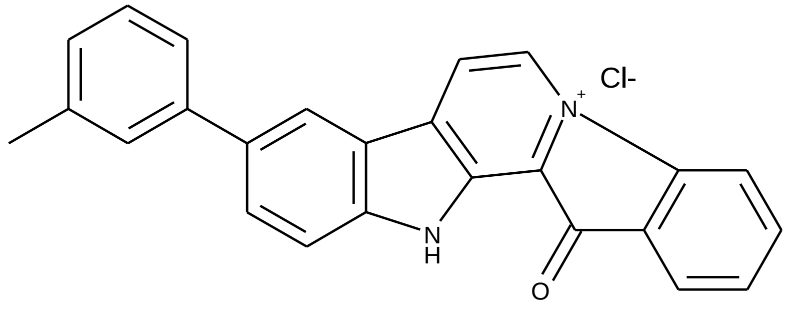

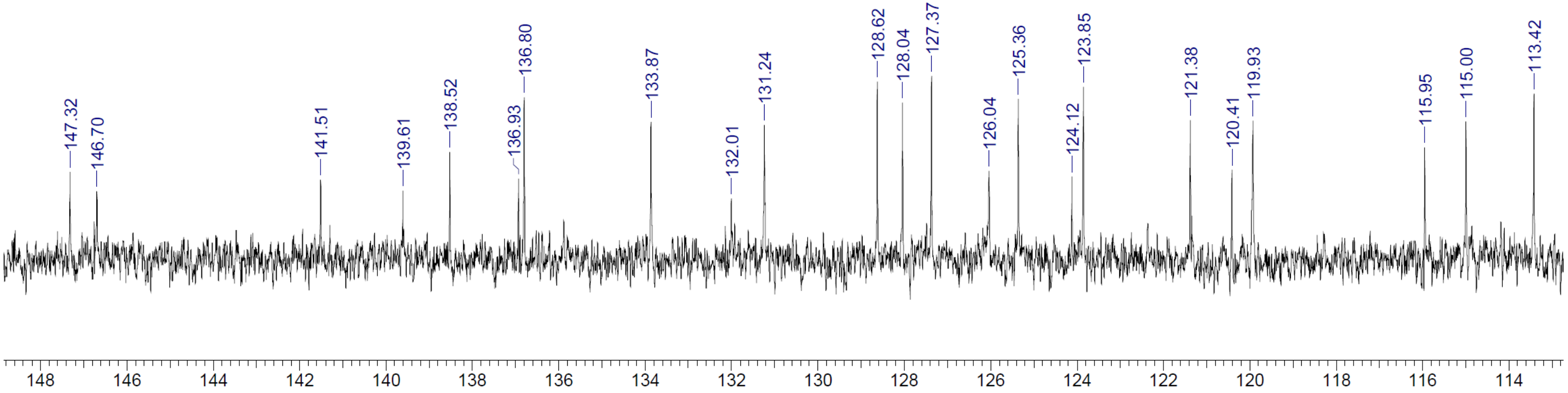

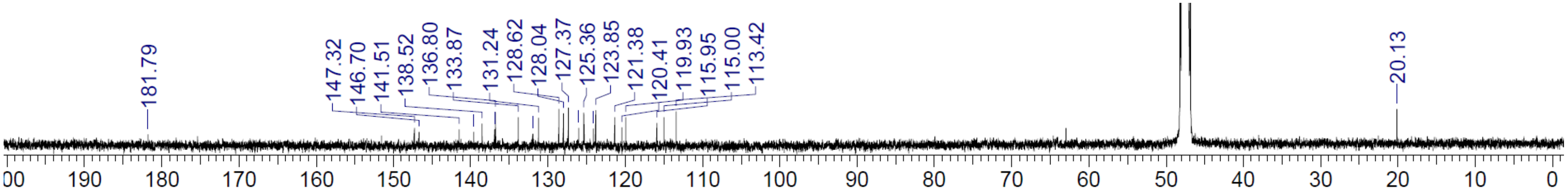

^{1}H NMR spectra of compound 25c

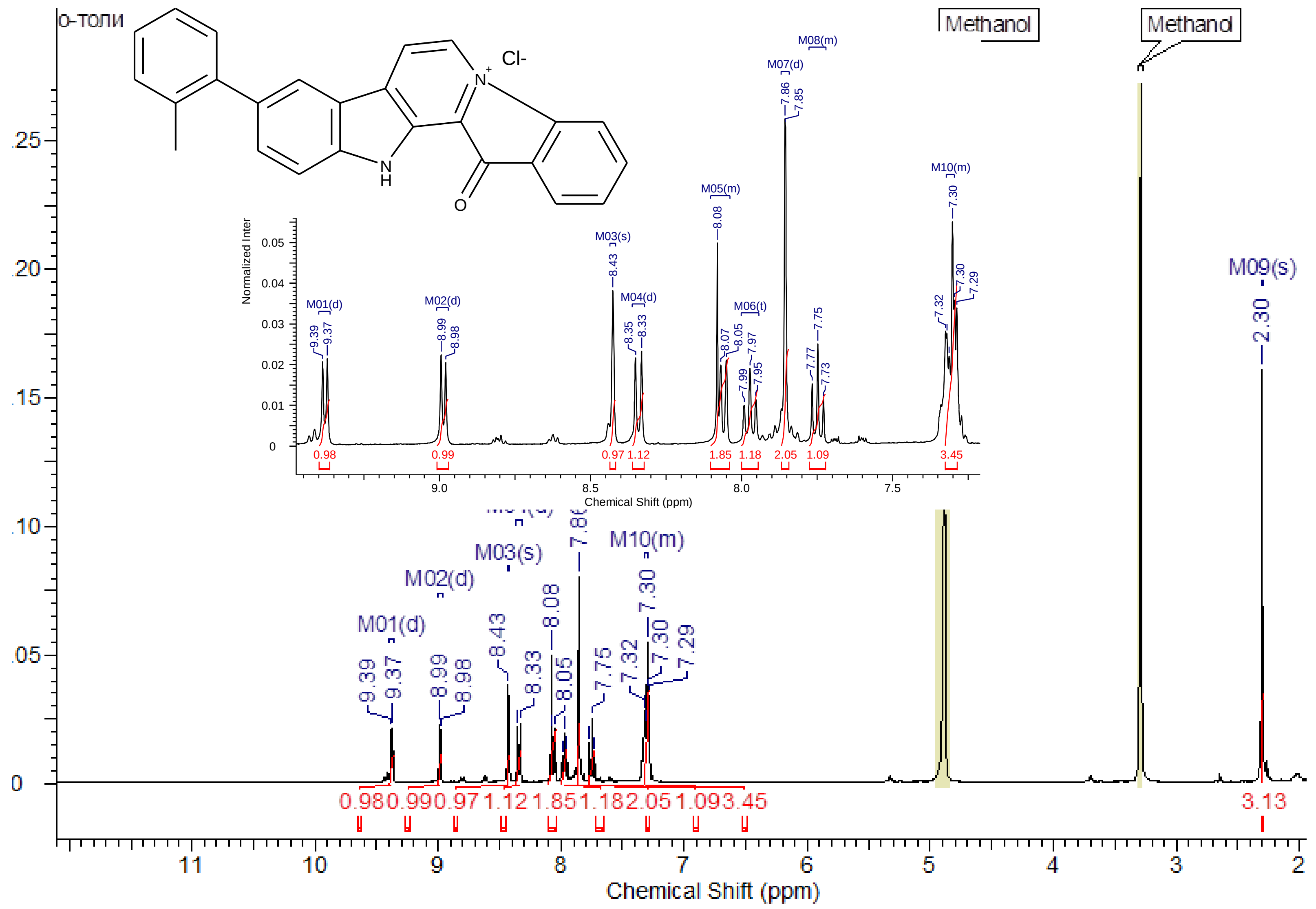

^{13}C NMR spectra of compound 25c

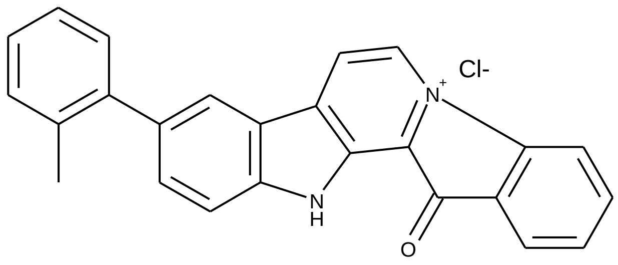

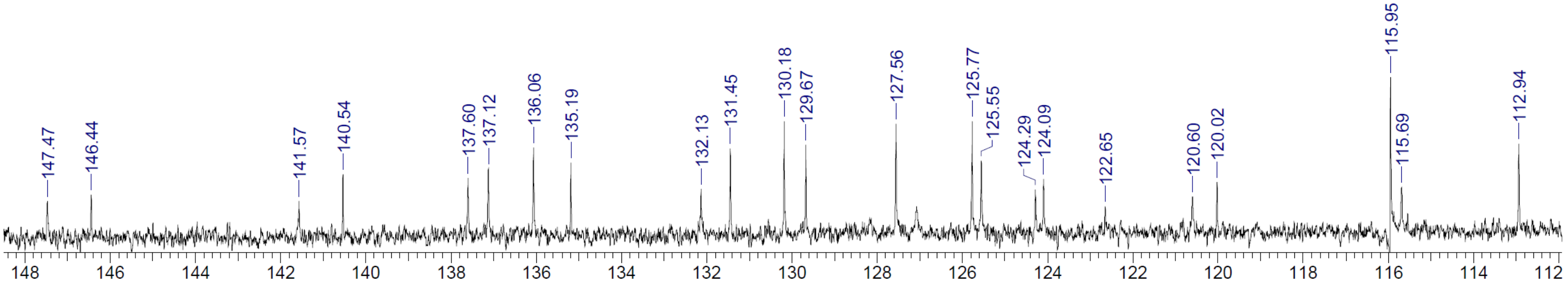

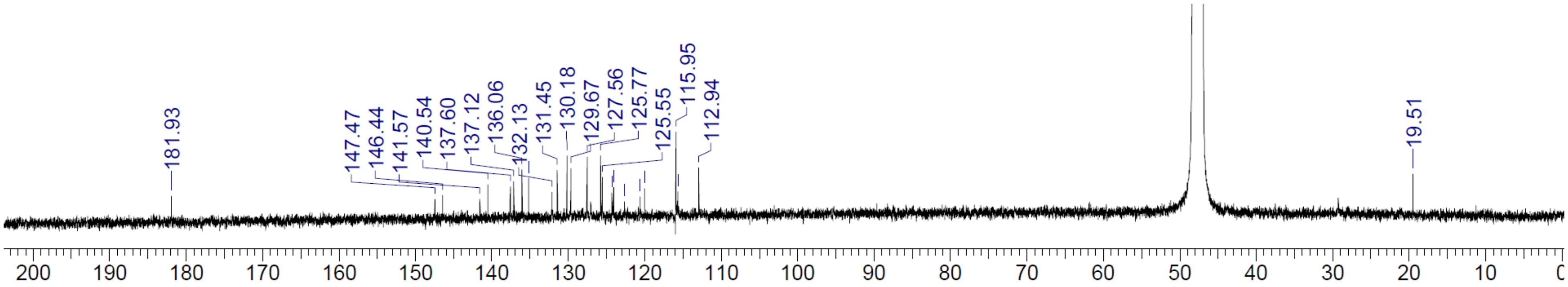

^{1}H NMR spectra of compound 25d

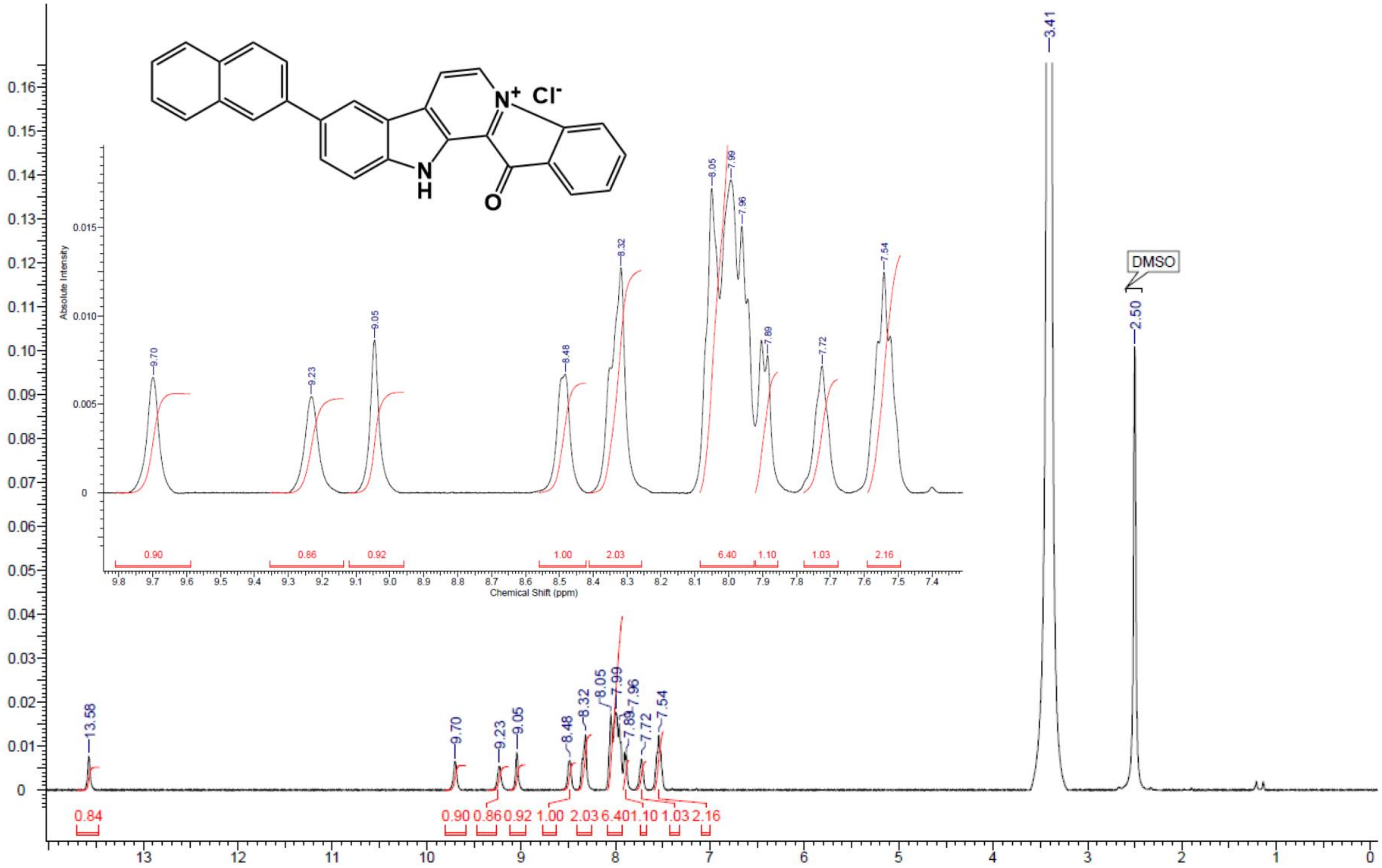

^{13}C NMR spectra of compound 25d

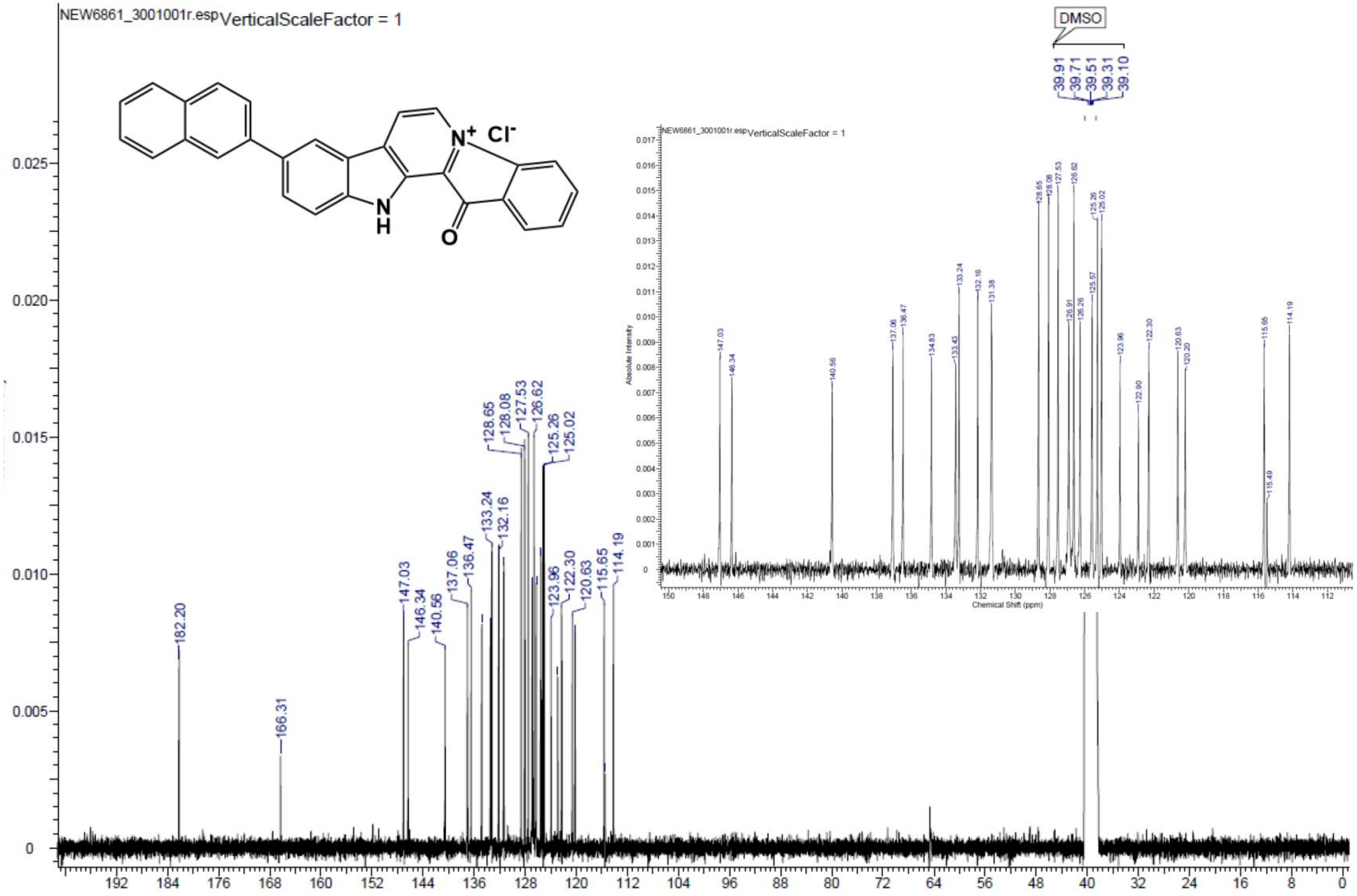

^{1}H NMR spectra of compound 25e

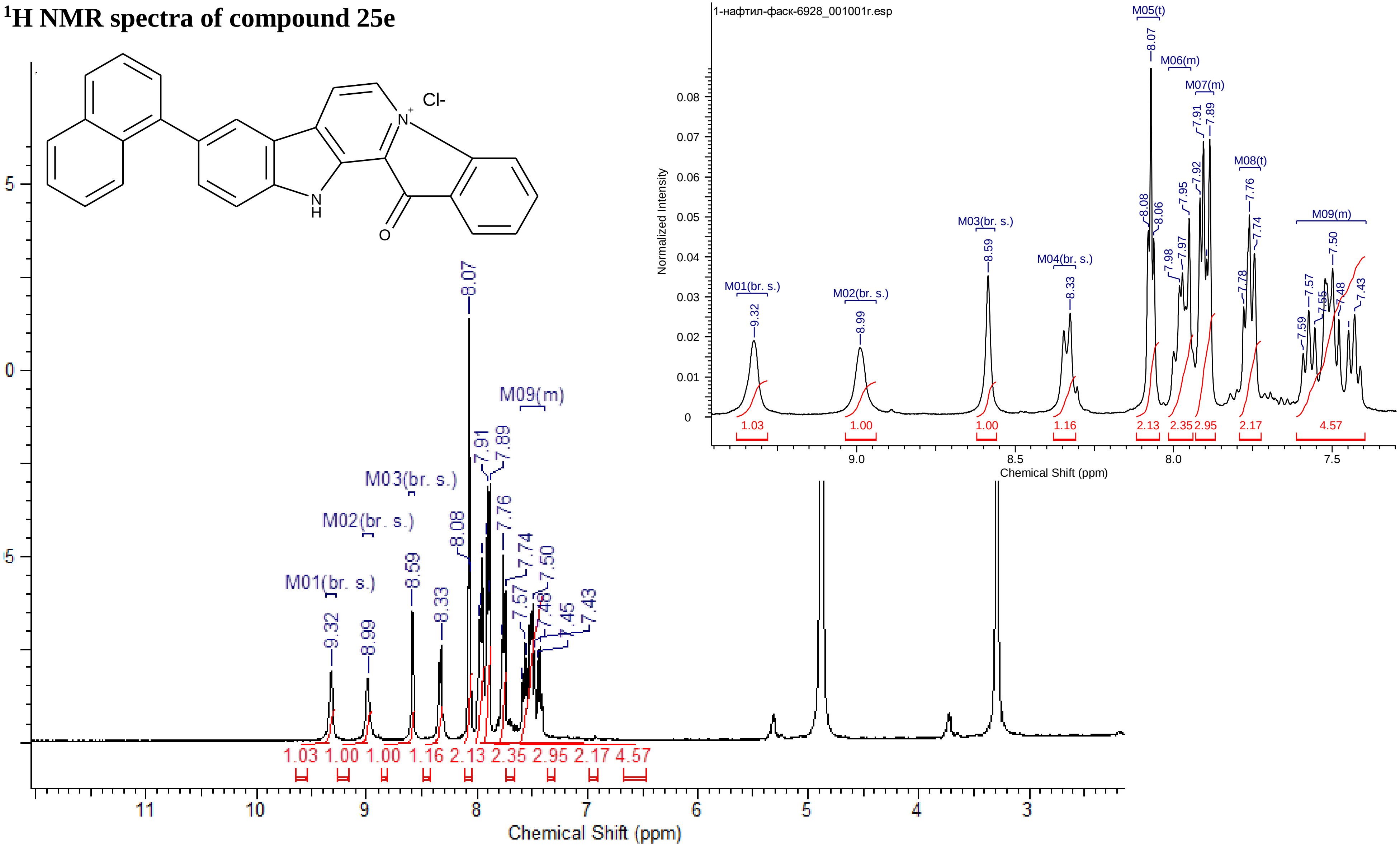

^{13}C NMR spectra of compound 25e

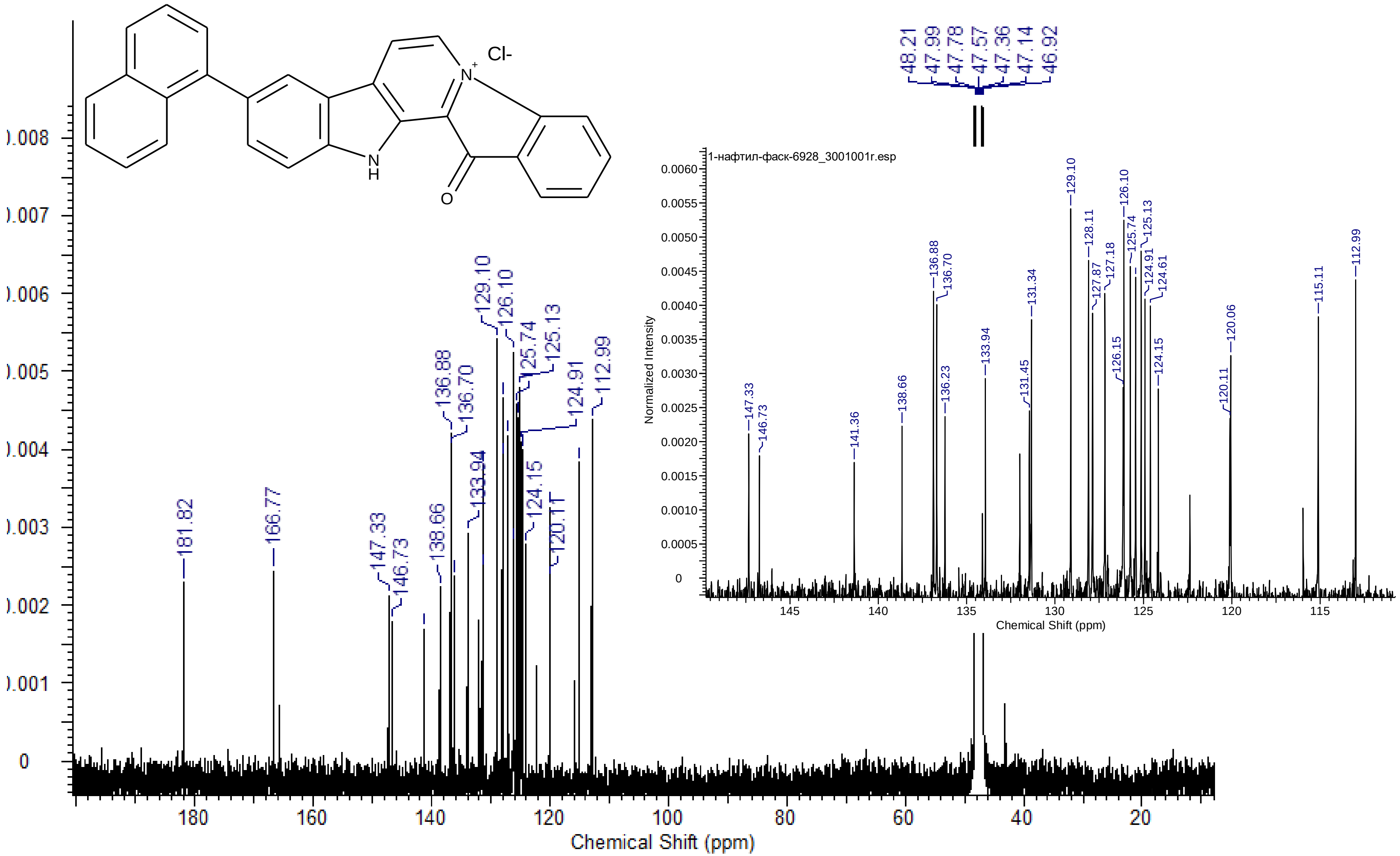

^{1}H NMR spectra of compound 25f

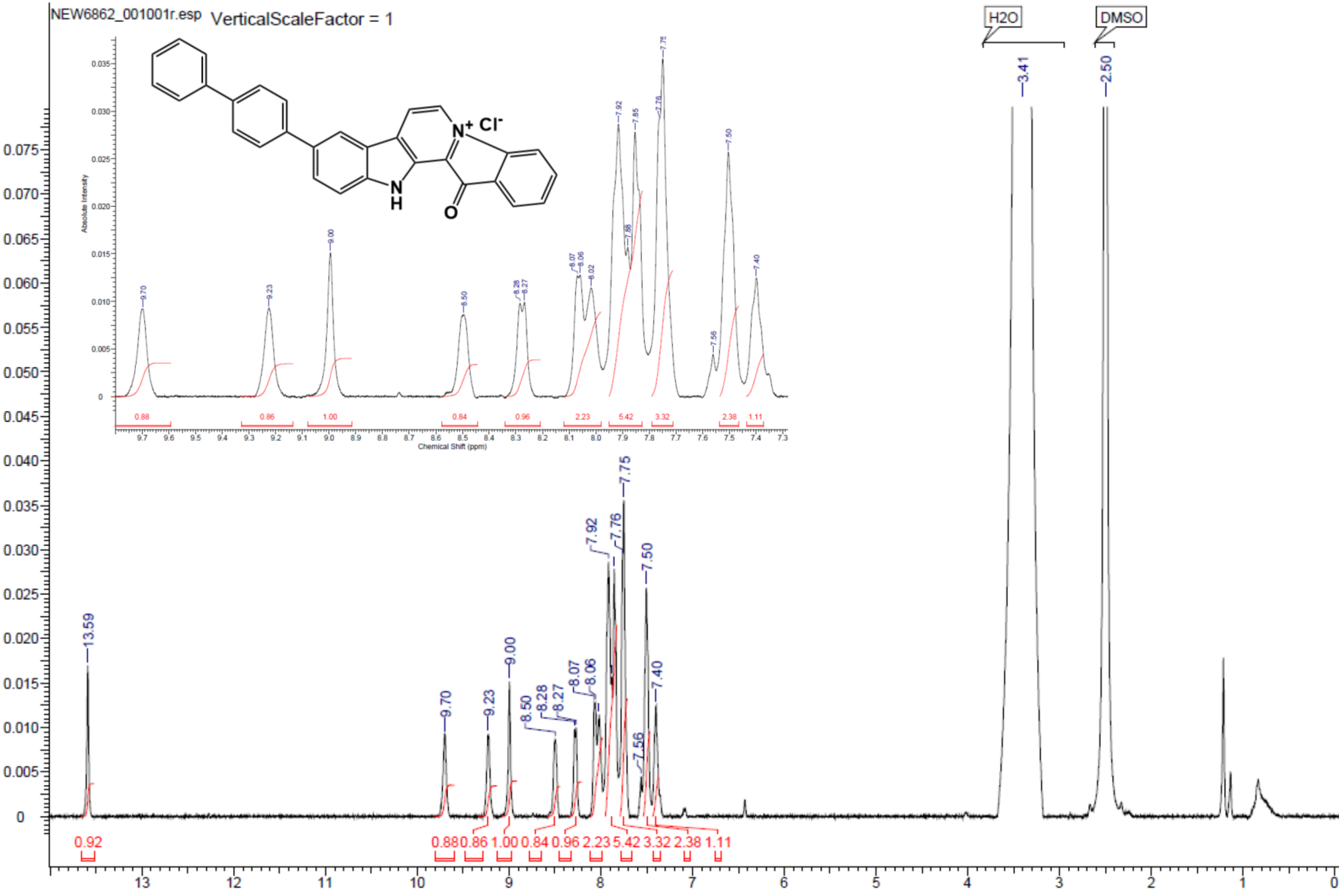

^{13}C NMR spectra of compound 25f

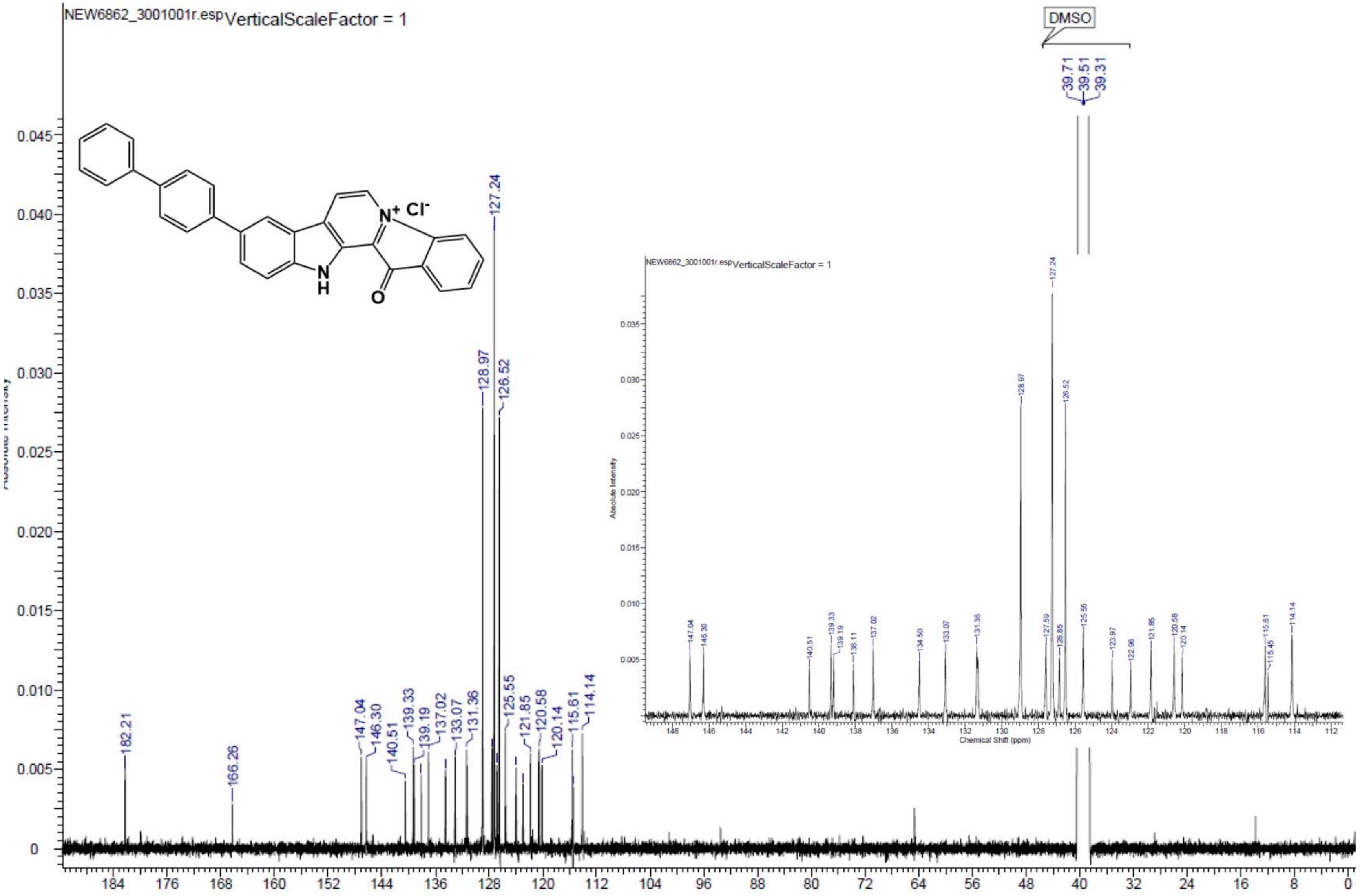

^{1}H NMR spectra of compound 25g

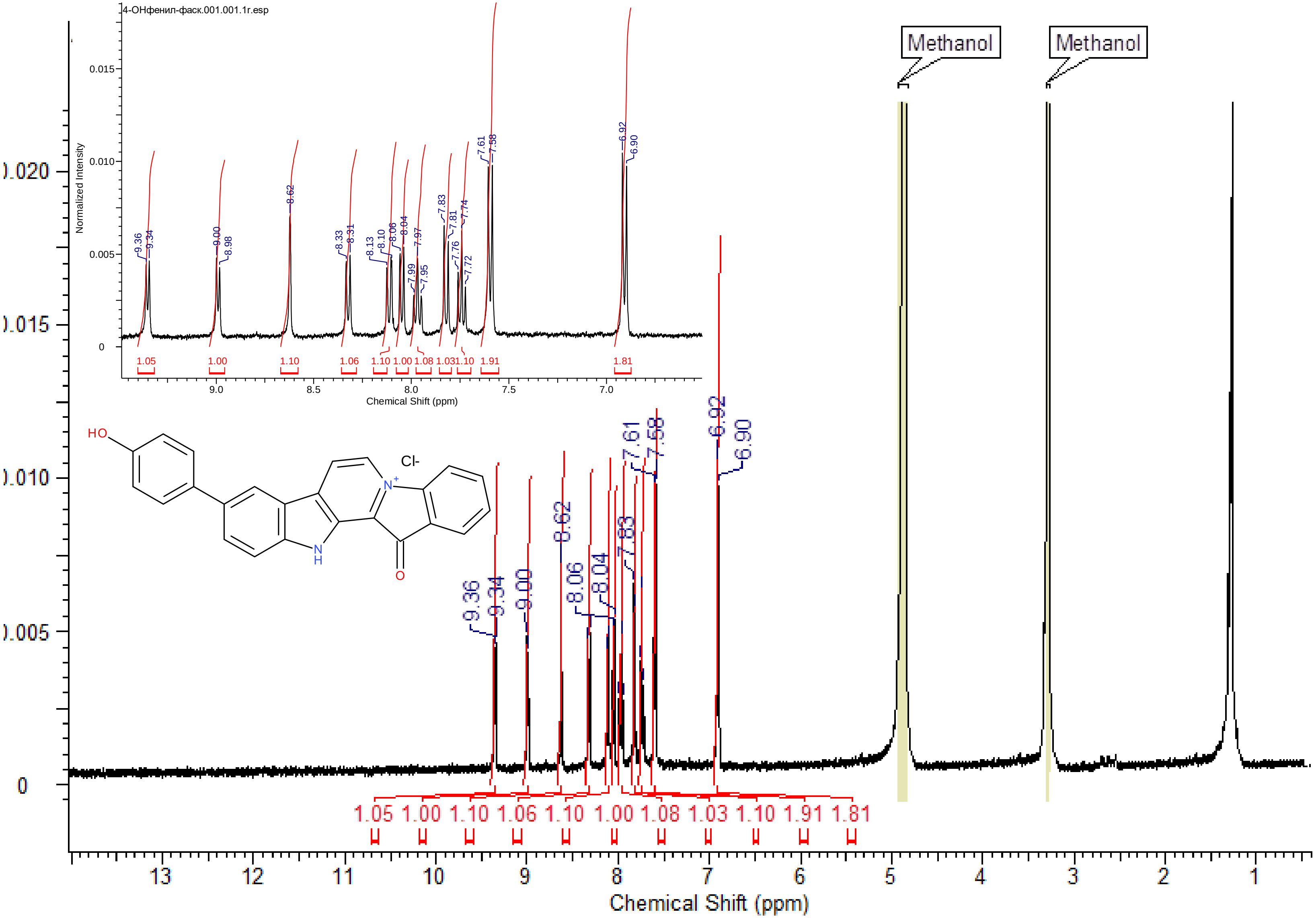

^{13}C NMR spectra of compound 25g

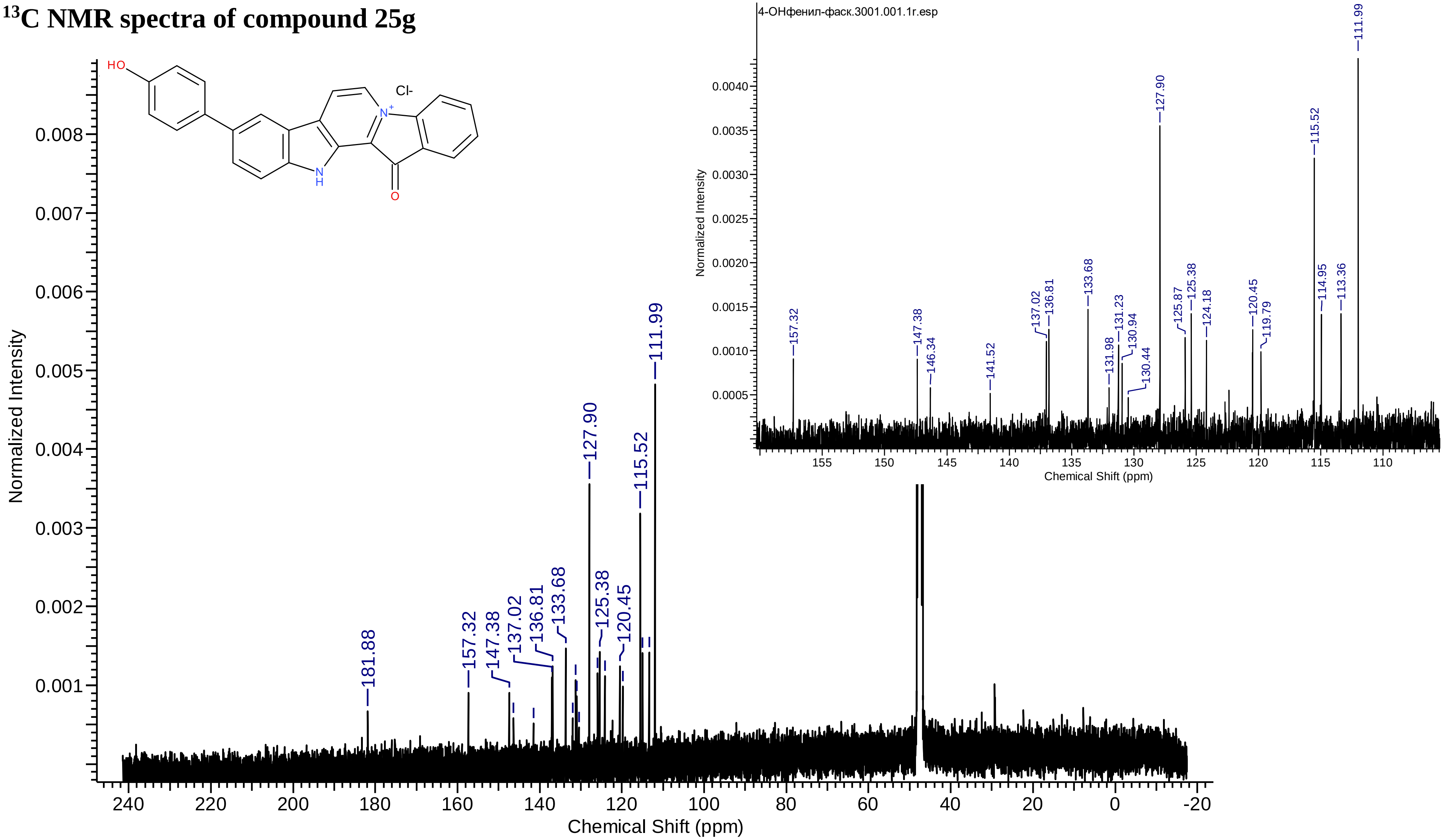

^{1}H NMR spectra of compound 25h

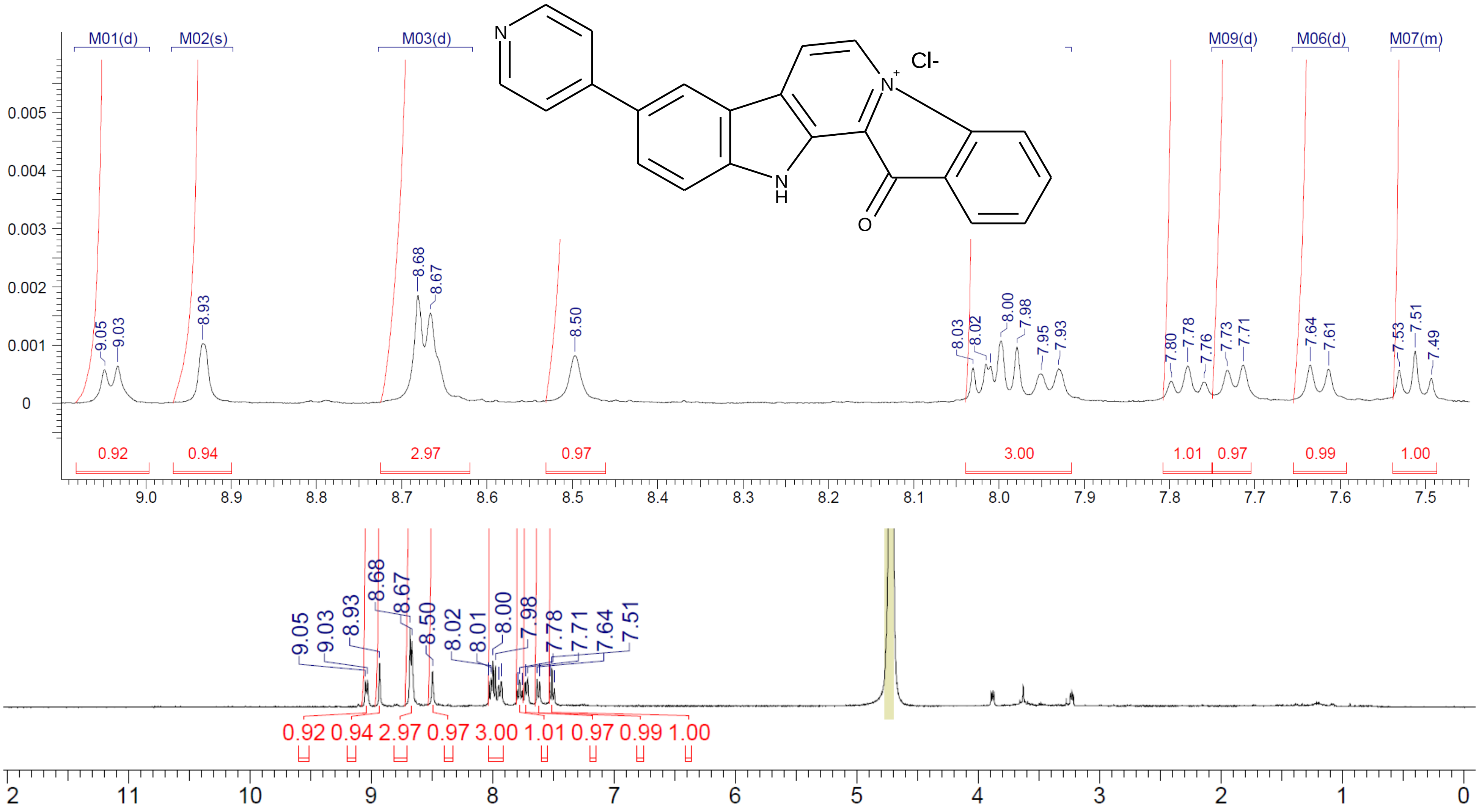

^{13}C NMR spectra of compound 25h

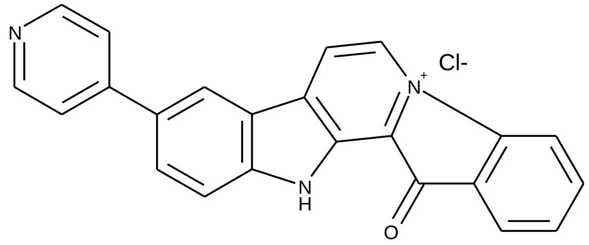

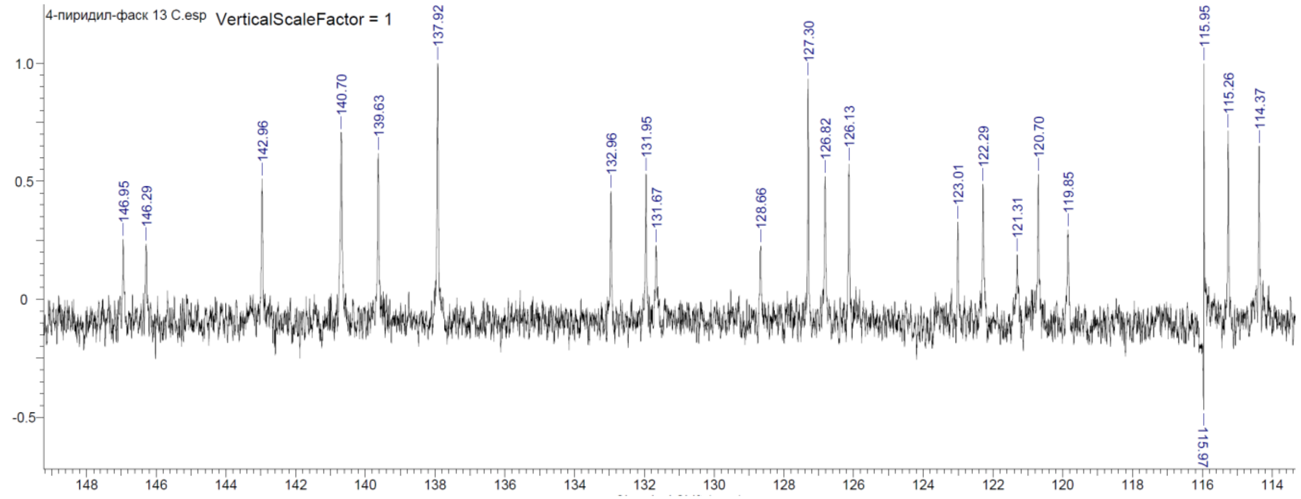

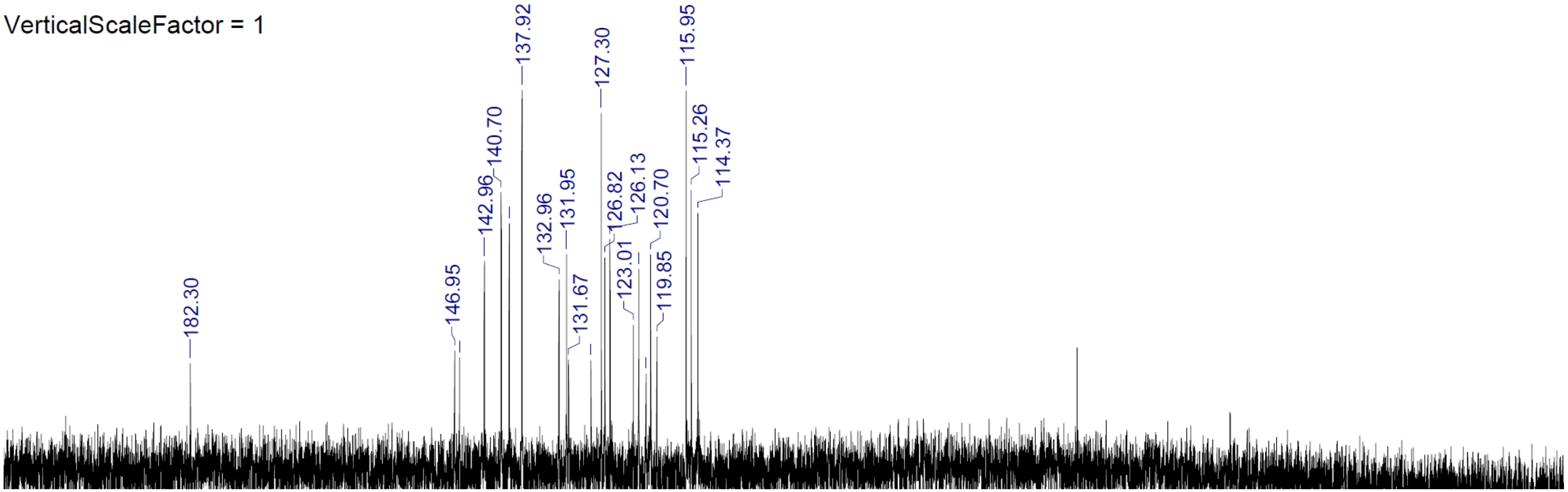

Supplement: Supplementary file 1 [file marinedrugs-22-00053-s001.zip › marinedrugs-2795641-supplementary.pdf]
